# Total Syntheses of 11‐Acetoxy‐4‐deoxyasbestinin D, 4‐Deoxyasbestinin C, Asbestinin‐10, ‐20, ‐21 and ‐23

**DOI:** 10.1002/chem.201904998

**Published:** 2020-01-16

**Authors:** Angus Campbell, Ian Mat Som, Claire Wilson, J. Stephen Clark

**Affiliations:** ^1^ School of Chemistry, Joseph Black Building University of Glasgow University Avenue Glasgow G12 8QQ UK

**Keywords:** dihydroxylation, diterpenes, epoxidation, natural products, oxepanes

## Abstract

Six members of the asbestinin family of marine diterpene natural products have been synthesized in an efficient and stereoselective manner from a single oxa‐bridged intermediate. Five of these natural products have not been synthesized previously and the structures of four of them have been confirmed as those proposed originally or following revisions to the original structures. The fifth natural product—asbestinin‐21—has been shown to be a diastereomer of the compound that had been proposed previously.

## Graphical abstract

**Access to the asbestinins**: The total syntheses of six members of the asbestinin family of marine diterpene natural products have been accomplished in an efficient and stereoselective manner from a single oxa‐bridged intermediate.

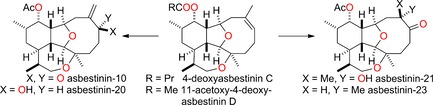

## Introduction

**Figure 1:**
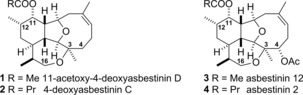
Examples of asbestinin natural products with and without an oxygen‐containing substituent at the C‐4 position.

The asbestinins are structurally complex tetracyclic marine diterpenes that have been isolated from the gorgonian octocorals *Briareum asbestinum* and *Briareum polyanthes*. The isolation and characterization of the first members of the family—asbestinins‐1–5—were reported by Faulkner, Clardy and co‐workers in 1980.^1^ The 39 asbestinins that have been isolated and characterized to date partition into two distinct structural groups: members that lack a C‐4 substituent, such as 11‐acetoxy‐deoxyasbestinin D (**1**) and 4‐deoxyasbestinin C (**2**), and those that are oxidised at C‐4, such as asbestinin‐12 (**3**) and asbestinin‐2 (**4**) (Figure 1).^2^, ^3^, ^4^, ^5^, ^6^, ^7^, ^8^ A biosynthetic route to the asbestinins from related briarellin natural products has been proposed in which Wagner–Meerwein rearrangement transfers a methyl group from C‐11 to C‐12.^1^, ^9^ The briarellin natural products are, in turn, thought to be derived from the simpler cladiellins (eunicellins).

Many of the asbestinins have been reported to possess significant biological activities: 4‐deoxyasbestinin A, 11‐acetoxy‐4‐deoxyasbestinin B, 4‐deoxyasbestinin C, 11‐acetoxy‐4‐deoxyasbestinin D and asbestinins‐6–10 possess significant in vitro activity against several cancer cells lines; *nor*‐asbestinin A, 11‐acetoxy‐4‐deoxyasbestinin F, and asbestinins‐10, ‐20, ‐21 and ‐26, display activity against the malarial parasite *Plasmodium falciparum*; 4‐deoxyasbestinin A, 11‐acetoxy‐4‐deoxyasbestinin B, 4‐deoxyasbestinin C, 11‐acetoxy‐4‐deoxy‐asbestinin D are active against the bacterium *Klebsiella pneumoniae*. However, the biological activities of most of the asbestinins have not been fully determined.^10^, ^11^

**Figure 2:**
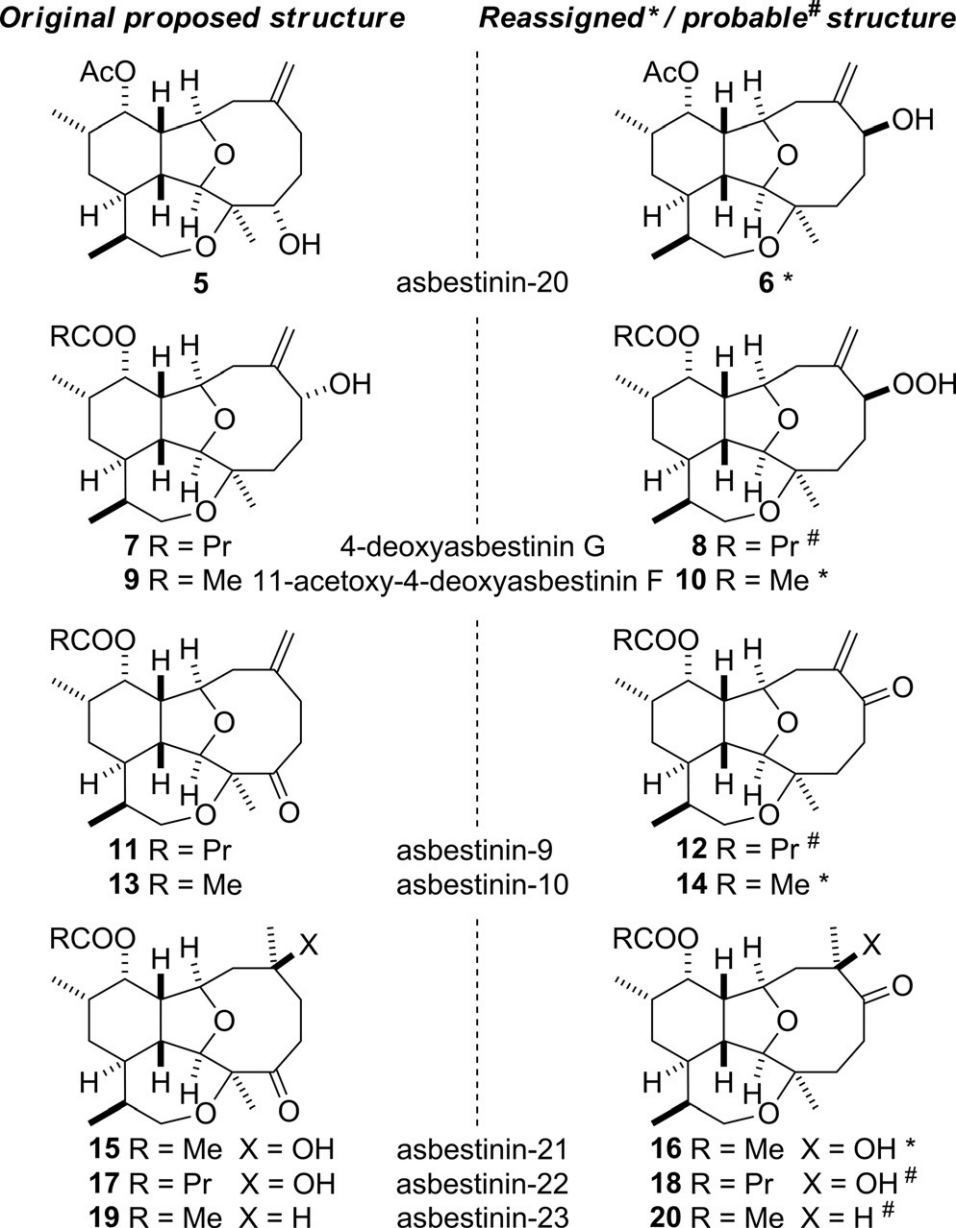
Asbestinin natural products that have had their structures corrected or are likely to require structural revision.

Several of the structures that were proposed for the asbestinins upon isolation have been revised as a consequence of the acquisition of better quality NMR data and the advent of new NMR techniques.^8^ Several asbestinins that were thought to be oxidized at the C‐4 position are likely to be 4‐deoxyasbestinins that contain a hydroxyl, hydroperoxy or carbonyl group at C‐6 instead (Figure 2). This work has resulted in tentative revision of the structures of asbestinin‐20 (**5**→**6**), 11‐acetoxy‐4‐deoxyasbestinin F (**9**→**10**), asbestinin‐10 (**13**→**14**) and asbestinin‐21 (**15**→**16**). It is likely that the structures of 4‐deoxyasbestinin G (**7**→**8**), asbestinin‐9 (**11**→**12**), asbestinin‐22 (**17**→**18**) and asbestinin‐23 (**19**→**20**) also require revision.^8^

The asbestinins possess a complex fused tetracyclic core that contains two medium‐sized cyclic ethers (ring sizes seven and nine) and bears a total of 9–12 stereogenic centres. The significant synthetic challenges presented by the asbestinins combined with their biological activities and the fact that there are ambiguities about their structures in many cases, makes them highly alluring targets for total synthesis. To date, 11‐acetoxy‐4‐deoxyasbestinin D and asbestinin‐12 are the only members of the asbestinin family of natural products that have been synthesized, as reported by Crimmins and Ellis in 2005 and 2008.^12^

## Results and Discussion

**Scheme 1:**
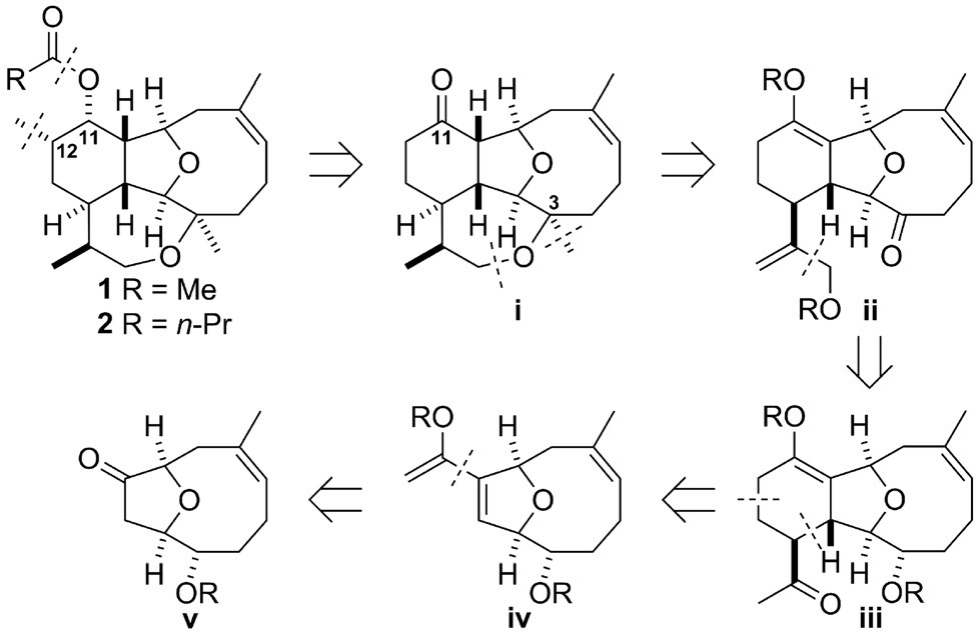
Retrosynthetic analysis of 11‐acetoxy‐4‐deoxyasbestinin D (**1**) and 4‐deoxyasbestinin C (**2**).

At the outset, we intended to synthesize 11‐acetoxy‐4‐deoxyasbestinin D (**1**) and 4‐deoxyasbestinin C (**2**), and prepare several other asbestinins from these natural products thereafter.^3^ The retrosynthetic analysis of the natural products **1** and **2** began with the cleavage of the ester and formation of a ketone at C‐11 and removal of the C‐12 methyl substituent to give advanced tetracyclic intermediate **i** (Scheme 1). Sequential opening of the oxepane by scission of the C−O bond distal to the quaternary centre at C‐3, conversion of the methyl substituent into a methylene group and the C‐11 ketone into an enol ether, followed by removal of the C‐3 methyl substituent led to the tricyclic ketone **ii**. Subsequent conversion of the side‐chain allylic ether into a methyl ketone produced intermediate **iii**, an analogue of a synthetic intermediate used in our total syntheses of members the cladiellin natural products.^13^ Further Diels–Alder disconnection led to the diene **iv**, and disconnection of the diene revealed the bridged bicyclic ether **v**.

**Scheme 2:**
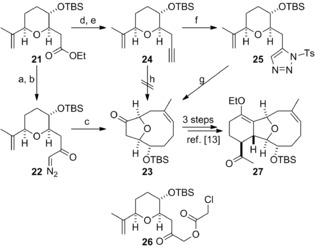
Oxonium ylide formation and ring expansion to form the oxa‐bridged tricyclic core system. a) LiOH, EtOH‐H_2_O (3:1), RT, 86 %; b) *i*BuOCOCl, Et_3_N, RT, then CH_2_N_2_, Et_2_O, RT, 82 %; c) Cu(hfacac)_2_, CH_2_Cl_2_, reflux, 90 % (5:1, *Z*:*E*); d) *i*Bu_2_AlH, CH_2_Cl_2_, −78 °C; e) MeCOC(N_2_)P(O)(OMe)_2_, K_2_CO_3_, MeOH, RT, 86 % (2 steps); f) *n*BuLi. TsN_3_, THF, −78 °C, 87 %; g) Rh_2_(OAc)_4_, DCE, reflux, 48 % (>20:1, *Z*:*E*); h) SPhosAuNTf_2_, ClCH_2_CO_2_H, 5‐bromo‐2‐methyl‐pyridine‐*N*‐oxide, DCE, RT, 36 % (**26**).

The ketone corresponding to tricyclic intermediate **iii** in Scheme 1 was prepared as shown in Scheme 2. Several approaches to the synthesis of the bridged bicyclic ketone **23** (a compound corresponding to ketone **v** in Scheme 1) from tetrahydropyranyl ester **21** were explored. In the first approach, we employed the method that we had used in our cladiellin syntheses.^13^ Conversion of ester **21** into α‐diazo ketone **22** proceeded smoothly and subsequent sequential copper‐catalysed carbenoid generation, ylide formation and rearrangement delivered the oxa‐bridged bicyclic ketone **23** in high yield. In the second approach, the metal‐mediated reaction of triazole **25** according to conditions described by Boyer was explored.^14^ Ester **21** was subjected to reduction to give the corresponding aldehyde followed by Bestmann–Ohira homologation to deliver alkyne **24**.^15^ Deprotonation of alkyne **24** and reaction of the resulting anion with tosyl azide afforded triazole **25**.^14^ The rhodium‐mediated reaction of triazole **25** produced the expected ketone **23** with a high level of diastereocontrol but in lower yield than that obtained from the copper‐catalysed reaction of α‐diazo ketone **22**. Finally, direct oxidative gold‐catalysed cyclization of alkyne **24** was performed in an attempt to generate the bicyclic oxonium ylide directly from this substrate and thus obviate the use of either α‐diazo ketone **22** or triazole **25**.^16^ Unfortunately, ester **26** was obtained instead of the required rearrangement product **23**.

**Scheme 3:**
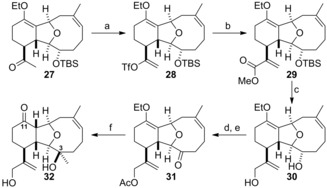
Functionalised of the tricyclic core in preparation for construction of the oxepane. a) NaN(SiMe_3_)_2_, PhNTf_2_, THF, −78 °C; b) Pd(PPh_3_)_4_, CO, MeOH, *i*Pr_2_NEt, RT, 96 % (2 steps); c) *i*Bu_2_AlH, PhMe, −35 °C, 80 %; d) Ac_2_O, DMAP (4‐dimethylaminopyridine), Et_3_N, CH_2_Cl_2_, RT; e) Dess–Martin periodinane, pyridine, CH_2_Cl_2_, RT, 98 % (2 steps); f) MeMgCl, THF, 0 °C→RT then 1 m HCl aq., RT, 87 %.

Bicyclic ether **23** was converted into tricyclic ketone **27**, as reported previously.^13^ Further functionality was introduced to enable the construction of the oxepane, the final ring in the tetracyclic ensemble (Scheme 3). One‐carbon chain extension was performed by conversion of the methyl ketone into triflate **28** followed by palladium‐mediated carbonylation in methanol to deliver ester **29** in excellent yield. Selective reduction of the carbonyl group of the α,β‐unsaturated ester was accompanied by the loss of the TBS (*tert*‐butyldimethylsilyl ether) protecting group, and diol **30** was produced in high yield. Selective acetylation of the primary allylic alcohol and subsequent oxidation of the free secondary hydroxyl group delivered ketone **31**. Treatment of this ketone with a large excess of methylmagnesium chloride resulted in a highly stereoselective installation of the C‐3 methyl substituent and concurrent cleavage of the acetate group.^17^ The enol ether was converted into the corresponding C‐11 ketone by hydrolysis during the acidic workup and keto diol **32** was produced in excellent yield.

**Scheme 4:**
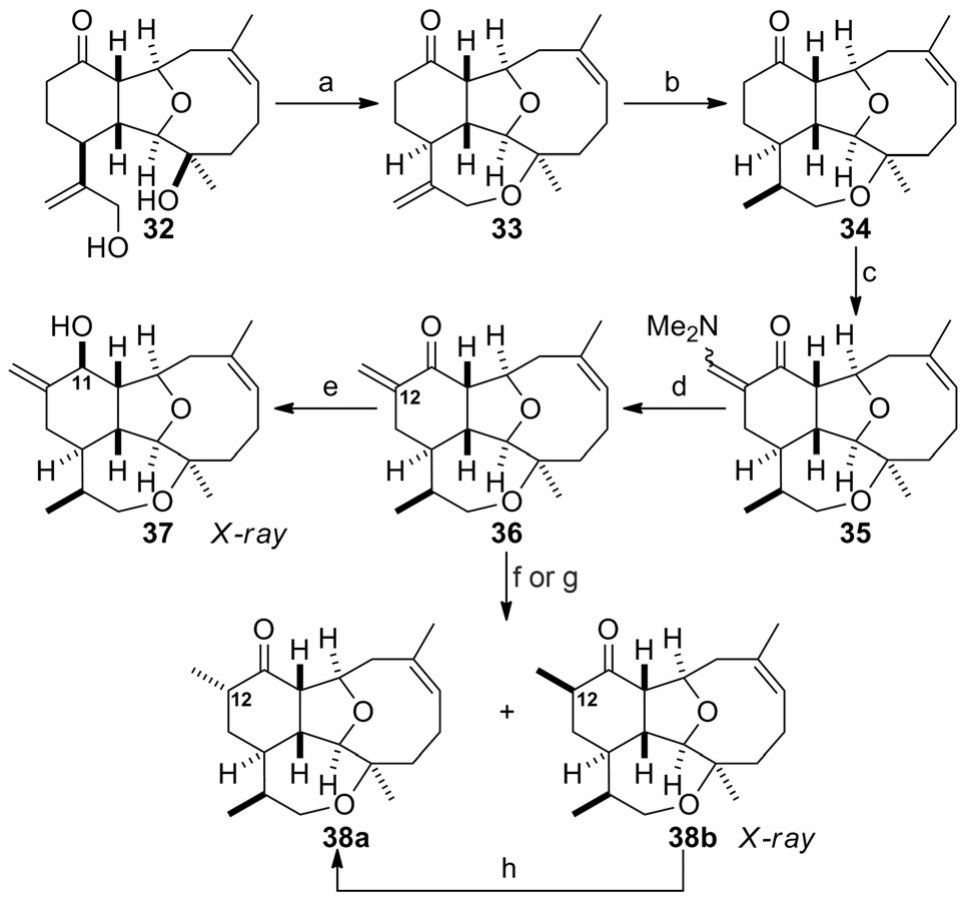
Completion of the tetracyclic asbestinin core. a) Tf_2_O, 2,6‐lutidine, CHCl_3_, 0 °C, 97 %; b) H_2_, PtO_2_, EtOAc, RT, 90 %; c) *t*BuOCH(NMe_2_)_2_, DMF, 100 °C; d) *i*Bu_2_AlH, CH_2_Cl_2_, −78 °C then MeI, RT, 52 % (2 steps); e) NaBH_4_, CeCl_3_⋅7 H_2_O, MeOH, −78 °C, 49 % (3 steps); f) L‐Selectride, THF, −78 °C, 76 % (30 % **38 a**+46 % **38 b**); g) [CuH(PPh_3_)]_6_, PhMe, −78 °C, 62 % (25 % **38 a**+37 % **38 b**) (3 steps); h) K_2_CO_3_, MeOH, RT, 85–99 % (1:1.4, **38 a**:**38 b**).

Conversion of tricyclic diol **32** into the fully functionalised tetracyclic asbestinin framework was accomplished by following the route shown in Scheme 4. Oxepane formation was performed by treatment of diol **32** with triflic anhydride in the presence of 2,6‐lutidine. The primary allylic triflate was not isolated from this reaction and instead the required cyclic allylic ether **33** was obtained in excellent yield as a result of immediate intramolecular nucleophilic displacement of the triflate by the tertiary alcohol. Hydrogenation of allylic ether **33** in the presence of Adams’ catalyst was both chemoselective and stereoselective; the tetracyclic ketone **34** was obtained as a single diastereomer in high yield.^18^ To convert ketone **34** into the natural products **1** and **2** it was necessary to install the C‐12 methyl substituent, reduce the ketone and esterify the resulting alcohol. Direct methylation at C‐12 by ketone deprotonation and alkylation was low‐yielding and non‐stereoselective, so other methods for the introduction of the methyl substituent were explored. Reaction of ketone **34** with Bredereck's reagent^19^ at 100 °C in DMF produced enamine **35**, which was subjected to immediate reduction with DIBAL‐H to afford enone **36**. Reduction of this enone under Luche conditions produced the crystalline allylic alcohol **37** as a single diastereomer, but the configuration at the hydroxyl‐bearing stereogenic centre (C‐11) was not the one required for the synthesis of the asbestinins, as revealed by X‐ray analysis.^20^, ^21^ Treatment of enone **36** with either L‐Selectride or Stryker's reagent^22^ resulted in the formation of a diastereomeric mixture of the required ketone **38 a** and its C‐12 epimer **38 b**, in which the latter predominated marginally. The epimeric ketones were separated and equilibration of each ketone under mildly basic conditions delivered a mixture of diastereomers in a similar ratio to that obtained by conjugate reduction of enone **36**.

**Scheme 5:**
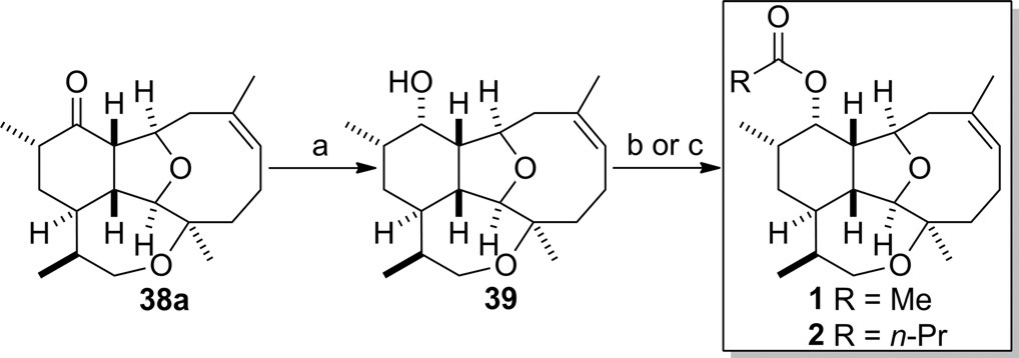
Completion of the syntheses of 11‐acetoxy‐4‐deoxyasbestinin D (**1**) and 4‐deoxyasbestinin C (**2**). a) NaBH_4_, CH_2_Cl_2_‐MeOH (1:1), RT; b) Ac_2_O, DMAP, Et_3_N, CH_2_Cl_2_, RT, 88 % (2 steps); c) (*n*‐PrCO)_2_O, DMAP, Et_3_N, CH_2_Cl_2_, RT, 50 % (2 steps).

Completion of the total syntheses of 11‐acetoxy‐4‐deoxyasbestinin D (**1**) and 4‐deoxyasbestinin C (**2**) was finally possible (Scheme 5). Reduction of ketone **38 a** to give the alcohol by treatment with sodium borohydride was highly diastereoselective.^23^ Immediate acetylation of the resulting alcohol **39** provided 11‐acetoxy‐4‐deoxyasbestinin D (**1**) in excellent yield and reaction of alcohol **39** with butyric anhydride produced 4‐deoxyasbestinin C (**2**) in good yield. The NMR and other characterization data of the synthetic esters **1** and **2** matched the data reported for each natural product.

**Scheme 6:**
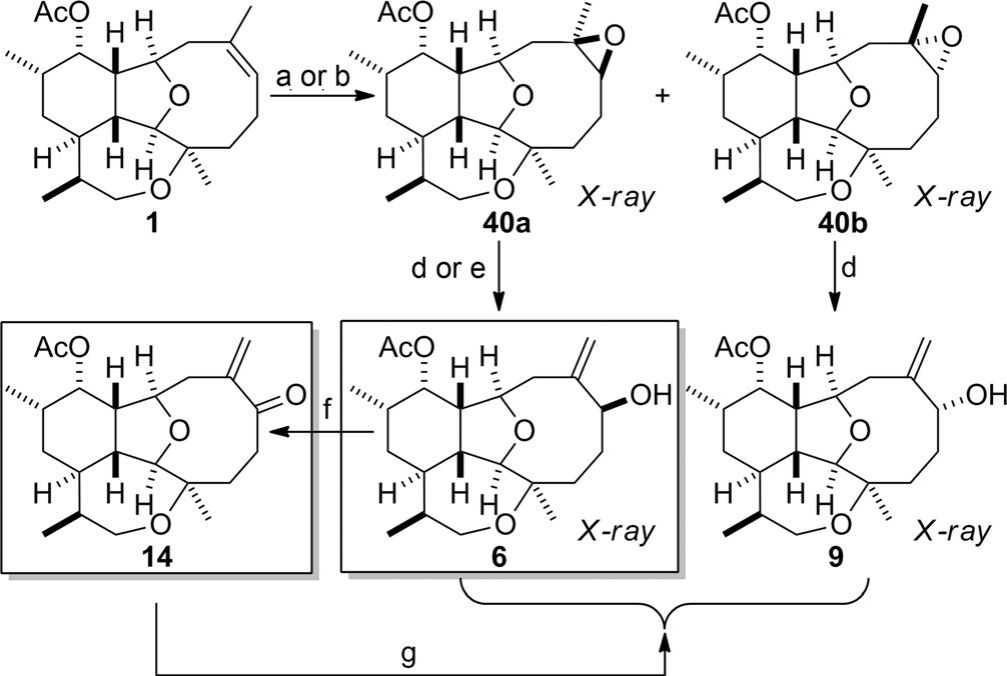
Synthesis of asbestinin‐20 (**6**), 6‐*epi*‐asbestinin‐20 (**9**) and asbestinin‐10 (**14**). a) *m*CPBA, CH_2_Cl_2_, −10 °C, 82 % (59 % **40 a**+23 % **40 b**); b) (+)‐Shi epoxidation catalyst, oxone, K_2_CO_3_, *n*Bu_4_NHSO_4_, Na_2_B_4_O_7_×10H_2_O, MeCN‐DMM (1:2) 0 °C, 81 % (75 % **40 a**+6 % **40 b**); c) (−)‐Shi epoxidation catalyst, oxone, K_2_CO_3_, *n*Bu_4_NHSO_4_, Na_2_B_4_O_7_×10H_2_O, MeCN‐DMM (1:2) 0 °C, 84 % (13 % **40 a**+71 % **40 b**); d) *t*BuMe_2_SiOTf, 2,6‐lutidine, CH_2_Cl_2_, 0 °C then TBAF, THF, RT, 39 % (**6**), 64 % (**9**); e) Au‐TiO_2_, DCE, 80 °C, 35 %; f) Dess–Martin periodinane, pyridine, CH_2_Cl_2_, RT, 75 %; g) NaBH_4_, CeCl_3_⋅7 H_2_O, CH_2_Cl_2_‐MeOH (1:1), −78 °C, 84 % (64 % **6**+20 % **9**).

Several other asbestinin natural products were synthesized from 11‐acetoxy‐4‐deoxyasbestinin D (**1**) in just two or three steps. Of particular interest was the synthesis of asbestinin‐20 (**6**) to verify its reassigned structure (Scheme 6). 11‐Acetoxy‐4‐deoxyasbestinin D (**1**) was first epoxidized by treatment with *m*CPBA to give a mixture (2:1) of diastereomeric epoxides **40 a** and **40 b**. The crystalline epoxides were readily separable by chromatography and their structures were determined by X‐ray crystallography.^20^ To amplify or override the weak substrate bias and thereby generate epoxides **40 a** and **40 b** with higher levels of diastereocontrol, the epoxidation reaction was performed by the application of Shi's asymmetric protocol using both enantiomers of the fructose‐derived catalyst.^24^ In the case of the (+)‐catalyst, the relatively weak substrate bias was reinforced and the ratio of diastereomers improved to 10.5:1 (**40 a**:**40 b**). In the mismatched case, in which the (−)‐catalyst was employed, the substrate bias was overturned and a 1:5.5 ratio of diastereomers (**40 a**:**40 b**) was obtained. Epoxides **40 a** and **40 b** were then converted into the corresponding allylic alcohols by sequential reaction with *tert*‐butyldimethylsilyl triflate in the presence of 2,6‐lutidine and treatment with TBAF.^25^ The crystalline allylic alcohols **6** and **9** were obtained with yields of 39 and 64 %, respectively; their structures were confirmed by X‐ray crystallography.^20^ In the case of epoxide **40 a**, treatment with titania‐supported gold nanoparticles in dichloroethane (DCE) at 80 °C, according to the procedure described by Garcia and Stratakis,^26^ delivered allylic alcohol **6** in 35 % yield. Comparison of NMR and other characterization data of alcohols **6** and **9** with that of natural asbestinin‐20 confirmed that the natural product is compound **6**. Thus, the reassigned structure and relative stereochemistry of asbestinin‐20 published by Ospina and Rodríguez is correct.^8^ Dess–Martin oxidation of asbestinin‐20 (**6**) produced asbestinin‐10 (**14**) and the structure of this natural product was confirmed. Enone reduction under Luche conditions afforded the allylic alcohols in a 3.2:1 ratio favouring asbestinin‐20 (**6**), a result consistent with that reported by Ospina and Rodríguez when they reduced natural asbestinin‐10 (**14**) under similar conditions.^8^

**Scheme 7:**
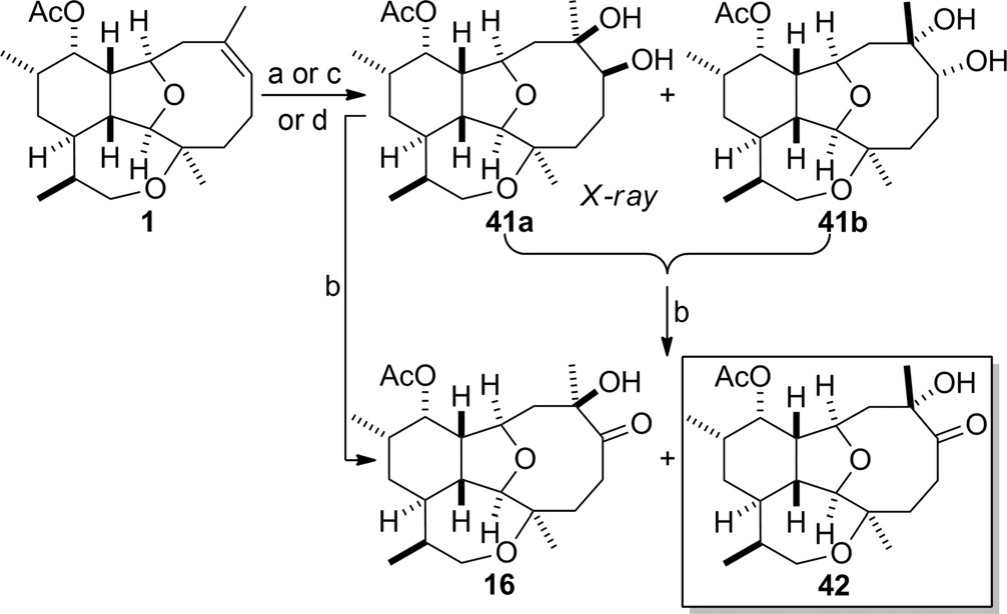
Stereoselective dihydroxylation of 11‐acetoxy‐4‐deoxyasbestinin D (**1**) and synthesis of asbestinin‐21 (**42**) and 7‐*epi*‐asbestinin‐21 (**16**). a) OsO_4_, NMO (*N*‐methylmorpholine *N*‐oxide), THF‐H_2_O (1:1), RT, 56 % (5:1, **41 a**:**41 b**); b) Dess–Martin periodinane, pyridine, CH_2_Cl_2_, RT, 87 % (**41 a→16**), 81 % (**41 a**+**41 b→16**+**42**); c) AD‐mix‐α, *t*BuOH‐H_2_O (1:1), 0 °C, 23 % (>12:1, **41 a**:**41 b**)+46 % (**16**); d) AD‐mix‐β, *t*BuOH‐H_2_O (1:1), 0 °C, 75 % (8:1, **41 a**:**41 b**)+10 % (**16**).

The next target to be synthesized was asbestinin‐21, which had been originally identified as the ketone **15** (Figure 2), but was later reassigned to be the α‐hydroxy ketone **16** (Scheme 7).^6^, ^8^ Dihydroxylation of 11‐acetoxy‐4‐deoxyasbestinin D (**1**) under standard Upjohn dihydroxylation conditions afforded a diastereomeric mixture of 1,2‐diols **41 a** and **41 b** in 56 % yield, in which the crystalline diol **41 a** predominated (5:1 ratio).^20^ The diol **41 a** was separated from the minor isomer **41 b** and oxidized to give α‐hydroxy ketone **16** by the use of the Dess–Martin protocol. Alternatively, the diastereomeric mixture of diols **41 a** and **41 b** was oxidized directly to produce a separable mixture of α‐hydroxy ketones **16** (67 % yield) and **42** (14 % yield).

Attempts were made to produce diol **41 b** as the major diastereomer by performing dihydroxylation according to the Sharpless asymmetric protocol.^27^ The use of AD‐mix‐α to perform the dihydroxylation reaction of 11‐acetoxy‐4‐deoxyasbestinin D (**1**) resulted in the formation of diol **41 a** with an enhanced level of diastereocontrol (>12:1). However, diol **41 a** was obtained in only 23 % yield, and the major product was the α‐hydroxy ketone **16** (46 % yield), an unexpected product resulting from over‐oxidation. In the case where substrate control and reagent control were expected to be mismatched, dihydroxylation of alkene **1** with AD‐mix‐β produced a mixture of diols **41 a** and **41 b** (75 % yield combined) along with a small amount (10 % yield) of α‐hydroxy ketone **16**. Interestingly, the dihydroxylation reaction proceeded with a higher level of diastereocontrol (8:1) than for the case when the reaction was performed under simple Upjohn conditions. This unexpected result presumably reflects the larger steric bulk of the osmium complex in the case of AD‐mix and the fact the reaction proceeds under substrate control rather than reagent control. Comparison of the ^1^H and ^13^C NMR data of the α‐hydroxy ketones **16** and **42** with those of natural asbestinin‐21 revealed that the natural product is **42** rather than **16**, a finding which established that the configuration of the hydroxyl‐bearing the C‐7 stereogenic centre had been misassigned by Ospina and Rodríguez when performing their structural revision.^8^

**Scheme 8:**
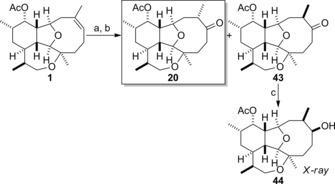
Completion of the syntheses of asbestinin‐23 (**20**) and 7‐*epi*‐asbestinin‐23 (**43**). a) BH_3_⋅THF, THF, 0 °C then NaBO_3_⋅4 H_2_O; b) Dess–Martin periodinane, pyridine, CH_2_Cl_2_, RT, 69 % (2 steps) (49 % **43 a**, 20 % **20**); c) L‐Selectride, THF, −78 °C, 64 % (>20:1).

The last natural product to be synthesized in our study was asbestinin‐23.^6^, ^8^ Reaction of 11‐acetoxy‐4‐deoxyasbestinin D (**1**) with borane‐THF complex followed by oxidative work‐up produced an inseparable diastereomeric mixture of alcohols (Scheme 8). This mixture of alcohols was subjected to immediate oxidation with Dess–Martin periodinane to give ketones **20** and **43**, which could be separated and were isolated in yields of 20 and 49 %, respectively. Reduction of ketone **43** with L‐Selectride afforded alcohol **44**, a crystalline solid, in a highly stereoselective manner (>20:1 selectivity). The configurations of the stereogenic centres at C‐6 and C‐7 of alcohol **44** were determined by X‐ray crystallography,^20^ and the configuration of the C‐7 stereocentres of ketones **43** and **20** were established consequently. Comparison of NMR data of ketones **20** and **43** with the data of the natural asbestinin‐23 revealed that the natural product is ketone **20**, which was suggested by Ospina and Rodríguez as the most likely alternative to the originally assigned structure (**19**, Figure 2).^8^

## Conclusions

We have completed the stereoselective total syntheses of six members of the asbestinin family of marine diterpene natural products from the bridged‐bicyclic ether **23**, which had been prepared previously from readily available starting materials. Syntheses of 11‐acetoxy‐deoxyasbestinin D (**1**) and 4‐deoxyasbestinin C (**2**) have been completed in 16 steps from the bicyclic ketone **23** and the former has been converted into abestinin‐10, abestinin‐20, abestinin‐21 and abestinin‐23 in two or three additional steps. The syntheses of abestinin‐10, abestinin‐20, and abestinin‐23 confirm the revised structures for these compounds proposed by Ospina and Rodríguez in 2006. In the case of abestinin‐21, our work has shown that this compound is the C‐7 epimer of the revised structure proposed by Ospina and Rodríguez.

## Conflict of interest

The authors declare no conflict of interest.

## Supporting information

As a service to our authors and readers, this journal provides supporting information supplied by the authors. Such materials are peer reviewed and may be re‐organized for online delivery, but are not copy‐edited or typeset. Technical support issues arising from supporting information (other than missing files) should be addressed to the authors.

Supplementary
