# Supplementary material for: Total Syntheses of 11‐Acetoxy‐4‐deoxyasbestinin D, 4‐Deoxyasbestinin C, Asbestinin‐10, ‐20, ‐21 and ‐23
Source: Chemistry. 2020 Jan 16;26(5):1155–60. doi: 10.1002/chem.201904998 (PMC7027880; doi:10.1002/chem.201904998)

CHEMISTRY
A European Journal

Supporting Information

Total Syntheses of 11-Acetoxy-4-deoxyasbestinin D, 4-Deoxyasbestinin C, Asbestinin-10, -20, -21 and -23

Angus Campbell, Ian Mat Som, Claire Wilson, and J. Stephen Clark*[a]

chem_201904998_sm_miscellaneous_information.pdf

Table of Contents

General Experimental S1

Experimental Procedures S2

References S31

^{1}H and ^{13}C NMR Spectra for New Compounds S32

General Experimental

Reagents and solvents were purchased from commercial suppliers and were used without further purification, unless otherwise stated.

Air and/or moisture sensitive reactions were performed under an atmosphere of argon in flame-dried apparatus. THF, toluene, acetonitrile, dichloromethane and diethyl ether were purified using a Pure-SolvTM 500 Solvent Purification System. Reactions were monitored by thin layer chromatography (TLC) using Merck silica gel 60 covered aluminium backed plates F254. TLC plates were visualised under UV light and stained using potassium permanganate solution or acidic ethanolic anisaldehyde solution. Flash column chromatography was performed with silica gel (Geduran Si 60 35-70 μm, purchased from Merck and SilicaGel 60A 40-63 μm, purchased from Fluorochem) as the solid support. Petroleum ether used for column chromatography was the 40–60 °C fraction.

IR spectra were recorded as thin films employing a Shimadzu FTIR-8400S spectrometer equipped with a Pike Technologies MIRacle ATR accessory; selected frequencies (ν_{max}) are reported. NMR spectra were recorded using dilute solutions in $CDCl_3$ or C_6D_6 on a Bruker AvanceIII 400 MHz, or Bruker AvanceIII UltraShield 500 MHz spectrometer using the deuterated solvent as the internal deuterium lock. ^{1}H Chemical shift data are given in units δ relative to the residual protic solvent where δ ($CDCl_3$) = 7.26 ppm and δ (C_6D_6) = 7.16. ^{1}H signals are described as singlets (s), doublets (d), triplets (t), quartets (q), multiplets (m), broad (br), apparent (app) or a combination of these. ^{13}C Chemical shift data were recorded with broadband proton decoupling and are given in units δ relative to the solvent where δ ($CDCl_3$) = 77.16 ppm and δ (C_6D_6) = 128.1. High resolution mass spectra (HRMS) were recorded using positive chemical ionisation (CI+) and ion impact (EI+) on a Joel MStation JMS-700 instrument or using positive ion electrospray (ESI+) technique on a Bruker micrOTOF-Q instrument by technical staff at the University of Glasgow.

Melting points were recorded using a Barnstead Electrothermal 9100 melting point apparatus. Where no solvent is indicated, the solid obtained from the described procedure was melted directly without recrystallisation. Optical rotations ($[\alpha]_D$) were determined using a Rudolph Research Analytical Autopol IV or V digital polarimeter. Elemental analyses were performed using an Exeter Analytical Elemental Analyser EA 440. X-ray crystallography was performed at the University of Glasgow by Dr Claire Wilson.

Experimental Procedures

***tert*-Butyldimethyl{[(2*R*,3*S*,6*R*)-6-(prop-1-en-2-yl)-2-(prop-2-yn-1-yl)oxan-3-yl]oxy}silane (24).**

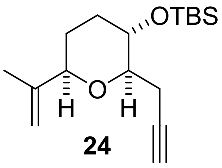

24

To a stirred solution of ester **21** (1.75 g, 5.11 mmol) in anhydrous dichloromethane (100 mL) at −78 °C was added *i*-Bu_2AlH (5.50 mL of a 1.0 M solution in dichloromethane, 5.50 mmol) dropwise over 5 minutes. The resulting solution was left to stir for 1 hour before the reaction was quenched by the addition of water (15 mL) and warmed to 0 °C for 15 minutes. A saturated aqueous solution of potassium sodium tartrate (30 mL) was added and the resulting solution was stirred vigorously for 1 hour at room temperature (two clear phases were obtained). The phases were separated and the aqueous phase was extracted with ethyl acetate (3 × 30 mL). The combined organic extracts were washed with brine (50 mL), dried ($MgSO_4$) and concentrated under reduced pressure to give the crude aldehyde, which was used in the next step without further purification.

To a stirred solution of aldehyde and potassium carbonate (1.41 g, 10.2 mmol) in anhydrous methanol (100 mL) at room temperature was added Bestmann-Ohira reagent (1.30 mL, 8.66 mmol) dropwise over 5 minutes. The resulting solution was stirred for 16 hours before the reaction was quenched by the addition of a saturated aqueous solution of sodium bicarbonate (40 mL). The resulting mixture was diluted with water (80 mL) and ethyl acetate (200 mL), and the phases were separated. The aqueous phase was extracted with ethyl acetate (3 × 80 mL) and the combined organic extracts were washed with brine (150 mL), dried ($MgSO_4$) and then concentrated under reduced pressure. The residue was purified by flash column chromatography on silica gel (petroleum ether-ethyl acetate, 100:1) to afford the alkyne **24** (1.29 g, 86% over two steps) as a colourless oil. R_f = 0.83 (petroleum ether-ethyl acetate, 20:1); $[\alpha]_D^{23}$ +45.5 (*c* = 0.390, $CHCl_3$); ν_{max} 3314, 2949, 2930, 2885, 2857, 2122, 1653, 885, 835, 775, 669, 635 cm^{-1}; ^1H NMR (400 MHz, $CDCl_3$) δ 5.00 (1H, br s), 4.82 (1H, br s), 3.75 (1H, br d, *J* = 8.9 Hz), 3.54−3.46 (1H, m), 3.27 (1H, ddd, *J* = 8.9, 6.0, 3.4 Hz), 2.63 (1H, ddd, *J* = 16.9, 3.4, 2.8 Hz), 2.47 (1H, ddd, *J* = 16.9, 6.0, 2.7 Hz), 2.09–1.98 (1H, m), 1.95 (1H, dd, *J* = 2.8, 2.7 Hz), 1.82–1.76 (1H, m), 1.75 (3H, s), 1.56–1.49 (2H, m), 0.89 (9H, s), 0.10 (3H, s), 0.08 (3H, s); ^{13}C NMR (101 MHz, $CDCl_3$) δ 145.6, 110.7, 81.6, 80.4, 80.2, 69.8, 69.4, 33.4, 29.9, 25.9, 22.4, 19.1, 18.1, −3.9, −4.6; HRMS (ESI+) $[M+Na]^+$ calcd. for $C_{17}H_{30}O_2SiNa$

317.1898, found 317.1907 (Δ +3.0 ppm). Anal. calcd for $C_{17}H_{30}O_2Si$: C, 69.33%; H, 10.27%. Found: C, 68.93%, H, 10.46%.

5-{[(2*R*,3*S*,6*R*)-3-(*tert*-Butyldimethylsilyloxy)-6-(prop-1-en-2-yl)oxan-2-yl]methyl}-1-[(4-methylphenyl)sulfonyl]-1*H*-1,2,3-triazole (25).

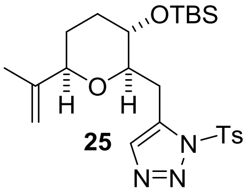

25

To a stirred solution of alkyne **24** (0.22 g, 0.75 mmol) in anhydrous THF (3.8 mL) at −78 °C was added *n*-butyl lithium (0.36 mL of a 2.5 M solution in dichloromethane, 0.90 mmol) dropwise over 5 minutes. The reaction mixture was stirred for 40 minutes before addition of tosyl azide (0.12 mL, 0.78 mmol) in one portion. The reaction mixture was stirred for 1 hour before the reaction was quenched by the addition of a saturated aqueous solution of ammonium chloride (3 mL), diluted with ethyl acetate (5 mL) and warmed to room temperature. The phases were separated and the aqueous phase was extracted with ethyl acetate (3 × 5 mL). The combined organic extracts were washed with brine (10 mL), dried ($MgSO_4$) and concentrated under reduced pressure. The residue was purified by flash column chromatography on silica gel (petroleum ether-ethyl acetate, gradient elution 20:1 → 10:1) to give the triazole **25** (0.32 g, 87%) as a colourless oil. R_f = 0.46 (petroleum ether-ethyl acetate, 9:1); $[\alpha]_D^{20}$ −16.6 (c = 0.375, $CHCl_3$); ν_{max} 2949, 2928, 2857, 1653, 1595, 1545, 893, 835, 775, 667, 583, 542 cm^{-1}; ^1H NMR (400 MHz, $CDCl_3$) δ 7.97−7.93 (2H, m), 7.55 (1H, s), 7.37−7.31 (2H, m), 4.89 (1H, br s), 4.78 (1H, s), 3.71–3.65 (2H, m), 3.47−3.38 (2H, m), 2.94−2.86 (1H, m), 2.44 (3H, s), 2.14–2.07 (1H, m), 1.86–1.80 (1H, m), 1.67 (3H, s), 1.60–1.52 (2H, m), 0.94 (9H, s), 0.15 (3H, s), 0.12 (3H, s); ^{13}C NMR (101 MHz, $CDCl_3$) δ 146.9, 144.8, 137.8, 134.3, 134.0, 130.4, 128.7, 110.9, 80.5, 80.4, 71.2, 33.6, 29.7, 27.4, 26.0, 21.9, 19.4, 18.1, −3.9, −4.5; HRMS (ESI+) $[M+Na]^+$ calcd. for $C_{24}H_{37}N_3O_4SSiNa$ 514.2166, found 514.2155 (Δ −2.1 ppm).

(1*R*,2*S*,5*Z*,8*R*)-2-(*tert*-Butyldimethylsilyloxy)-6-methyl-11-oxabicyclo[6.2.1]undec-5-en-9-one (23).

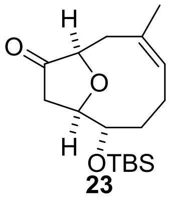

23

To a solution of triazole **25** (18 mg, 37 μmol) in anhydrous DCE (1.4 mL) was added $Rh_2(OAc)_4$ (1.6 mg, 3.6 μmol) in one portion. The resulting solution was stirred at reflux temperature in a sealed tube for 2 hours. The solution was cooled to room temperature and basic alumina (Brockmann III, 0.37 g) was added in one portion and the mixture stirred for 30 minutes. The mixture was filtered through Celite and the filter cake was washed with DCM (5 mL). The filtrate was concentrated under reduced pressure and the residue was purified by flash column chromatography on silica gel (petroleum ether-ethyl acetate, gradient elution 50:1 → 25:1) to give the *Z*-alkene **23** (5.5 mg, 48%) as a colourless solid.

The NMR (^1H and ^{13}C), IR and mass spectrometry data were consistent with those reported for compound **23** previously.[1]

3-[(2*R*,3*S*,6*R*)-3-(*tert*-Butyldimethylsilyloxy)-6-(prop-1-en-2-yl)oxan-2-yl]-2-oxopropyl 2-chloroacetate (26).

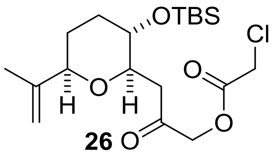

26

To a solution of alkyne **24** (0.50 g, 1.7 mmol) in anhydrous DCE (1.7 mL) was added $SPhosAuNTf_2$ (7.5 mg, 8.5 μmol), chloroacetic acid (32 mg, 0.34 mmol) and 5-bromo-2-methylpyridine-*N*-oxide (38 mg, 0.20 mmol) in one portion. The resulting solution was stirred at room temperature in a sealed vial for 16 hours and then concentrated under reduced pressure. The residue was purified by flash column chromatography on silica gel (petroleum ether-ethyl acetate, gradient elution 40:1 → 20:1) to deliver the ester **26** (25 mg, 36%) as a colourless solid. R_f = 0.45 (petroleum ether-ethyl acetate, 5:1);); $[\alpha]_D^{23}$ +86.4 (*c* = 0.500, $CHCl_3$); ν_{max} 2953, 2930, 2895, 2857, 1761, 1742, 1655, 901, 837, 777 cm^{-1}; ^1H NMR (500 MHz, $CDCl_3$) δ 5.24 (1H, dd, *J* = 7.5, 5.6 Hz), 4.99 (1H, br s), 4.96−4.93 (1H, m), 4.21 (1H, ddd, *J* = 8.9, 6.5, 3.3 Hz), 4.05 (2H, s), 4.03 (1H, d, *J* = 17.0 Hz), 3.94 (1H, ddd, *J* = 6.5, 5.2, 3.3 Hz), 3.85 (1H, d, *J* = 17.0 Hz), 2.58 (1H, ddd, *J* = 18.0, 8.9, 1.1 Hz), 2.35 (1H, dd, *J* = 18.0, 6.5 Hz), 1.89−1.80 (1H, m), 1.73 (3H, s), 1.73−1.65 (1H, m), 1.51−1.35 (2H, m), 0.87 (9H, s), 0.08 (3H, s), 0.05 (3H, s); ^{13}C NMR (126 MHz, $CDCl_3$) δ 214.9, 166.7, 141.9, 114.1, 80.9, 79.2, 72.8, 71.4, 41.1, 37.3, 30.0, 28.2, 26.0, 18.3, 18.1, −4.2, −4.4; HRMS (ESI+) $[M+Na]^+$ calcd. for $C_{19}H_{33}O_5SiClNa$ 427.1678, found 427.1672 (Δ −1.3 ppm).

Methyl 2-[(1*R*,2*S*,3*S*,8*R*,10*Z*,14*S*)-14-(*tert*-butyldimethylsilyloxy)-6-ethoxy-10-methyl-15-oxatricyclo[6.6.1.0^{2,7}]pentadeca-6,10-dien-3-yl]prop-2-enoate (29).

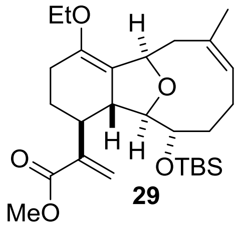

29

To a stirred solution of ketone **27**[1] (600 mg, 1.38 mmol) and *N*-phenyl-bis(trifluoromethane-sulfonimide) (1.23 g, 3.44 mmol) in anhydrous THF (28 mL) at −78 °C was added NaHMDS (1.4 mL of a 2.0 M solution in THF, 2.8 mmol) dropwise over 10 minutes. The resulting solution was stirred for 2.5 hours and then the reaction was quenched by the addition of water (15 mL). The mixture was diluted with diethyl ether (20 mL) and then allowed to warm to room temperature. The phases were separated and the aqueous phase was extracted with diethyl ether (3 × 10 mL). The combined organic extracts were washed with brine (20 mL), dried ($MgSO_4$) and concentrated under reduced pressure to give the crude enol triflate **28**, which was used in the next step without purification.

To a stirred solution of enol triflate **28**, *N*,*N*-diisopropylethylamine (0.96 mL, 5.5 mmol) in anhydrous methanol (69 mL) was added palladium tetrakis(triphenylphosphine) (159 mg, 0.138 mmol) and sparged with carbon monoxide for 15 minutes. The resulting solution was stirred under an atmosphere of carbon monoxide at room temperature for 16 hours. The reaction was terminated by sparging the reaction mixture with argon for 30 minutes. The mixture was concentrated under reduced pressure and the residue was purified by flash column chromatography on silica gel (petroleum ether-diethyl ether, 20:1) to give the ester **29** (633 mg, 96%) as a colourless oil. R_f = 0.80 (petroleum ether-ethyl acetate, 3:1); $[\alpha]_D^{24}$ +142 (*c* = 0.353, $CHCl_3$); ν_{max} 2955, 2930, 2895, 2857, 1721, 941, 833, 773 cm^{-1}; ^1H NMR (400 MHz, $CDCl_3$) δ 6.19 (1H, d, *J* = 1.2 Hz), 5.56 (1H, d, *J* = 1.2 Hz), 5.29 (1H, dd, *J* = 11.2, 5.5 Hz), 4.89−4.85 (1H, m), 3.80 (1H, q, *J* = 7.0 Hz), 3.79 (1H, q, *J* = 7.0 Hz), 3.75 (3H, s), 3.76−3.71 (1H, m), 3.58 (1H, ddd, *J* = 3.6, 2.8, 2.8 Hz), 3.14 (1H, app td, *J* = 11.9, 8.9 Hz), 2.86–2.75 (2H, m), 2.35–2.27 (1H, m), 2.27–2.21 (2H, m), 1.94 (1H, dd, *J* = 14.0, 4.0 Hz), 1.90–1.72 (4H, m), 1.69 (3H, s), 1.52 (1H, ddd, *J* = 14.3, 8.9, 3.6 Hz), 1.23 (3H, t, *J* = 7.0 Hz), 0.86 (9H, s), 0.01 (3H, s), −0.01 (3H, s); ^{13}C NMR (101 MHz, $CDCl_3$) δ 167.2, 144.3, 143.6, 131.1, 129.8, 125.5, 120.1, 88.0, 75.4, 72.0, 63.0, 52.0, 46.5, 42.1, 37.6, 33.1, 30.9, 28.5, 26.3, 25.1, 22.0, 18.6, 15.9, −4.4, −4.5; HRMS (ESI+) $[M+Na]^+$ calcd. for $C_{27}H_{44}O_5SiNa$ 499.2850, found 499.2832 (Δ −3.6 ppm). Anal. calcd for $C_{27}H_{44}O_5Si$: C, 68.03%; H, 9.30%. Found: C, 68.27%, H, 9.30%.

(1*R*,6*S*,7*R*,8*R*,9*S*,12*Z*)-3-Ethoxy-6-(3-hydroxyprop-1-en-2-yl)-13-methyl-15-oxatricyclo-[6.6.1.0^{2,7}]pentadeca-2,12-dien-9-ol (30).

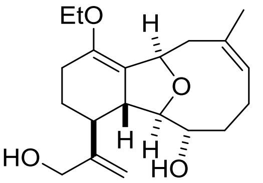

30

To a stirred solution of ester **29** (250 mg, 0.524 mmol) in anhydrous dichloromethane (8.6 mL) at −30 °C was added *i*-Bu_2AlH (1.6 mL of a 1.0 M solution in dichloromethane, 1.6 mmol) dropwise over 15 minutes. The resulting solution was stirred for 1.5 hours and then the reaction was quenched by the addition of water (2 mL) and the mixture was warmed to 0 °C for 15 minutes. A saturated aqueous solution of potassium sodium tartrate (10 mL) was added and the resulting mixture was stirred vigorously for 1 hour at room temperature (two clear phases were obtained). The phases were separated and the aqueous phase extracted with diethyl ether (3 × 10 mL). The combined organic extracts were washed with brine (20 mL), dried ($MgSO_4$) and concentrated under reduced pressure. The residue was purified by flash column chromatography on silica gel (petroleum ether-ethyl acetate, gradient elution 2:1 → 1:2) to provide the diol **30** (140 mg, 80%) as a colourless solid. R_f = 0.21 (petroleum ether-ethyl acetate, 1:2); m.p. 108−110 °C; $[\alpha]_D^{23}$ +150 (*c* = 1.40, $CHCl_3$); ν_{max} 3366, 2965, 2922, 2857, 1713, 1643, 991, 895, 731 cm^{-1}; ^{1}H NMR (400 MHz, C_6D_6) δ 5.39 (1H, dd, *J* = 11.2, 5.7 Hz), 5.13−5.08 (1H, m), 5.02 (1H, d, *J* = 1.1 Hz), 4.77 (1H, d, *J* = 1.1 Hz), 3.99 (1H, dd, *J* = 9.0, 3.2 Hz), 3.86−3.71 (3H, m), 3.45 (1H, dq, *J* = 9.5, 7.0 Hz), 3.40 (1H, dq, *J* = 9.5, 7.0 Hz), 3.07−2.88 (2H, m), 2.87 (1H, br d, *J* = 13.8 Hz), 2.77−2.68 (1H, m), 2.19−2.08 (1H, m), 2.11 (1H, dd, *J* = 13.8, 4.0 Hz), 1.92−1.81 (3H, m), 1.80 (3H, s), 1.73−1.55 (3H, m), 1.50−1.30 (2H, m), 1.02 (3H, t, *J* = 7.0 Hz); ^{13}C NMR (101 MHz, C_6D_6) δ 152.0, 144.7, 131.5, 129.6, 120.0, 109.6, 87.9, 75.6, 72.2, 65.0, 62.5, 47.3, 43.1, 37.9, 33.0, 30.8, 28.4, 25.0, 22.4, 15.8; HRMS (ESI+) $[M+Na]^+$ calcd. for $C_{20}H_{30}O_4Na$ 357.2036, found 357.2031 (Δ −1.4 ppm).

2-[(1*R*,2*S*,3*S*,8*R*,10*Z*,14*S*)-6-Ethoxy-14-hydroxy-10-methyl-15-oxatricyclo[6.6.1.0^{2,7}]-pentadeca-6,10-dien-3-yl]prop-2-en-1-yl acetate (S1).

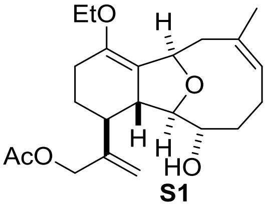

S1

To a stirred solution of diol **30** (250 mg, 0.747 mmol), DMAP (91 mg, 0.74 mmol) and distilled triethylamine (0.21 mL, 1.5 mmol) in anhydrous dichloromethane (30 mL) at 0 °C was added distilled acetic anhydride (4.0 mL of a 0.2 M solution in dichloromethane, 0.80 mmol). The resulting solution was warmed to room temperature and allowed to stir for 45 minutes. The mixture then cooled to 0 °C and the reaction was quenched by the addition of a saturated aqueous solution of ammonium chloride (20 mL) and diluted with diethyl ether (60 mL). The phases were separated and the aqueous phase was extracted with diethyl ether (3 × 20 mL). The combined organic extracts were washed with brine (40 mL), dried ($MgSO_4$) and concentrated under reduced pressure. The residue was purified by flash column chromatography on silica gel (petroleum ether-ethyl acetate, 10:1) to deliver the acetate (274 mg, 97%) as a colourless oil. R_f = 0.62 (petroleum ether-ethyl acetate, 1:2); $[\alpha]_D^{23}$ +142 (*c* = 1.21, $CHCl_3$); ν_{max} 3439, 2974, 2928, 2882, 2857, 1742, 1709, 1670, 955, 907, 777 cm^{-1}; ^1H NMR (400 MHz, $CDCl_3$) δ 5.34 (1H, dd, *J* = 11.2, 5.8 Hz), 5.08 (1H, d, *J* = 1.3 Hz), 4.98 (1H, d, *J* = 1.3 Hz), 4.87–4.83 (1H, m), 4.48 (2H, br s), 3.84−3.77 (1H, m), 3.80 (1H, q, *J* = 7.0 Hz), 3.80 (1H, q, *J* = 7.0 Hz), 3.69–3.64 (1H, m), 2.86–2.77 (1H, m), 2.75 (1H, br d, *J* = 14.0), 2.72–2.64 (2H, m), 2.35–2.15 (2H, m), 2.10 (3H, s), 2.03–1.95 (1H, m), 1.97 (1H, dd, *J* = 14.0, 4.0 Hz), 1.94–1.86 (1H, m), 1.86–1.74 (2H, m), 1.69 (3H, s), 1.67–1.55 (2H, m), 1.24 (3H, d, *J* = 7.0); ^{13}C NMR (101 MHz, $CDCl_3$) δ 170.8, 146.1, 144.8, 131.6, 129.2, 119.5, 113.4, 87.4, 75.3, 71.5, 65.8, 62.9, 46.2, 43.8, 37.5, 32.4, 30.2, 28.3, 25.0, 21.6, 21.1, 15.9; HRMS (ESI+) $[M+Na]^+$ calcd. for $C_{22}H_{32}O_5Na$ 399.2142, found 399.2130 (Δ −2.9 ppm).

2-[(1*R*,2*S*,3*S*,8*R*,10*Z*)-6-Ethoxy-10-methyl-15-oxatricyclo[6.6.1.0^{2,7}]pentadeca-6,10-dien-14-one-6-yl]prop-2-en-1-yl acetate (31).

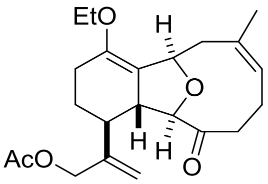

31

To a stirred solution of acetate **S1** (270 mg, 0.717 mmol) in anhydrous dichloromethane (11 mL) at room temperature was added pyridine (0.23 mL, 2.9 mmol) and Dess-Martin periodinane (551 mg, 1.30 mmol). The resulting mixture was stirred for 2 hours then diluted with diethyl ether (30 mL). The reaction was quenched by the addition of a saturated aqueous solution of sodium thiosulfate (10 mL). The biphasic mixture was stirred for 5 minutes and then a saturated aqueous solution of sodium bicarbonate (5 mL) was added. The resulting mixture was stirred vigorously for 30 minutes. The phases were separated and the aqueous phase was extracted with diethyl ether (3 × 15 mL). The combined organic extracts were washed

with brine (30 mL), dried ($MgSO_4$) and concentrated under reduced pressure. The residue was purified by flash column chromatography on silica gel (petroleum ether-ethyl acetate, 4:1) to provide the ketone **31** (223 mg, 83%) as a colourless oil. R_f = 0.77 (petroleum ether-ethyl acetate, 1:1); $[\alpha]_D^{31}$ +108 (*c* = 0.980, $CHCl_3$); ν_{max} 2974, 2930, 2868, 1742, 1705, 1647, 901, 845 cm^{-1}; ^1H NMR (400 MHz, $CDCl_3$) δ 5.45 (1H, br s), 5.10 (1H, br s), 5.06 (1H, d, *J* = 1.4 Hz), 5.00 (1H, d, *J* = 1.4 Hz), 4.70 (1H, d, *J* = 14.0 Hz), 4.66 (1H, d, *J* = 14.0 Hz), 3.97 (1H, d, *J* = 6.0 Hz), 3.83 (2H, q, *J* = 7.0 Hz), 2.98 (1H, ddd, *J* = 13.0, 6.0, 3.9 Hz), 2.81 (1H, br s), 2.44–2.31 (2H, m), 2.28–2.16 (1H, m), 2.10 (3H, s), 2.09–1.89 (5H, m), 1.74 (3H, s), 1.79–1.66 (2H, m), 1.24 (3H, d, *J* = 7.0 Hz). ^{13}C NMR (101 MHz, $CDCl_3$) δ 171.0, 146.0, 145.0, 134.8, 127.0, 119.5, 113.4, 88.7, 80.3, 64.9, 63.3, 46.7, 41.5, 37.1, 29.3, 29.3, 24.9, 24.1, 21.2, 15.7 (peaks for C-9 and C-8 were not observed); HRMS (ESI+) $[M+Na]^+$ calcd. for $C_{22}H_{30}O_5Na$ 397.1985, found 397.1979 (Δ −1.7 ppm). Anal. calcd for $C_{22}H_{30}O_5$: C, 70.56%; H, 8.08%. Found: C, 70.25%, H, 7.89%.

2-[(1*R*,2*S*,3*S*,8*R*,10*Z*)-6-Ethoxy-10-methyl-15-oxatricyclo[6.6.1.0^{2,7}]pentadeca-6,10-dien-14-one-3-yl]prop-2-en-1-yl acetate (31).

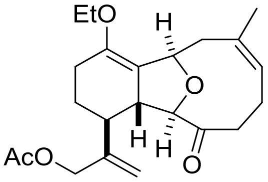

31

To a stirred solution of diol **30** (820 mg, 2.45 mmol), DMAP (299 mg, 2.45 mmol) and distilled triethylamine (0.68 mL, 4.9 mmol) in anhydrous dichloromethane (98 mL) at 0 °C was added distilled acetic anhydride (13.5 mL of a 0.2 M solution in dichloromethane, 2.70 mmol). The resulting solution was warmed to room temperature and stirred for 45 minutes. The mixture was cooled to 0 °C and the reaction was quenched by the addition of a saturated aqueous solution of ammonium chloride (75 mL) and the mixture was diluted with diethyl ether (200 mL). The phases were separated and the aqueous phase was extracted with diethyl ether (3 × 40 mL). The organic extracts were combined and washed with brine (75 mL), dried ($MgSO_4$) and concentrated under reduced pressure to give crude acetate **S1**, which was used in the next step without purification.

To a stirred solution of acetate **S1** in anhydrous dichloromethane (38 mL) at room temperature was added pyridine (0.79 mL, 9.8 mmol) and Dess-Martin periodinane (1.87 g, 4.41 mmol) in one portion. The mixture was stirred for 2 hours then diluted with diethyl ether (80 mL). The reaction was quenched by the addition of a saturated aqueous solution of sodium thiosulfate (40 mL). The mixture was stirred for 5 minutes and a saturated aqueous solution of sodium

bicarbonate (10 mL) was then added. The resulting mixture was stirred vigorously for 30 minutes. The phases were separated and the aqueous phase was extracted with diethyl ether (3 × 20 mL). The combined organic extracts were washed with brine (50 mL), dried ($MgSO_4$) and concentrated under reduced pressure. The residue was purified by flash column chromatography on silica gel (petroleum ether-ethyl acetate, 4:1) to give the ketone **31** (905 mg, 98%) as a colourless oil.

(1*R*,2*R*,6*S*,7*S*,8*R*,9*R*,12*Z*)-9-Hydroxy-6-(3-hydroxyprop-1-en-2-yl)-9,13-dimethyl-15-oxa-tricyclo[6.6.1.0^{2,7}]pentadec-12-en-3-one (32).

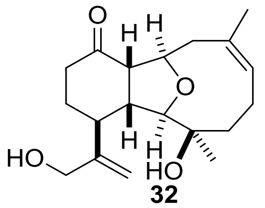

32

To a stirred solution of MeMgCl (4.60 mL of a 3.0 M solution in THF, 13.8 mmol) in anhydrous THF (32 mL) at 0 °C was added a solution of ketone **31** (520 mg, 1.39 mmol) in anhydrous THF (64 mL) dropwise over 5 minutes. The resulting solution was stirred for 30 minutes and then allowed to warm to room temperature for 1.5 hours. The reaction was quenched by slow transfer of the reaction mixture into a round bottom flask containing an aqueous solution of 1 M HCl (60 mL) over a period 30 minutes at room temperature. The resulting solution was stirred for 2 hours and then diluted by the addition of a saturated aqueous solution of ammonium chloride (60 mL), water (60 mL) and ethyl acetate (120 mL). The phases were separated and the aqueous phase was extracted with ethyl acetate (3 × 40 mL). The combined organic extracts were washed with brine (60 mL), dried ($MgSO_4$) and concentrated under reduced pressure. The residue was purified by flash column chromatography on silica gel (petroleum ether-ethyl acetate, gradient elution 1:2 → 1:5) to deliver the diol **32** (388 mg, 87%) as a colourless solid. R_f = 0.22 (petroleum ether-ethyl acetate, 1:2); m.p. 118−120 °C; $[\alpha]_D^{24}$ +29.3 (*c* = 0.420, $CHCl_3$); ν_{max} 3410, 2963, 2926, 2853, 1703, 1530, 977, 899, 735, 703 cm^{-1}; ^1H NMR (400 MHz, $CDCl_3$) δ 5.79 (1H, dd, *J* = 11.3, 6.0 Hz), 5.25 (1H, d, *J* = 0.8 Hz), 5.06 (1H, d, *J* = 0.8 Hz), 4.41 (1H, ddd, *J* = 8.9, 3.2, 3.2 Hz), 4.16 (1H, d, *J* = 0.8 Hz), 4.14 (1H, d, *J* = 0.8 Hz), 3.86 (1H, br d, *J* = 1.3 Hz), 3.06–2.85 (5H, m), 2.49–2.43 (2H, m), 2.38 (1H, ddd, *J* = 11.7, 11.7, 3.0 Hz), 2.09–1.94 (3H, m), 1.91 (3H, s), 1.87–1.72 (3H, m), 1.70 (1H, ddd, *J* = 14.6, 10.7, 6.0 Hz), 1.00 (3H, s); ^{13}C NMR (101 MHz, $CDCl_3$) δ 210.0, 150.7, 135.9, 128.8, 112.2, 91.5, 79.6, 74.9, 65.3, 54.0, 48.7, 41.2, 38.8, 38.6, 35.6, 31.1, 28.9, 28.5, 26.6; HRMS (ESI+) $[M+Na]^+$ calcd. for $C_{19}H_{28}O_4Na$ 343.1880, found 343.1870 (Δ −2.7 ppm).

(1*R*,2*R*,3*S*,7*S*,11*R*,14*Z*,17*R*)-11,15-Dimethyl-8-methylidene-10,18-dioxatetracyclo-[9.7.0.0^{2,7}.0^{3,17}]octadec-14-en-4-one (33).

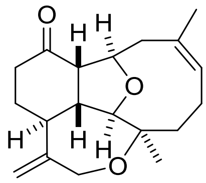

33

To a stirred solution of diol **32** (645 mg, 2.01 mmol) in anhydrous chloroform (101 mL) at 0 °C was added 2,6-lutidine (1.20 mL, 10.4 mmol) followed by trifluoromethanesulfonic anhydride (16.1 mL of a 0.5 M solution in dichloromethane, 8.05 mmol) dropwise over 5 minutes. The mixture was stirred for 1 hour and then the reaction was quenched by the addition of a saturated aqueous solution of ammonium chloride (60 mL). The mixture was diluted with ethyl acetate (150 mL) and the phases were separated. The aqueous phase was extracted with ethyl acetate (3 × 20 mL) and the organic extracts were combined and then washed successively with an aqueous solution of 1 M HCl (2 × 50 mL), brine (75 mL), dried ($MgSO_4$) and concentrated under reduced pressure. The residue was purified by flash column chromatography on silica gel (petroleum ether-ethyl acetate, 10:1) to deliver the tetracyclic ketone **33** (591 mg, 97%) as a colourless solid. R_f = 0.47 (petroleum ether-ethyl acetate, 4:1); m.p. 137−140 °C; $[\alpha]_D^{23}$ −26.6 (*c* = 0.630, $CHCl_3$); ν_{max} 2980, 2963, 2936, 2917, 2869, 2850, 1708, 1639, 917, 895, 852, 730 cm^{-1}; ^{1}H NMR (400 MHz, C_6D_6) δ 5.39 (1H, dd, *J* = 10.5, 6.8 Hz), 5.26 (1H, ddd, *J* = 4.3, 2.9, 2.9 Hz), 4.77 (1H, br s), 4.56 (1H, br s), 4.05 (1H, d, *J* = 14.2 Hz), 3.86 (1H, d, *J* = 14.2 Hz), 3.62 (1H, d, *J* = 9.3 Hz), 2.86−2.75 (1H, m), 2.66−2.61 (1H, m), 2.64 (1H, ddd, *J* = 12.0, 11.0, 9.3 Hz), 2.44 (1H, dd, *J* = 12.0, 2.9 Hz), 2.02−1.97 (2H, m), 1.88 (1H, dd, *J* = 14.2, 8.7 Hz), 1.86−1.80 (1H, m), 1.74 (1H, dd, *J* = 14.2, 10.7 Hz), 1.64 (1H, dd, *J* = 14.5, 4.3 Hz), 1.57 (3H, s), 1.54−1.47 (1H, m), 1.45 (3H, d, *J* = 0.7 Hz), 1.44−1.39 (1H, m), 1.37−1.26 (1H, m); ^{13}C NMR (101 MHz, C_6D_6) δ 208.4, 149.9, 131.8, 129.2, 110.7, 92.4, 76.9, 76.6, 67.4, 53.0, 47.8, 43.3, 38.5, 37.2, 36.6, 29.3, 27.2, 25.5, 23.4; HRMS (ESI+) $[M+Na]^+$ calcd. for $C_{19}H_{26}O_3Na$ 325.1774, found 325.1768 (Δ −1.9 ppm).

(1*R*,2*R*,3*S*,7*R*,8*S*,11*R*,14*Z*,17*R*)-8,11,15-Trimethyl-10,18-dioxatetracyclo[9.7.0.0^{2,7}.0^{3,17}]-octadec-14-en-4-one (34).

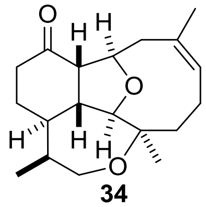

34

To a stirred solution of tetracyclic ketone **33** (590 mg, 1.95 mmol) in ethyl acetate (49 mL) was added PtO_2 (44 mg, 0.19 mmol) and the resulting suspension stirred under an atmosphere of hydrogen for 1.5 hours. The suspension was filtered over celite, washed with ethyl acetate and concentrated under reduced pressure. The residue was purified by flash column chromatography on silica gel (petroleum ether-ethyl acetate, 10:1) to afford the ketone **34** (536 mg, 90%) as a colourless solid. R_f = 0.41 (petroleum ether-ethyl acetate, 3:1); m.p. 113−115 °C; $[\alpha]_D^{23}$ −49.7 (*c* = 0.320, $CHCl_3$); ν_{max} 2961, 2924, 2874, 1713, 851, 750 cm^{-1}; ^1H NMR (400 MHz, $CDCl_3$) δ 5.56−5.49 (1H, m,), 4.94 (1H, ddd, *J* = 4.4, 3.0, 3.0 Hz), 3.80 (1H, dd, *J* = 13.4, 0.6 Hz), 3.76 (1H, d, *J* = 9.0 Hz), 3.46 (1H, dd, *J* = 13.4, 3.3 Hz), 2.85 (1H, ddd, *J* = 11.6, 11.6, 9.0 Hz), 2.69–2.56 (3H, m), 2.50 (1H, dd, *J* = 18.6, 6.9 Hz), 2.31 (1H, ddd, *J* = 18.6, 11.1, 7.9 Hz), 1.95–1.78 (4H, m), 1.76 (3H, s), 1.76–1.66 (3H, m), 1.37–1.24 (1H, m), 1.33 (3H, s), 1.02 (3H, d, *J* = 7.1 Hz); ^{13}C NMR (101 MHz, $CDCl_3$) δ 211.3, 131.7, 128.9, 90.8, 77.1, 76.3, 67.2, 53.2, 44.5, 43.8, 39.4, 39.3, 37.3, 37.0, 29.4, 26.3, 26.2, 23.2, 11.3; HRMS (ESI+) $[M+Na]^+$ calcd. for $C_{19}H_{28}O_3Na$ 327.1931, found 327.1921 (Δ −2.9 ppm).

(1*R*,2*R*,3*S*,7*R*,8*S*,11*R*,14*Z*,17*R*)-8,11,15-Trimethyl-5-methylidene-10,18-dioxatetracyclo-[9.7.0.0^{2,7}.0^{3,17}]octadec-14-en-4-one (36).

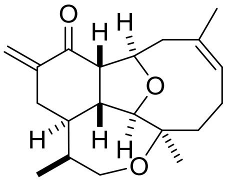

36

To a solution of ketone **34** (64 mg, 0.20 mmol) in anhydrous DMF (2.1 mL) in a sealed tube was added Bredereck's reagent [*tert*-butoxy bis(dimethylamino)methane] (0.14 mL, 0.68 mmol) in one portion and the resulting solution was stirred at 100 °C for 2 hours. The reaction mixture was cooled to room temperature and then diluted with ethyl acetate (15 mL) and water (5 mL). The phases were separated and the organic phase washed successively with water (3 × 5 mL), a 10 mol% aqueous solution of lithium chloride (5 mL) and brine (5 mL). The organic phase was then dried ($MgSO_4$) and concentrated under reduced pressure to give a residue that was purified by flash column chromatography on silica gel (petroleum ether-ethyl acetate, gradient elution 2:1 → 1:5) to give the enaminone **35** as a pale yellow solid. The enaminone **35** was used directly in the next step without further purification.

To a stirred solution of the enaminone **35** in anhydrous dichloromethane (2.6 mL) at −78 °C was added *i*-Bu_2AlH (0.30 mL of a 1.0 M solution in dichloromethane, 0.30 mmol) dropwise and the mixture was stirred for 20 minutes. The mixture was warmed to room temperature and stirred for a further 1 hour. A solution of iodomethane (0.13 mL, 2.1 mmol) in anhydrous

dichloromethane (4.2 mL) was added and the resulting mixture was stirred for 1 hour. The reaction mixture was cooled to 0 °C and a saturated aqueous solution of potassium sodium tartrate (8 mL) was added followed by diethyl ether (10 mL). The resulting mixture was warmed to room temperature and stirred vigorously for 1 hour (two clear phases were obtained). The phases were separated and the aqueous phase extracted with diethyl ether (3 × 5 mL). The combined organic extracts were washed with brine (10 mL), dried ($MgSO_4$) and concentrated under reduced pressure. The residue was purified by flash column chromatography on silica gel (petroleum ether-ethyl acetate, 10:1) to afford the enone **36** (34.5 mg, 52%) as a colourless solid. R_f = 0.60 (petroleum ether-ethyl acetate, 3:1); m.p. 118−120 °C; $[\alpha]_D^{25}$ +1.99 (*c* = 0.795, $CHCl_3$); ν_{max} 2963, 2924, 2874, 2851, 1699, 1624, 939, 923, 850, 824, 812 cm^{-1}; ^1H NMR (400 MHz, $CDCl_3$) δ 6.06 (1H, dd, *J* = 1.1, 1.1 Hz), 5.57−5.50 (1H, m), 5.23 (1H, br s), 5.01−4.96 (1H, m), 3.81 (1H, br d, *J* = 9.0 Hz), 3.81 (1H, dd, *J* = 13.4, 1.5 Hz), 3.46 (1H, dd, *J* = 13.4, 3.2 Hz), 2.84 (1H, ddd, *J* = 11.9, 11.5, 9.0 Hz), 2.75−2.52 (4H, m), 2.40 (1H, dd, *J* = 14.9, 2.2 Hz), 1.91−1.76 (3H, m), 1.91 (1H, dd, *J* = 14.6, 4.4 Hz), 1.75 (3H, s), 1.73−1.65 (1H, m), 1.52 (1H, dddd, *J* = 13.5, 11.5, 4.7, 2.2 Hz), 1.34 (3H, s), 1.04 (3H, d, *J* = 7.1 Hz); ^{13}C NMR (101 MHz, $CDCl_3$) δ 199.6, 143.9, 131.8, 129.0, 120.8, 91.1, 78.4, 76.2, 67.1, 52.4, 43.7, 43.3, 39.4, 37.2, 37.0, 34.8, 29.6, 26.0, 23.2, 11.3; HRMS (ESI+) $[M+Na]^+$ calcd. for $C_{20}H_{28}O_3Na$ 339.1931, found 339.1931 (Δ 0.0 ppm).

(1*R*,2*R*,3*R*,4*S*,7*R*,8*S*,11*R*,14*Z*,17*R*)-8,11,15-Trimethyl-5-methylidene-10,18-dioxatetra-cyclo[9.7.0.0^{2,7}.0^{3,17}]octadec-14-en-4-ol (37).

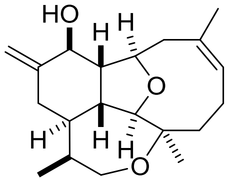

37

To a solution of ketone **34** (12.3 mg, 40.0 μmol) in anhydrous DMF (0.4 mL) in a sealed tube was added Bredereck's reagent [*tert*-butoxy bis(dimethylamino)methane] (27 μL, 0.13 mmol) in one portion. The resulting mixture was heated to 100 °C and stirred at this temperature for 2 hours. The mixture was cooled to room temperature and diluted with ethyl acetate (5 mL) and water (2 mL). The phases were separated and the organic phase was washed successively with water (3 × 2 mL), a 10 mol% aqueous solution of lithium chloride (3 mL) and brine (3 mL). The aqueous phase was then dried ($MgSO_4$) and concentrated under reduced pressure. The residue was purified by flash column chromatography on silica gel (petroleum ether-ethyl acetate, gradient elution 2:1 → 1:2) to give the enaminone **35** as a pale yellow solid, which was used immediately in the next step.

To a stirred solution of the enaminone **35** in anhydrous dichloromethane (0.5 mL) at −78 °C was added *i*-Bu_2AlH (0.05 mL of a 1.0 M solution in dichloromethane, 0.05 mmol) dropwise and the mixture was stirred at −78 °C for 20 minutes. The mixture was warmed to room temperature and was stirred for a further 1 hour. A solution of iodomethane (30 μL, 0.48 mmol) in anhydrous dichloromethane (2.4 mL) was added and the resulting mixture was stirred for 1 hour. The mixture was cooled to 0 °C and a saturated aqueous solution of potassium sodium tartrate (2 mL) was added followed by diethyl ether (3 mL). The resulting mixture was warmed to room temperature and stirred vigorously for 1 hour (two clear phases were obtained). The phases were separated and the aqueous phase extracted with diethyl ether (3 × 2 mL). The combined organic extracts were washed with brine (3 mL), dried ($MgSO_4$) and concentrated under reduced pressure to give the crude enone **36**, which was used in the next step without purification.

To a stirred solution of the enone **36** in methanol (0.8 mL) at room temperature was added cerium(III) chloride heptahydrate (30 mg, 81 μmol). The mixture was stirred for 15 minutes and then cooled to −78 °C and then sodium borohydride (3 mg, 0.08 mmol) was added in one portion. The mixture was stirred at −78 °C for 2.5 hours, warmed to room temperature and stirred at this temperature for a further 1.5 hours. The reaction was quenched by the addition of a 50% aqueous solution of ammonium chloride (1 mL) and the resulting mixture was diluted with ethyl acetate (3 mL). The phases were separated and the aqueous phase was extracted with ethyl acetate (3 × 2 mL). The combined organic extracts were washed with brine (3 mL), dried ($MgSO_4$) and concentrated under reduced pressure. The residue was purified by flash column chromatography on silica gel (petroleum ether-ethyl acetate, gradient elution 10:1 → 4:1) to give the allylic alcohol **37** (6.3 mg, 49% over 3 steps) as a colourless solid. R_f = 0.43 (petroleum ether-ethyl acetate, 2:1); m.p. 134−136 °C; $[\alpha]_D^{26}$ +59.5 (*c* = 0.305, $CHCl_3$); ν_{max} 3402, 2961, 2928, 2878, 2857, 1647, 905, 729, 650 cm^{-1}: ^1H NMR (400 MHz, $CDCl_3$) δ 5.53–5.46 (1H, m), 5.19 (1H, d, *J* = 2.1 Hz), 4.98 (1H, d, *J* = 2.1 Hz), 4.52 (1H, ddd, *J* = 5.0, 2.8, 2.5 Hz), 4.34–4.24 (1H, m), 3.96 (1H, d, *J* = 8.9 Hz), 3.82 (1H, d, *J* = 13.2 Hz), 3.46 (1H, dd, *J* = 13.2, 3.2 Hz), 2.67–2.57 (2H, m), 2.50 (1H, ddd, *J* = 10.8, 8.9, 8.9 Hz), 2.38–2.31 (2H, m), 1.94 (1H, dd, *J* = 14.4, 5.0 Hz), 1.91–1.79 (3H, m), 1.78 (3H, s), 1.76–1.61 (3H, m), 1.56 (1H, br s), 1.37 (3H, s), 0.94 (3H, d, *J* = 7.0 Hz); ^{13}C NMR (101 MHz, $CDCl_3$) δ 149.6, 131.0, 129.2, 107.9, 91.4, 82.9, 76.2, 71.1, 67.4, 48.6, 42.0, 39.5, 39.0, 38.1, 36.6, 34.1, 29.3, 26.0, 23.1, 10.8; HRMS (EI+) $[M]^+$ calcd. for $C_{20}H_{30}O_3$ 318.2195, found 318.2208 (Δ +4.1 ppm).

(1*R*,2*R*,3*S*,5*S*,7*R*,8*S*,11*R*,14*Z*,17*R*)-5,8,11,15-Tetramethyl-10,18-dioxatetracyclo-[9.7.0.0^{2,7}.0^{3,17}]octadec-14-en-4-one (38a) and (1*R*,2*R*,3*S*,5*R*,7*R*,8*S*,11*R*,14*Z*,17*R*)-5,8,11,15-Tetramethyl-10,18-dioxatetracyclo-[9.7.0.0^{2,7}.0^{3,17}]octadec-14-en-4-one (38b).

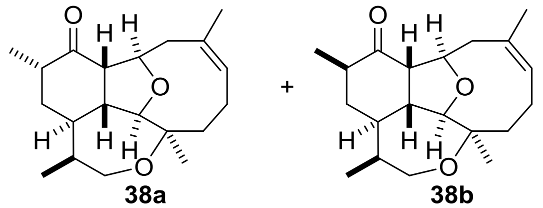

To a stirred solution of enone **36** (12 mg, 38 μmol) in anhydrous THF (0.8 mL) at −78 °C was added L-selectride (57 μL of a 1.0 M solution in THF, 57 μmol) dropwise. The resulting solution was stirred for 1 hour and then warmed to 0 °C for 30 minutes. The reaction was quenched by the addition of water (0.5 mL) and the mixture was warmed to room temperature and diluted with ethyl acetate (2 mL). The phases were separated and the aqueous phase was extracted with ethyl acetate (3 × 1 mL). The combined organic extracts were washed with brine (2 mL), dried ($MgSO_4$) and concentrated under reduced pressure. The residue was purified by flash column chromatography on silica gel (petroleum ether-ethyl acetate, 10:1) to give the diastereoisomeric ketones **38a** (3.6 mg, 30%) and **38b** (5.5 mg, 46%) as colourless solids. **38a**: R_f = 0.63 (petroleum ether-ethyl acetate, 4:1); m.p. 109–111 °C; $[\alpha]_D^{24}$ −12.1 (*c* = 0.162, $CHCl_3$); ν_{max} 2963, 2924, 2874, 1713, 922, 856, 806, 732 cm^{-1}; ^{1}H NMR (400 MHz, $CDCl_3$) δ 5.55–5.48 (1H, m), 4.98 (1H, ddd, *J* = 4.4, 2.9, 2.9 Hz), 3.79 (1H, d, *J* = 13.3 Hz), 3.71 (1H, d, *J* = 9.1 Hz), 3.44 (1H, dd, *J* = 13.3, 3.3 Hz), 2.87 (1H, ddd, *J* = 11.7, 9.2, 9.1 Hz), 2.70 (1H, dd, *J* = 11.7, 2.9 Hz), 2.69–2.56 (2H, m), 2.50 (1H, dq, *J* = 8.4, 7.1 Hz), 2.26 (1H, ddd, *J* = 13.3, 13.2, 8.4 Hz), 1.92–1.78 (3H, m), 1.77 (3H, s), 1.76–1.64 (2H, m), 1.40 (1H, dd, *J* = 13.3, 1.9 Hz), 1.33 (3H, s), 1.29–1.19 (1H, m), 1.12 (3H, d, *J* = 7.1 Hz), 1.00 (3H, d, *J* = 7.1 Hz); ^{13}C NMR (101 MHz, $CDCl_3$) δ 213.2, 131.6, 128.9, 90.3, 76.6, 76.2, 67.2, 52.9, 45.7, 41.6, 41.4, 39.5, 37.4, 37.0, 35.2, 29.4, 26.3, 23.2, 16.0, 11.3; HRMS (ESI+) $[M]^+$ calcd. for $C_{20}H_{30}O_3Na$ 341.2087, found 341.2078 (Δ −2.8 ppm). **38b**: R_f = 0.51 (petroleum ether-ethyl acetate, 4:1); m.p. 139−141 °C; $[\alpha]_D^{24}$ −61.1 (*c* = 0.195, $CHCl_3$); ν_{max} 2963, 2928, 2874, 1709, 914, 849, 806, 737 cm^{-1}; ^{1}H NMR (400 MHz, $CDCl_3$) δ 5.56–5.49 (1H, m), 4.92 (1H, ddd, *J* = 4.6, 3.0, 3.0 Hz), 3.78 (1H, dd, *J* = 13.3, 1.2 Hz), 3.74 (1H, br d, *J* = 9.1 Hz), 3.45 (1H, dd, *J* = 13.3, 3.3 Hz), 2.85 (1H, ddd, *J* = 12.2, 11.0, 9.1 Hz), 2.70 (1H, dd, *J* = 12.2, 3.0 Hz), 2.67−2.56 (2H, m), 2.35 (1H, dqd, *J* = 10.8, 7.3, 7.2 Hz), 1.89−1.76 (3H, m), 1.89 (1H, dd, *J* = 14.5, 4.6 Hz), 1.76 (3H, s), 1.74−1.67 (2H, m), 1.54 (1H, ddd, *J* = 12.8, 12.8, 10.8 Hz), 1.38−1.29 (1H, m), 1.33 (3H, s), 1.16 (3H, d, *J* = 7.3 Hz), 1.02 (3H, d, *J* = 7.1 Hz); ^{13}C NMR (101 MHz, $CDCl_3$) δ 214.9, 131.6, 128.9, 90.8, 77.0, 76.4, 67.3, 50.6, 45.2, 44.3, 44.0, 39.4,

37.2, 37.0, 35.4, 29.3, 26.2, 23.2, 18.8, 11.3; HRMS (ESI+) $[M+Na]^+$ calcd. for $C_{20}H_{30}O_3Na$ 341.2087, found 341.2078 (Δ −2.8 ppm).

(1*R*,2*R*,3*S*,5*S*,7*R*,8*S*,11*R*,14*Z*,17*R*)-5,8,11,15-Tetramethyl-10,18-dioxatetracyclo-[9.7.0.0^{2,7}.0^{3,17}]octadec-14-en-4-one (38a) and (1*R*,2*R*,3*S*,5*R*,7*R*,8*S*,11*R*,14*Z*,17*R*)-5,8,11,15-Tetramethyl-10,18-dioxatetracyclo-[9.7.0.0^{2,7}.0^{3,17}]octadec-14-en-4-one (38b).

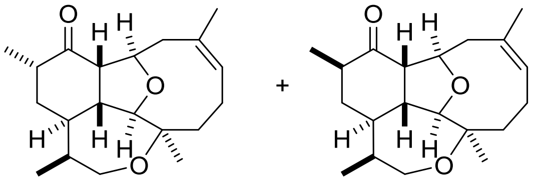

To a solution of ketone **34** (56.5 mg, 0.186 mmol) in anhydrous DMF (1.9 mL) in a sealed tube was added Bredereck's reagent [*tert*-butoxy bis(dimethylamino)methane] (0.12 mL, 0.58 mmol) in one portion. The resulting solution was heated to 100 °C and stirred at this temperature for 2 hours. The reaction mixture was cooled to room temperature and then diluted with ethyl acetate (12 mL) and water (5 mL). The phases were separated and the organic phase washed successively with water (3 × 3 mL), a 10 mol% aqueous solution of lithium chloride (5 mL) and brine (5 mL). The organic phase was dried ($MgSO_4$) and concentrated under reduced pressure. The residue was purified by flash column chromatography on silica gel (petroleum ether-ethyl acetate, gradient elution 1:1 → 1:5) to afford the enaminone **35** as a pale yellow solid, which was used in the next step without further purification.

To a stirred solution of enaminone **35** in anhydrous dichloromethane (2.3 mL) at −78 °C was added *i*-Bu_2AlH (0.22 mL of a 1.0 M solution in dichloromethane, 0.22 mmol) dropwise and the mixture was stirred for 20 minutes. The mixture was warmed to room temperature and allowed to stir for a further 1 hour. A solution of iodomethane (0.12 mL, 1.9 mmol) in anhydrous dichloromethane (3.5 mL) was added and the resulting solution was stirred for 1 hour. The mixture was cooled to 0 °C and a saturated aqueous solution of potassium sodium tartrate (5 mL) was added followed by diethyl ether (5 mL). The resulting mixture was warmed to room temperature and stirred vigorously for 1 hour (two clear phases were obtained). The phases were separated and the aqueous phase was extracted with diethyl ether (3 × 3 mL). The combined organic extracts were washed with brine (5 mL), dried ($MgSO_4$) and concentrated under reduced pressure to give crude enone **36**, which was used in the next step without purification.

To a stirred solution of Stryker's reagent [(triphenylphosphine)copper hydride hexamer] (0.47 mL of a 0.2 M solution in benzene, 94 μmol) in anhydrous and de-gassed toluene (3.3

mL) at −10 °C was added dropwise a solution of enone **36** in anhydrous and de-gassed toluene (6 mL) over 10 minutes. The resulting solution was stirred for 1 hour and the reaction was then quenched by the addition of water (5 mL). The mixture was warmed to room temperature and diluted with ethyl acetate (5 mL). The phases were separated and the aqueous phase was extracted with ethyl acetate (3 × 3 mL). The combined organic extracts were washed with brine (5 mL), dried ($MgSO_4$) and concentrated under reduced pressure. The residue was purified by flash column chromatography on silica gel (petroleum ether-ethyl acetate, 10:1) to afford the diastereoisomeric ketones **38a** (15 mg, 25% over 3 steps) and **38b** (22 mg, 37% over 3 steps) as colourless solids. Spectroscopic data were in accordance with that previously reported for compounds **38a** and **38b**.

Epimerisation of Ketone 38b.

To a stirred solution of ketone **38b** (39 mg, 0.12 mmol) in anhydrous methanol (2.5 mL) at room temperature was added potassium carbonate (20 mg, 0.14 mmol) and the resulting solution was stirred for 16 hours. The mixture was diluted with a saturated aqueous solution of ammonium chloride (5 mL) and ethyl acetate (5 mL). The phases were separated and the aqueous phase was extracted with ethyl acetate (3 × 3 mL). The combined organic extracts were washed with brine (5 mL), dried ($MgSO_4$) and concentrated under reduced pressure. The residue was purified by flash column chromatography on silica gel (petroleum ether-ethyl acetate, 10:1) to afford the diastereomeric ketones **38a** (15.9 mg, 41%) and **38b** (23 mg, 59%) as colourless solids.

Epimerisation of Ketone 38a.

To a stirred solution of ketone **38b** (12.6 mg, 39.6 μmol) in anhydrous methanol (0.8 mL) at room temperature was added potassium carbonate (6.6 mg, 48 μmol) and the resulting solution was stirred for 16 hours. The mixture was diluted with a saturated aqueous solution of ammonium chloride (3 mL) and ethyl acetate (5 mL). The phases were separated and the aqueous phase was extracted with ethyl acetate (3 × 3 mL). The combined organic extracts were washed with brine (5 mL), dried ($MgSO_4$) and concentrated under reduced pressure. The residue was purified by flash column chromatography on silica gel (petroleum ether-ethyl acetate, 10:1) to afford the diastereomeric ketones **38a** (4.5 mg, 36%) and **38b** (6.2 mg, 49%) as colourless solids.

11-Acetoxy-4-deoxyasbestinin D (1).

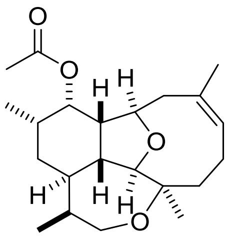

1

To a stirred solution of the ketone **38a** (23 mg, 72 μmol) in a mixture of methanol and dichloromethane (1:1, 1.4 mL) at 0 °C was added sodium borohydride (4.9 mg, 0.13 mmol) in one portion and the resulting mixture was stirred for 1 hour. The mixture was warmed to room temperature and stirred for a further 30 minutes. The reaction was quenched by the addition of a water (3 mL) and the mixture was diluted with ethyl acetate (3 mL). The phases were separated and the aqueous phase was extracted with ethyl acetate (3 × 2 mL). The combined organic extracts were washed with brine (3 mL), dried ($MgSO_4$) and concentrated under reduced pressure to give crude alcohol **39**, which was used in the subsequent reaction without purification.

To a stirred solution of alcohol **39**, DMAP (8.8 mg, 72 μmol) and distilled triethylamine (20 μL, 0.14 mmol) in anhydrous dichloromethane (1.4 mL) at 0 °C was added distilled acetic anhydride (0.65 mL of a 0.2 M solution in dichloromethane, 0.13 mmol). The mixture was warmed to room temperature and stirred for 16 hours. The reaction was quenched by the addition of a saturated aqueous solution of ammonium chloride (3 mL) and the mixture was diluted with diethyl ether (3 mL). The phases were separated and the aqueous phase was extracted with diethyl ether (3 × 2 mL). The combined organic extracts were washed with brine (3 mL), dried ($MgSO_4$) and concentrated under reduced pressure. The residue was purified by flash column chromatography on silica gel (pentane-ethyl acetate, 10:1) to give 11-acetoxy-4-deoxyasbestinin D (**1**) (23.8 mg, 88% over 2 steps) as a colourless oil. R_f = 0.67 (petroleum ether-ethyl acetate, 4:1); $[\alpha]_D^{26}$ −11.3 (*c* = 0.445, $CHCl_3$) {Lit.[2] $[\alpha]_D^{29}$ −2.29 (*c* = 1.31, $CHCl_3$); ν_{max} 2963, 2924, 2878, 1736, 910, 733 cm^{-1}; ^1H NMR (400 MHz, $CDCl_3$) δ 5.48 (1H, dddd, *J* = 9.8, 6.4, 1.4, 1.4 Hz), 5.31 (1H, dd, *J* = 5.2, 2.8 Hz), 4.10 (1H, ddd, *J* = 5.4, 2.8, 2.8 Hz), 3.88 (1H, dd, *J* = 8.8 Hz), 3.86 (1H, dd, *J* = 13.2, 1.2 Hz), 3.48 (1H, dd, *J* = 13.2, 3.3 Hz), 2.61−2.52 (1H, m), 2.50 (1H, dd, *J* = 14.4, 2.8 Hz, CH_2-C8a), 2.34 (1H, ddd, *J* = 10.8, 10.8, 8.8 Hz), 2.10 (3H, s), 2.08−1.83 (5H, m), 1.77−1.73 (2H, m), 1.75 (3H, s), 1.66−1.56 (1H, m), 1.53 (1H, ddd, *J* = 13.5, 13.5, 9.7 Hz), 1.36 (3H, s), 1.01 (1H, ddd, *J* = 13.5, 3.2, 1.5 Hz), 0.93 (3H, d, *J* = 7.2 Hz), 0.92 (3H, d, *J* = 7.1 Hz); ^{13}C NMR (101 MHz, $CDCl_3$) δ 171.4, 131.0, 128.9, 92.4, 81.2, 76.6, 73.7, 68.1, 46.0, 40.7, 38.7, 38.2, 37.7, 37.5, 31.7, 31.6, 29.1, 26.2, 23.6,

21.5, 18.1, 11.1; HRMS (ESI+) $[M+Na]^+$ calcd. for $C_{22}H_{34}O_4Na$ 385.2349, found 385.2341 (Δ −2.2 ppm).

(1*R*,2*R*,3*R*,4*S*,5*S*,7*R*,8*S*,11*R*,14*Z*,17*R*)-5,8,11,15-Tetramethyl-10,18-dioxatetracyclo-[9.7.0.0^{2,7}.0^{3,17}]octadec-14-en-4-ol (39).

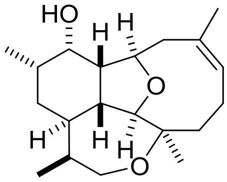

39

R_f = 0.41 (petroleum ether-ethyl acetate, 2:1); m.p. 126−128 °C; $[\alpha]_D^{17}$ +6.7 (*c* = 0.86, $CHCl_3$); ν_{max} 3292, 2920, 2890, 1721, 986, 733 cm^{-1}; ^1H NMR (400 MHz, $CDCl_3$) δ 5.49 (1H, dddd, *J* = 9.9, 6.2, 1.5, 1.5 Hz), 4.42 (1H, ddd, *J* = 5.0, 2.8, 2.8 Hz), 3.94 (1H, br d, *J* = 8.9 Hz), 3.86 (1H, br d, *J* = 13.2 Hz), 3.86−3.82 (1H, m), 3.45 (1H, dd, *J* = 13.2, 3.3 Hz), 2.69−2.57 (2H, m), 2.28 (1H, ddd, *J* = 10.9, 10.7, 8.9 Hz), 2.07 (1H, dddd, *J* = 13.6, 10.9, 4.4, 3.5 Hz), 1.97 (1H, dd, *J* = 14.4, 5.0 Hz), 1.92−1.79 (2H, m), 1.89 (1H, ddd, *J* = 10.7, 2.8, 2.4 Hz), 1.78−1.72 (2H, m), 1.77 (3H, s), 1.64 (1H, br d, *J* = 3.0 Hz), 1.60−1.54 (1H, m), 1.52 (1H, ddd, *J* = 13.6, 13.5, 10.0 Hz), 1.35 (3H, s), 1.08 (3H, d, *J* = 7.3 Hz), 1.01 (1H, ddd, *J* = 13.5, 3.5, 1.8 Hz), 0.90 (3H, d, *J* = 7.1 Hz); ^{13}C NMR (101 MHz, $CDCl_3$) δ 131.0, 128.9, 92.7, 81.9, 76.4, 72.7, 68.1, 47.2, 40.7, 38.8, 37.9, 37.8, 37.7, 32.3, 31.8, 29.3, 26.1, 23.6, 18.5, 11.0; HRMS (ESI+) $[M+Na]^+$ calcd. for $C_{20}H_{32}O_3Na$ 343.2244, found 343.2249 (Δ +1.6 ppm).

4-Deoxyasbestinin C (2).

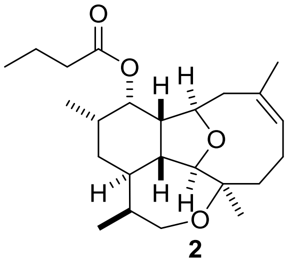

2

To a stirred solution of alcohol **39** (18 mg, 56 μmol), DMAP (8.2 mg, 67 μmol) and distilled *N*,*N*-diisopropylethylamine (0.23 mL, 1.3 mmol) in anhydrous dichloromethane (1.2 mL) at 0 °C was added butyric anhydride (0.34 mL of a 0.5 M solution in dichloromethane, 0.17 mmol). The resulting solution was warmed to room temperature and stirred for 16 hours. The reaction was quenched by the addition of a saturated aqueous solution of ammonium chloride (5 mL) and the mixture was diluted with diethyl ether (3 mL). The phases were separated and the aqueous phase was extracted with diethyl ether (3 × 3 mL). The combined organic extracts

were washed with brine (5 mL), dried ($MgSO_4$) and concentrated under reduced pressure. The residue was purified by flash column chromatography on silica gel (pentane-ethyl acetate, 10:1) to afford 4-deoxyasbestinin C (**2**) (16.5 mg, 75%) as a colourless oil. R_f = 0.81 (petroleum ether-ethyl acetate, 3:1); $[\alpha]_D^{20}$ −16.8 (*c* = 0.375, $CHCl_3$) {Lit.[2] $[\alpha]_D^{29}$ −1.2 (*c* = 0.84, $CHCl_3$); ν_{max} 2963, 2932, 2874, 1732 cm^{-1}; ^1H NMR (400 MHz, $CDCl_3$) δ 5.51–5.44 (1H, m), 5.33 (1H, dd, *J* = 5.1, 2.8 Hz), 4.08 (1H, ddd, *J* = 5.0, 2.8, 2.8 Hz), 3.88 (1H, d, *J* = 8.9 Hz), 3.86 (1H, d, *J* = 13.3 Hz), 3.49 (1H, dd, *J* = 13.3, 3.3 Hz), 2.63−2.50 (2H, m), 2.41–2.26 (3H, m), 2.10–2.01 (1H, m), 1.98 (1H, ddd, *J* = 10.7, 2.8, 2.8 Hz), 1.95 (1H, dd, *J* = 14.5, 5.0 Hz), 1.93–1.84 (2H, m), 1.77–1.48 (5H, m), 1.76 (3H, s), 1.52 (1H, *J* = 13.5, 13.5, 9.7), 1.35 (3H, s), 1.01 (1H, dd, J = 13.5, 3.2, 1.5 Hz), 0.98 (3H, t, *J* = 7.4 Hz), 0.92 (3H, d, *J* = 7.3 Hz), 0.92 (3H, d, *J* = 7.0 Hz); ^{13}C NMR (101 MHz, $CDCl_3$) δ 174.0, 131.1, 128.9, 92.3, 81.3, 76.5, 73.4, 68.1, 45.9, 40.8, 38.9, 38.3, 37.6, 37.6, 36.9, 31.8, 31.6, 29.2, 26.3, 23.4, 18.7, 18.2, 13.9, 11.1; HRMS (ESI+) $[M+Na]^+$ calcd. for $C_{24}H_{38}O_4Na$ 413.2662, found 413.2646 (Δ −3.8 ppm).

(1*R*,2*R*,3*S*,4*S*,5*S*,7*R*,8*S*,11*R*,14*S*,16*R*,18*R*)-5,8,11,16-Tetramethyl-10,15,19-trioxapenta-cyclo[9.8.0^{2,7}.0^{3,18}.0^{14,16}]nonadecan-4-yl acetate (40a) and (1*R*,2*R*,3*S*,4*S*,5*S*,7*R*,8*S*,11*R*,14*R*,16*S*,18*R*)-5,8,11,16-Tetramethyl-10,15,19-trioxapenta-cyclo[9.8.0^{2,7}.0^{3,18}.0^{14,16}]nonadecan-4-yl acetate (40b).

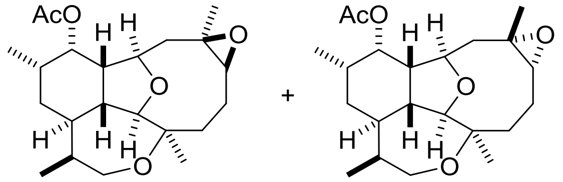

40a **40b**

*Epoxidation of 11-acetoxy-4-deoxyasbestinin D (*1*) with m-CPBA*

To a stirred solution of 11-acetoxy-4-deoxyasbestinin D (**1**) (22 mg, 61 μmol) in anhydrous dichloromethane (1.2 mL) at 0 °C was added *m*-CPBA solution (0.24 mL of a 0.5 M solution in dichloromethane, 0.12 mmol). The resulting solution was stirred for 2.5 hours and the reaction was quenched at 0 °C by the addition of a saturated aqueous solution of sodium thiosulfate (3 mL) and then warmed to room temperature. The mixture was diluted with ethyl acetate (5 mL) and the phases were separated. The aqueous phase was extracted with ethyl acetate (3 × 3 mL) and the combined organic extracts were washed with brine (5 mL), dried ($MgSO_4$) and concentrated under reduced pressure. The residue was purified by flash column chromatography on silica gel (petroleum ether-ethyl acetate, gradient elution 10:1 → 1:1) to deliver the diastereoisomeric epoxides **40a** (13.6 mg, 59%) and **40b** (5.3 mg, 23%) as colourless solids.

*Epoxidation of 11-acetoxy-4-deoxyasbestinin D (**1**) with the Shi Epoxidation Catalyst (unnatural enantiomer)*

To a stirred solution of 11-acetoxy-4-deoxyasbestinin D (**1**) (16 mg, 44 µmol) and unnatural Shi ketone (3.8 mg, 15 µmol) in a mixture of acetonitrile and dimethoxyethane (1:2, 0.9 mL) was added buffer (0.48 mL of a 0.05 M solution of $Na_2B_4O_7•10H_2O$ in 4 × 10^{-4} M aqueous Na_2(EDTA), 24 µmol) followed by tetrabutylammonium hydrogen sulfate (5.0 µL of a 0.4 M solution in water, 2.0 µmol). The resulting solution was cooled to 0 °C and Oxone™ (1.4 mL of a 0.07 M solution in 4 × 10^{-4} M aqueous Na_2(EDTA), 0.098 mmol) and potassium carbonate (0.70 mL of a 0.4 M solution in water, 0.28 mmol) were added simultaneously. The mixture was stirred for 1 hour and then diluted with water (5 mL) and ethyl acetate (10 mL). The phases were separated and the aqueous phase was extracted with ethyl acetate (3 × 5 mL). The combined organic extracts were washed with brine (10 mL), dried ($MgSO_4$) and concentrated under reduced pressure. The residue was purified by flash column chromatography on silica gel (pentane-ethyl acetate, gradient elution 10:1 → 2:1) to give the diastereoisomeric epoxides **40a** (12.5 mg, 75%) and **40b** (1.0 mg, 6%) as colourless solids.

*Epoxidation of 11-acetoxy-4-deoxyasbestinin D (**1**) with the Shi Epoxidation Catalyst (natural enantiomer)*

To a stirred solution of 11-acetoxy-4-deoxyasbestinin D (**1**) (27.5 mg, 75.9 µmol) and natural Shi ketone (5.9 mg, 23 µmol) in a mixture of acetonitrile and dimethoxyethane (1:2, 1.2 mL) was added buffer (1.2 mL of a 0.05 M solution of $Na_2B_4O_7•10H_2O$ in 4 × 10^{-4} M aqueous Na_2(EDTA), 0.060 mmol) followed by tetrabutylammonium hydrogen sulfate (7.5 µL of a 0.4 M solution in water, 3.0 µmol). The resulting solution was cooled to 0 °C and Oxone™ (1.9 mL of a 0.4 M solution in 4 × 10^{-4} M aqueous Na_2(EDTA), 0.76 mmol) and potassium carbonate (1.1 mL of a 0.4 M solution in water, 0.44 mmol) were added simultaneously. The mixture was stirred for 1 hour and then diluted with water (5 mL) and ethyl acetate (10 mL). The phases were separated and the aqueous phase was extracted with ethyl acetate (3 × 5 mL). The combined organic extracts were washed with brine (10 mL), dried ($MgSO_4$) and concentrated under reduced pressure. The residue was purified by flash column chromatography on silica gel (pentane-ethyl acetate, gradient elution 10:1 → 2:1) to give the diastereomeric epoxides **40a** (3.6 mg, 13%) and **40b** (20.4 mg, 71%) as colourless solids.

40a: R_f = 0.10 (petroleum ether-ethyl acetate, 2:1); m.p. 156−158 °C; $[\alpha]_D^{19}$ −37.7 (*c* = 0.247, $CHCl_3$); ν_{max} 2959, 2928, 2878, 1736, 733 cm^{-1}; ^1H NMR (400 MHz, $CDCl_3$) δ 5.41 (1H, dd, *J* = 6.3, 1.8 Hz), 4.26 (1H, dd, *J* = 11.7, 4.8 Hz), 3.88 (1H, d, *J* = 10.1 Hz), 3.83 (1H, d, *J* = 13.1 Hz), 3.60 (1H, dd, *J* = 13.1, 2.2 Hz), 2.80 (1H, ddd, *J* = 10.1, 9.6, 9.4 Hz, CH-C1), 2.74 (1H, dd, *J* = 9.6, 3.1 Hz), 2.20−2.11 (1H, m), 2.08 (3H, s), 2.03−1.96 (1H, m), 2.02 (1H, dd, *J* = 13.6, 4.8 Hz), 1.91−1.74 (3H, m), 1.71−1.48 (5H, m), 1.34 (3H, s), 1.34 (3H, s), 1.05 (1H, dd,

J = 13.5, 2.2 Hz), 0.98 (3H, d, J = 7.1 Hz), 0.92 (3H, d, J = 7.2 Hz); ^{13}C NMR (101 MHz, $CDCl_3$) δ 171.1, 94.4, 79.3, 78.5, 72.8, 70.3, 66.4, 59.5, 50.0, 43.4, 39.6, 37.6, 36.4, 34.3, 32.0, 31.3, 27.8, 22.5, 21.8, 21.5, 16.7, 11.2; HRMS (ESI+) [M+Na]$^+$ calcd. for $C_{22}H_{34}O_5Na$ 401.2298, found 401.2304 (Δ −1.4 ppm). **40b**: R_f = 0.18 (petroleum ether-ethyl acetate, 2:1); m.p. 167−169 °C; $[\alpha]_D^{17}$ +6.74 (c = 0.860, $CHCl_3$); ν_{max} 2963, 2928, 2874, 1736, 991, 930, 872, 733, 702, 652 cm^{-1}; ^{1}H NMR (400 MHz, $CDCl_3$) δ 5.30 (1H, dd, J = 5.1, 3.2 Hz), 4.06 (1H, ddd, J = 3.2, 3.2, 3.2 Hz), 3.88 (1H, br d, J = 8.8 Hz), 3.85 (1H, br d, J = 13.2 Hz), 3.50 (1H, dd, J = 13.2, 3.5 Hz), 2.70 (1H, dd, J = 10.9, 3.8 Hz), 2.37 (1H, ddd, J = 10.8, 3.2, 3.2 Hz), 2.31 (1H, ddd, J = 10.8, 10.8, 8.8 Hz), 2.12 (3H, s), 2.07−1.83 (7H, m), 1.72−1.61 (2H, m), 1.55 (1H, ddd, J = 13.5, 13.5, 9.7 Hz), 1.43 (3H, s), 1.35 (3H, s), 1.07 (1H, ddd, J = 13.5, 3.2, 1.6 Hz), 0.95 (3H, d, J = 7.6 Hz), 0.93 (3H, d, J = 7.4 Hz); ^{13}C NMR (101 MHz, $CDCl_3$) δ 171.4, 92.1, 80.7, 76.0, 73.7, 67.8, 67.0, 59.2, 44.6, 40.8, 39.9, 38.1, 37.5, 33.7, 31.8, 31.6, 28.7, 25.5, 23.9, 21.4, 18.2, 11.0; HRMS (ESI+) [M+Na]$^+$ calcd. for $C_{22}H_{34}O_5Na$ 401.2298, found 401.2286 (Δ +3.1 ppm).

Asbestinin-20 (6).

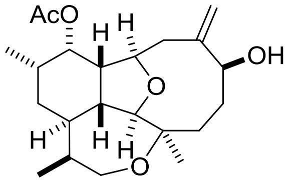

6

Method A: Silylation and ring opening of epoxide **40a**

To a stirred solution of epoxide **40a** (18 mg, 0.048 mmol) in anhydrous dichloromethane (1.0 mL) at 0 °C was added 2,6-lutidine (28 μl, 0.24 mmol) followed by *tert*-butyldimethylsilyl trifluoromethanesulfonate (0.16 mL of a 0.5 M solution in THF, 0.080 mmol). The mixture was stirred for 3 hours and then the reaction was quenched by the addition of a saturated aqueous solution of ammonium chloride (3 mL). The mixture was diluted with ethyl acetate (5 mL) and the phases were separated. The aqueous phase was extracted with ethyl acetate (3 × 3 mL) and the combined organic extracts were washed with brine (5 mL), dried ($MgSO_4$) and concentrated under reduced pressure. The resulting crude TBS-protected allylic alcohol was used in the subsequent step without purification.

To a stirred solution of TBS-protected allylic alcohol in THF (1.0 mL) at room temperature was added tetrabutylammonium fluoride (0.19 mL of a 1.0 M solution in THF, 0.19 mmol) in one portion. The mixture was stirred for 16 hours and then diluted with diethyl ether (5 mL) and water (3 mL). The phases were separated and the aqueous phase extracted with diethyl ether (3 × 3 mL). The combined organic extracts were washed with brine (5 mL), dried ($MgSO_4$) and

concentrated under reduced pressure. The residue was purified by flash column chromatography on silica gel (hexane-ethyl acetate, gradient elution 2:1 → 1:1) to afford asbestinin-20 (**6**) (7.0 mg, 39%) as a colourless solid.

Method B: Ring opening of epoxide **40a** *with Au-TiO₂*

To a solution of epoxide **40a** (8.6 mg, 23 μmol) in anhydrous dichloroethane (0.9 mL) was added Au/TiO_2 (2.3 mg) in one portion. The resulting suspension was stirred at 80 °C in a sealed vial for 2 days. The reaction mixture was cooled to room temperature and diluted with dichloromethane (3 mL). The mixture was filtered through a pad of celite and the filter cake was washed with dichloromethane (3 mL). The filtrate was concentrated under reduced pressure and the residue was purified by flash column chromatography on silica gel (pentane-ethyl acetate, gradient elution 1:1 → 1:3) to afford asbestinin 20 (**6**) (3.0 mg, 35%) as a colourless solid. R_f = 0.40 (petroleum ether-ethyl acetate, 1:2); m.p. 185−187 °C; $[\alpha]_D^{16}$ −14.4 (*c* = 0.250, $CHCl_3$) {Lit.[4] $[\alpha]_D^{25}$ −16.7 (*c* = 5.20, $CHCl_3$); ν_{max} 3433, 2959, 2924, 2874, 1736 cm^{-1}; ^{1}H NMR (500 MHz, $CDCl_3$) δ 5.37−5.33 (1H, m), 5.36 (1H, dd, *J* = 5.3, 2.2 Hz), 5.05 (1H, br s), 4.27 (1H, br s), 4.17 (1H, ddd, *J* = 4.5, 4.5, 1.8 Hz), 3.85 (1H, d, *J* = 8.7 Hz), 3.76 (1H, d, *J* = 13.0 Hz), 3.45 (1H, dd, *J* = 13.0, 2.9 Hz), 2.51−2.42 (1H, m), 2.32−2.16 (4H, m), 2.16−2.07 (1H, m), 2.09 (3H, s), 1.91−1.78 (3H, m,), 1.61−1.55 (1H, m), 1.50 (1H, dd, *J* = 13.5, 13.5, 9.4 Hz), 1.48 (1H, d, *J* = 3.1 Hz), 1.40−1.30 (1H, m), 1.30 (3H, s), 1.02 (1H, dd, *J* = 13.5, 2.4 Hz), 0.93 (3H, d, *J* = 7.2 Hz), 0.90 (3H, d, *J* = 7.1 Hz); ^{13}C NMR (126 MHz, $CDCl_3$) δ 171.5, 148.5, 115.0, 94.3, 83.1, 76.8, 76.2, 74.0, 67.7, 46.4, 39.3, 39.0, 38.2, 36.9, 31.7, 31.5, 29.2, 27.4, 23.6, 21.5, 17.7, 11.1; HRMS (ESI+) $[M+Na]^+$ calcd. for $C_{22}H_{34}O_5Na$ 401.2298, found 401.2283 (Δ −3.8 ppm).

6-*epi*-Asbestinin-20 (9).

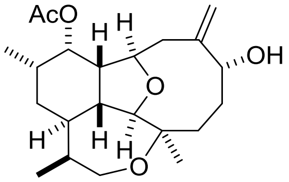

9

To a stirred solution of epoxide **40b** (20 mg, 0.053 mmol) in anhydrous dichloromethane (1.1 mL) at 0 °C was added 2,6-lutidine (30 μl, 0.27 mmol) followed by *tert*-butyldimethylsilyl trifluoromethanesulfonate (0.16 mL of a 0.5 M solution in THF, 0.080 mmol) and the mixture was stirred for 3 hours. The reaction was quenched by the addition of a saturated aqueous solution of ammonium chloride (3 mL) and the mixture was diluted with ethyl acetate (5 mL). The phases were separated and the aqueous phase was extracted with ethyl acetate (3 × 3 mL). The combined organic extracts were washed with brine (5 mL), dried ($MgSO_4$) and

concentrated under reduced pressure to give crude TBS protected allylic alcohol, which was used in the next step without purification.

To a stirred solution of TBS protected allylic alcohol in THF (1.1 mL) at room temperature was added tetrabutylammonium fluoride (0.2 mL of a 1.0 M solution in THF, 0.2 mmol) in one portion and the mixture was stirred for 16 hours. The mixture was diluted with diethyl ether (5 mL) and water (3 mL) and the phases were separted. The aqueous phase was extracted with diethyl ether (3 × 3 mL) and the combined organic extracts were washed with brine (5 mL), dried ($MgSO_4$) and concentrated under reduced pressure. The residue was purified by flash column chromatography on silica gel (pentane-ethyl acetate, gradient elution 1:1 → 1:2) to provide 6-*epi*-asbestinin 20 (**9**) (12.8 mg, 64%) as a colourless solid. R_f = 0.21 (petroleum ether-ethyl acetate, 1:2); m.p. 212−214 °C; $[\alpha]_D^{20}$ −51.2 (*c* = 0.285, $CHCl_3$) {Lit.[3] $[\alpha]_D^{20}$ −30 (*c* = 0.50, $CHCl_3$); ν_{max} 3429, 2957, 2926, 2876, 1736, 968 cm^{-1}; ^1H NMR (400 MHz, $CDCl_3$) δ 5.35 (1H, dd, *J* = 5.4, 2.5 Hz), 5.22 (1H, br s), 5.08 (1H, br s), 4.14 (1H, ddd, *J* = 4.7, 2.5, 2.5 Hz), 4.03 (1H, dd, *J* = 11.7, 4.0 Hz), 3.85 (1H, d, *J* = 8.5 Hz), 3.76 (1H, d, *J* = 13.0 Hz), 3.42 (1H, dd, *J* = 13.0, 3.2 Hz), 2.74 (1H, dd, *J* = 14.0, 4.7 Hz), 2.30 (1H, ddd, *J* = 10.5, 10.5, 8.5 Hz), 2.19 (1H, ddd, *J* = 10.5, 2.5, 2.5 Hz), 2.13−2.02 (2H, m), 2.11 (3H, s), 1.97 (1H, dd, *J* = 14.0, 2.5 Hz), 1.87 (1H, dddd, *J* = 13.5, 10.5, 4.5, 3.1 Hz), 1.76−1.65 (1H, m), 1.63−1.35 (5H, m), 1.32 (3H, s), 1.01 (1H, dd, *J* = 13.5, 3.1 Hz), 0.93 (3H, d, *J* = 7.2 Hz), 0.86 (3H, d, *J* = 7.1 Hz); ^{13}C NMR (101 MHz, $CDCl_3$) δ 171.3, 147.6, 119.6, 93.9, 82.0, 79.1, 76.0, 74.0, 67.4, 45.8, 39.1, 37.9, 36.8, 35.4, 31.6, 31.6, 31.4, 27.8, 23.5, 21.3, 17.7, 10.9; HRMS (ESI+) $[M+Na]^+$ calcd. for $C_{22}H_{34}O_5Na$ 401.2298, found 401.2284 (Δ −3.6 ppm).

Asbestinin-10 (14).

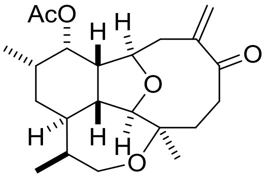

14

To a stirred solution of allylic alcohols **6** and **9** (2:1 mixture, 16 mg, 42 μmol) in anhydrous dichloromethane (1.0 mL) at room temperature was added anhydrous pyridine (14 μL, 0.17 mmol) followed by Dess-Martin periodinane (32 mg, 75 μmol) in one portion. The reaction mixture was stirred for 2 hours and then diluted with ethyl acetate (5 mL). The reaction was quenched by the addition of a saturated aqueous solution of sodium thiosulfate (3 mL) and the mixture was stirred for 5 minutes. A saturated aqueous solution of sodium bicarbonate (1 mL) was added and the resulting mixture was stirred vigorously for 30 minutes. The phases were separated and the aqueous phase extracted with ethyl acetate (3 × 3 mL). The combined

organic extracts were washed with brine (5 mL), dried ($MgSO_4$) and concentrated under reduced pressure. The residue was purified by flash column chromatography on silica gel (pentane-diethyl ether, 10:1) to give asbestinin-10 (**14**) (12 mg, 75%) as a colourless oil. R_f = 0.23 (petroleum ether-ethyl acetate, 2:1) ; $[\alpha]_D^{23}$ −78 (*c* = 0.13, $CHCl_3$) {Lit.[5] $[\alpha]_D^{25}$ −81.5 (*c* = 0.760, $CHCl_3$); ν_{max} 2961, 2928, 2874, 1736, 1686, 916 cm^{-1}; ^1H NMR (400 MHz, $CDCl_3$) δ 5.28–5.26 (1H, m), 5.26 (1H, dd, *J* = 5.0, 2.8 Hz), 5.18 (1H, br s), 4.04 (1H, dd, *J* = 6.7, 4.1 Hz), 3.77 (1H, d, *J* = 9.4 Hz), 3.75 (1H, d, *J* = 13.0 Hz), 3.49 (1H, dd, *J* = 13.0, 3.0 Hz), 3.36 (1H, ddd, *J* = 13.2, 6.8, 1.5 Hz), 2.81 (1H, ddd, *J* = 15.5, 8.1, 2.1 Hz), 2.49 (1H, ddd, *J* = 15.5 12.5, 2.0 Hz), 2.41 (1H, ddd, *J* = 11.2, 10.8, 9.4 Hz), 2.30 (1H, ddd, *J* = 14.9, 12.5, 2.1 Hz), 2.15 (1H, br d, *J* = 13.2 Hz), 2.10–1.98 (2H, m), 2.09 (3H, s), 1.89 (1H, dddd, *J* = 14.0, 10.8, 4.0, 3.6 Hz), 1.67 (1H, ddd, *J* = 14.9, 8.1, 2.0 Hz), 1.63–1.51 (2H, m), 1.28 (3H, s), 1.05 (1H, ddd, *J* = 13.6, 3.6, 1.9 Hz), 0.94 (3H, d, *J* = 7.2 Hz), 0.93 (3H, d, *J* = 7.1 Hz); ^1H NMR (400 MHz, C_6D_6) δ 5.23 (1H, dd, *J* = 5.0, 2.9 Hz), 4.79 (1H, br s), 4.77 (1H, br s), 4.07 (1H, dd, *J* = 6.7, 4.0 Hz), 3.77 (1H, d, *J* = 9.4 Hz), 3.49–3.42 (2H, m), 3.33 (1H, dd, *J* = 13.0, 3.0 Hz), 2.63–2.48 (2H, m), 2.39–2.24 (2H, m), 1.82–1.71 (4H, m), 1.70 (3H, s), 1.66–1.57 (1H, m), 1.29–1.18 (2H, m), 1.28 (3H, s), 0.88 (3H, d, *J* = 7.1 Hz), 0.88 (3H, d, *J* = 7.1 Hz), 0.75 (1H, ddd, *J* = 13.5, 3.7, 2.0 Hz); ^{13}C NMR (101 MHz, $CDCl_3$) δ 206.5, 171.1, 146.7, 114.1, 93.3, 80.0, 73.1, 68.2, 48.0, 41.6, 40.2, 37.6, 37.4, 36.7, 35.9, 31.7, 31.2, 24.1, 21.2, 18.1, 11.0 (peak for C-3 was not observed); ^{13}C NMR (101 MHz, C_6D_6) δ 203.0, 169.1, 146.8, 111.6, 92.5, 79.2, 76.4, 71.8, 66.7, 47.1, 40.6, 39.1, 36.7, 36.6, 36.0, 35.1, 30.7, 30.3, 23.0, 19.4, 17.1, 10.1; HRMS (ESI+) $[M+Na]^+$ calcd. for $C_{22}H_{32}O_5Na$ 399.2142, found 399.2131 (Δ −2.8 ppm).

Asbestinin-20 (6) and 6-*epi*-Asbestinin-20 (9).

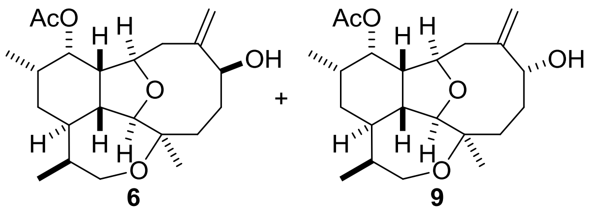

To a stirred solution of asbestinin-10 (**14**) (10 mg, 27 μmol) in a mixture of methanol and dichloromethane (1:1, 0.6 mL) at room temperature was added cerium(III) chloride heptahydrate (20 mg, 54 μmol). The mixture was stirred for 15 minutes and then cooled to −78 °C. Sodium borohydride (2.0 mg, 0.053 mmol) was added in one portion and the resulting mixture was stirred for 2 hours. The reaction mixture was warmed to room temperature and stirred for a further 20 minutes. The reaction was quenched by the addition of a 50% aqueous solution of ammonium chloride (2 mL) and the resulting mixture was diluted with ethyl acetate

(5 mL). The phases were separated and and the aqueous phase was extracted with ethyl acetate (3 × 2 mL). The combined organic extracts were washed with brine (3 mL), dried ($MgSO_4$) and concentrated under reduced pressure. The residue was purified by flash column chromatography on silica gel (pentane-diethyl ether, gradient elution 1:2 → 1:5) to deliver 6-*epi*-asbestinin-20 (**9**) (2.0 mg, 20%) and asbestinin-20 (**6**) (6.4 mg, 64%) as colourless solids. Spectroscopic data is in accordance with that previously reported for the alcohols **6** and **9**.

(1*R*,2*R*,3*S*,4*S*,5*S*,7*R*,8*S*,11*R*,14*S*,15*R*,17*R*)-14,15-Dihydroxy-5,8,11,15-tetramethyl-10,18-dioxatetracyclo[9.7.0.0^{2,7}.0^{3,17}]octadecan-4-yl acetate (41a) and (1*R*,2*R*,3*S*,4*S*,5*S*,7*R*,8*S*,11*R*,14*R*,15*S*,17*R*)-14,15-Dihydroxy-5,8,11,15-tetramethyl-10,18-dioxatetracyclo[9.7.0.0^{2,7}.0^{3,17}]octadecan-4-yl acetate (41b).

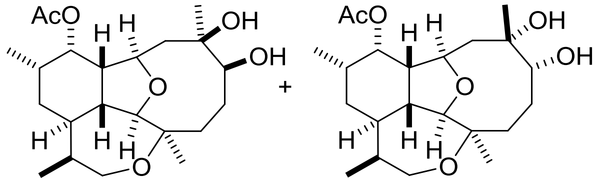

To a stirred of 11-acetoxy-4-deoxyasbestinin D (**1**) (16.6 mg, 45.8 μmol) in a mixture of THF and water (1:1, 1.2 mL) at 0 °C was added *N*-methylmorpholine oxide (54 mg, 0.46 mmol) followed by osmium tetroxide (15 μL of a 4% wt solution in water, 2.4 μmol). The reaction mixture was stirred for 1.5 hours and then the reaction was quenched by the addition of a saturated aqueous solution of sodium thiosulfate (2 mL). The resulting suspension was stirred vigorously for 1 hour and then diluted with ethyl acetate (5 mL). The phases were separated and the aqueous phase was extracted with ethyl acetate (3 × 2 mL). The combined organic extracts were washed with brine (4 mL), dried ($MgSO_4$) and concentrated under reduced pressure. The residue was purified by flash column chromatography on silica gel (pentane-ethyl acetate, gradient elution 1:2 → 1:5) to give the diols **41a** and **41b** (5.7:1, 10.2 mg, 56%) as a colourless solid. The diols were later found to be separable by flash column chromatography (dichloromethane-methanol, gradient elution 19:1 → 5:1) and a pure sample of the crystalline diol **41a** was characterised fully. R_f = 0.13 (petroleum ether-ethyl acetate, 1:2); m.p. 209−211 °C; $[\alpha]_D^{20}$ −8.89 (*c* = 0.450, $CHCl_3$); ν_{max} 3431, 2963, 2922, 2876, 1734, 991, 974, 930, 918, 731 cm^{-1}; ^{1}H NMR (500 MHz, $CDCl_3$) δ 5.39 (1H, dd, *J* = 5.7, 2.0 Hz), 4.22 (1H, dd, *J* = 12.4, 3.9 Hz), 3.83−3.77 (2H, m), 3.47 (1H, dd, *J* = 13.1, 2.7 Hz), 2.56 (1H, ddd, *J* = 9.4, 9.4, 9.4 Hz), 2.22−1.99 (4H, m), 2.08 (3H, s), 1.96−1.74 (5H, m), 1.65−1.57 (2H, m), 1.52−1.43 (2H, m), 1.37 (1H, br s), 1.30 (3H, s), 1.30 (3H, s), 1.03 (1H, dd, *J* = 13.4, 2.7 Hz), 0.92 (3H, d, *J* = 7.2 Hz), 0.92 (3H, d, *J* = 7.2 Hz); ^{13}C NMR (126 MHz, $CDCl_3$) δ 171.5, 93.4,

79.3, 77.5, 75.5, 73.5, 68.0, 48.5, 38.3, 37.6, 37.6, 31.5, 31.4, 29.8, 25.7, 22.4, 21.6, 17.4, 11.1 (peaks for three carbons were not observed); HRMS (ESI+) $[M+Na]^+$ calcd. for $C_{22}H_{36}O_6Na$ 419.2404, found 419.2387 (Δ −4.0 ppm).

7-*epi*-Asbestinin-21 (16) and Asbestinin-21 (42).

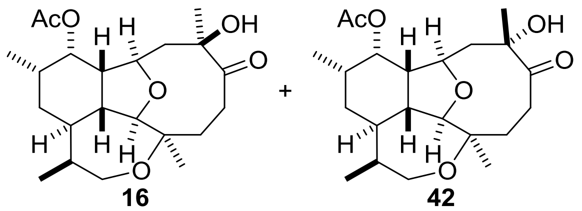

To a stirred solution of the diastereomeric diols **41a** and **41b** (10.2 mg, 25.7 μmol) in anhydrous dichloromethane (1.0 mL) at room temperature was added pyridine (80 μL, 0.10 mmol) and Dess-Martin periodinane (20 mg, 47 μmol). The resulting mixture was stirred for 2 hours then diluted with diethyl ether (3 mL). The reaction was quenched by the addition of saturated aqueous solution of sodium thiosulfate (2 mL) and the mixture was stirred for 5 minutes. A saturated aqueous solution of sodium bicarbonate (0.5 mL) was added and the resulting mixture was stirred vigorously for 30 minutes. The phases were separated and the aqueous phase was extracted with diethyl ether (3 × 2 mL). The combined organic extracts were washed with brine (3 mL), dried ($MgSO_4$) and concentrated under reduced pressure. The residue was purified by flash column chromatography on silica gel (pentane-ethyl acetate, gradient elution 1:1 → 1:3) to deliver 7-*epi*-asbestinin-21 (**16**) (6.8 mg, 67%) as a colourless oil and asbestinin-21 (**42**) (1.4 mg, 14%) as a colourless solid. **16**: R_f = 0.49 (petroleum ether-ethyl acetate, 1:3) ; $[\alpha]_D^{20}$ −25.6 (*c* = 0.245, $CHCl_3$); ν_{max} 3451, 2963, 2932, 2876, 1736, 1690, 997, 963, 918, 873, 731, 646 cm^{-1}; ^1H NMR (400 MHz, $CDCl_3$) δ 5.23 (1H, dd, *J* = 5.1, 2.6 Hz), 3.92 (1H, ddd, *J* = 6.1, 4.1, 1.8 Hz), 3.78 (1H, br d, *J* = 13.1 Hz), 3.77 (1H, d, *J* = 9.7 Hz), 3.55 (1H, dd, *J* = 13.1, 2.8 Hz), 3.32–3.21 (1H, br s), 2.83 (1H, ddd, *J* = 10.4, 9.7, 9.5 Hz), 2.52–2.46 (1H, m), 2.46 (1H, dd, *J* = 14.5, 6.1 Hz), 2.41–2.30 (1H, m), 2.12–1.98 (2H, m), 2.07 (3H, s), 1.93–1.79 (4H, m), 1.63–1.51 (2H, m), 1.38 (3H, s), 1.32 (3H, s), 1.03 (1H, dd, *J* = 13.5, 2.0 Hz), 0.99 (3H, d, *J* = 7.1 Hz), 0.92 (3H, d, *J* = 7.2 Hz); ^{13}C NMR (101 MHz, $CDCl_3$) δ 214.2, 171.4, 93.7, 79.8, 78.9, 76.7, 73.8, 69.1, 47.1, 47.0, 40.1, 37.5, 37.1, 37.0, 32.8, 32.1, 31.3, 28.4, 25.7, 21.4, 18.0, 11.2; HRMS (ESI+) $[M+Na]^+$ calcd. for $C_{22}H_{34}O_6Na$ 417.2248, found 417.2235 (Δ −3.1 ppm). **42**: R_f = 0.44 (petroleum ether-ethyl acetate, 1:2); $[\alpha]_D^{21}$ −7.2 (*c* = 0.10, $CHCl_3$) {Lit.[4] $[\alpha]_D^{24}$ −27.1 (*c* = 1.8, $CHCl_3$)}; ν_{max} 3439, 2964, 2926, 1736, 1686 cm^{-1}; ^1H NMR (500 MHz, $CDCl_3$) δ 5.34 (1H, dd, *J* = 5.6, 2.3 Hz), 4.39 (1H, s), 4.28 (1H, dd, *J* = 11.5, 3.4 Hz), 3.82 (1H, d, *J* = 8.8 Hz), 3.78 (1H, br d, *J* = 13.0 Hz), 3.49 (1H, dd, *J* = 13.0, 2.8 Hz), 2.75 (1H, ddd, *J* = 14.6, 14.6, 3.9 Hz), 2.67−2.61 (1H, m), 2.57 (1H, ddd, *J* = 10.0, 10.0,

8.8 Hz), 2.30 (1H, ddd, J = 14.6, 14.6, 3.4 Hz), 2.26−2.18 (1H, m), 2.12–2.03 (2H, m), 2.05 (3H, s), 1.93–1.85 (2H, m), 1.64–1.45 (3H, m), 1.33 (3H, s), 1.30 (3H, s), 1.04 (1H, dd, J = 13.6, 2.6 Hz), 0.94 (3H, d, J = 7.1 Hz), 0.90 (3H, d, J = 7.3 Hz); ^{13}C NMR (126 MHz, $CDCl_3$) δ 210.5, 170.9, 93.5, 78.2, 77.0, 76.2, 72.7, 68.1, 49.3, 47.5, 37.9, 37.9, 36.4, 36.1, 33.9, 31.4, 31.4, 27.7, 22.5, 21.4, 17.2, 10.9; HRMS (ESI+) $[M+Na]^+$ calcd. for $C_{22}H_{34}O_6Na$ 417.2248, found 417.2233 (Δ −3.5 ppm).

7-*epi*-Asbestinin-21 (16).

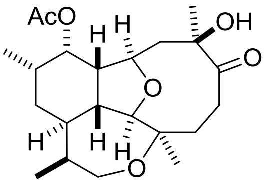

16

To a stirred solution of diol **41a** (10.5 mg, 26.4 μmol) in anhydrous dichloromethane (0.5 mL) at room temperature was added pyridine (8.4 μL, 0.10 mmol) and Dess-Martin periodinane (20 mg, 47 μmol). The mixture was stirred for 2 hours and then diluted with diethyl ether (5 mL). The reaction was quenched by the addition of a saturated aqueous solution of sodium thiosulfate (2 mL) and the mixture was stirred for 5 minutes. A saturated aqueous solution of sodium bicarbonate (0.5 mL) was then added and the resulting mixture was stirred vigorously for 30 minutes. The phases were separated and the aqueous phase was extracted with diethyl ether (3 × 1 mL). The combined organic extracts were washed with brine (3 mL), dried ($MgSO_4$) and concentrated under reduced pressure. The residue was purified by flash column chromatography on silica gel (pentane-ethyl acetate, 1:2) to give 7-*epi*-asbestinin-21 (**16**) (9.1 mg, 87%) as a colourless oil.

(1*R*,2*R*,3*S*,4*S*,5*S*,7*R*,8*S*,11*R*,14*S*,15*R*,17*R*)-14,15-Dihydroxy-5,8,11,15-tetramethyl-10,18-dioxatetracyclo[9.7.0.0^{2,7}.0^{3,17}]octadecan-4-yl acetate (41a), (1*R*,2*R*,3*S*,4*S*,5*S*,7*R*,8*S*,11*R*,14*R*,15*S*,17*R*)-14,15-Dihydroxy-5,8,11,15-tetramethyl-10,18-dioxatetracyclo[9.7.0.0^{2,7}.0^{3,17}]octadecan-4-yl acetate (41b) and 7-*epi*-Asbestinin-21 (16).

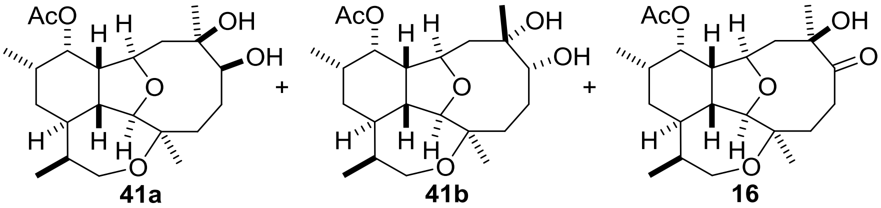

*Dihydroxylation of 11-Acetoxy-4-deoxyasbestinin D (**1**) with the osmium complex of $(DHQD)_2PHAL$*

To a stirred solution of $(DHQD)_2PHAL$ (23 mg, 30 μmol), potassium osmate (7.4 mg, 20 μmol), potassium ferricyanate (1.64 g, 4.98 mmol) and potassium carbonate (690 mg, 4.99 mmol) in a mixture of *t*-BuOH and water (1:1, 1.1 mL) at 0 °C was added 11-acetoxy-4-deoxyasbestinin D (**1**) (18 mg, 50 μmol) in *t*-BuOH (0.3 mL) in one portion. The mixture was warmed to room temperature over 2 hours and then stirred for 16 hours. The reaction was quenched by the addition of a saturated aqueous solution of sodium metabisulfite (3 mL) and the resulting mixture was stirred for 30 minutes. The mixture was diluted with ethyl acetate (5 mL) and the phases were separated. The aqueous phase was extracted with ethyl acetate (3 × 3 mL) and the combined organic extracts were washed with brine (5 mL), dried ($MgSO_4$) and concentrated under reduced pressure. The residue was purified by flash column chromatography on silica gel (pentane-ethyl acetate, gradient elution 1:2 → 1:5) to give the diols **41a** and **41b** (8.0:1, 14.7 mg, 75%) as a colourless solid and 7-*epi*-asbestinin-21 (**16**) (1.9 mg, 10%) as a colourless oil.

*Dihydroxylation of 11-Acetoxy-4-deoxyasbestinin D (**1**) with the osmium complex of $(DHQ)_2PHAL$*

To a stirred solution of $(DHQ)_2PHAL$ (9.1 mg, 12 μmol), potassium osmate (2.9 mg, 7.9 μmol), potassium ferricyanate (642 mg, 1.95 mmol) and potassium carbonate (270 mg, 1.95 mmol) in a mixture of *t*-BuOH and water (1:1, 0.8 mL) at 0 °C was added 11-acetoxy-4-deoxyasbestinin D (**1**) (18 mg, 50 μmol) in *t*-BuOH (0.3 mL) in one portion. The reaction mixture was warmed to room temperature over 2 hours and then stirred for 16 hours. The reaction was quenched by the addition of a saturated aqueous solution of sodium metabisulfite (3 mL) and the resulting mixture was stirred for 30 minutes. The mixture was diluted with ethyl acetate (5 mL) and the phases were separated. The aqueous phase was extracted with ethyl acetate (3 × 3 mL) and the combined organic extracts were washed with brine (5 mL), dried ($MgSO_4$) and concentrated under reduced pressure. The residue was purified by flash column chromatography on silica gel (pentane-ethyl acetate, gradient elution 1:2 → 1:5) to afford the diols **41a** and **41b** (>12:1, 4.6 mg, 23%) as a colourless solid and 7-*epi*-asbestinin-21 (**16**) (9.1 mg, 46%) as a colourless oil.

Asbestinin-23 (20) and 7-*epi*-Asbestinin-23 (43).

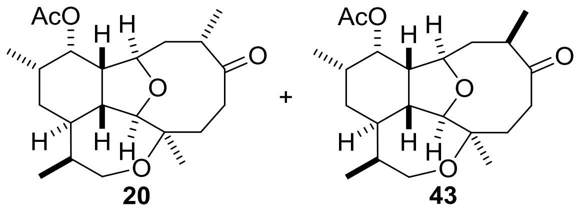

20 **43**

To a stirred solution of 11-acetoxy-4-deoxyasbestinin D (**1**) (26.0 mg, 72 μmol) in anhydrous THF (1.4 mL) at 0 °C was added BH_3•THF (0.2 mL of a 1.0 M solution in THF, 0.2 mmol). The resulting mixture was stirred for 2.5 hours and then the reaction was quenched by the addition of water (2 mL) and sodium perborate tetrahydrate (84 mg, 0.55 mmol). The reaction mixture was warmed to room temperature and stirred for 16 hours. The mixture was diluted with diethyl ether (5 mL) and the phases were separated. The aqueous phase was extracted with diethyl ether (3 × 2 mL) and the combined organic extracts were washed with brine (5 mL), dried ($MgSO_4$) and concentrated under reduced pressure. The resulting crude mixture of alcohols was used in the subsequent reaction without purification.

To a stirred solution of alcohols in anhydrous dichloromethane (1.4 mL) at room temperature was added pyridine (0.30 mL, 0.37 mmol) and Dess-Martin periodinane (73 mg, 0.17 mmol) in one portion. The resulting mixture was stirred for 2 hours and then diluted with diethyl ether (5 mL). The reaction was quenched by the addition of a saturated aqueous solution of sodium thiosulfate (2 mL) and the mixture was stirred for 5 minutes. A saturated aqueous solution of sodium bicarbonate (0.5 mL) was added and the resulting mixture was stirred vigorously for 30 minutes. The phases were separated and the aqueous phase was extracted with diethyl ether (3 × 3 mL). The combined organic extracts were washed with brine (5 mL), dried ($MgSO_4$) and concentrated under reduced pressure. The residue was purified by flash column chromatography on silica gel (pentane-ethyl acetate, gradient elution 10:1 → 2:1) to deliver asbestinin-23 (**20**) (5.3 mg, 20%) and 7-*epi*-asbestinin-23 (**43**) (13.4 mg, 49%) as colourless oils. **20**: R_f = 0.39 (petroleum ether-ethyl acetate, 1:1); $[\alpha]_D^{20}$ −38.3 (*c* = 0.235, $CHCl_3$) {Lit.[4] $[\alpha]_D^{24}$ −22.0 (*c* = 2.30, $CHCl_3$)}; ν_{max} 2959, 2928, 2872, 1736, 1697 cm^{-1}; ^1H NMR (500 MHz, $CDCl_3$) δ 5.25 (1H, dd, *J* = 5.0, 2.8 Hz), 3.94−3.90 (1H, m), 3.80 (1H, d, *J* = 9.5 Hz), 3.78 (1H, br d, *J* = 13.0 Hz), 3.53 (1H, dd, *J* = 13.0, 2.9 Hz), 2.78 (1H, dqd, *J* = 13.4, 6.7, 1.9 Hz), 2.61−2.53 (2H, m), 2.48 (1H, ddd, *J* = 13.4, 12.4, 6.0 Hz), 2.40−2.32 (1H, m), 2.29−2.22 (1H, m), 2.08 (3H, s), 2.07−2.01 (2H, m), 1.96−1.87 (1H, m), 1.73 (1H, dd, *J* = 14.7, 8.0 Hz), 1.65−1.55 (3H, m), 1.30 (3H, s), 1.07 (1H, ddd, *J* = 13.6, 3.5, 1.7 Hz), 1.02 (3H, d, *J* = 6.7 Hz), 0.98 (3H, d, *J* = 7.1 Hz), 0.94 (3H, d, *J* = 7.2 Hz); ^{13}C NMR (126 MHz, $CDCl_3$) δ 171.2, 93.5, 79.8, 77.1, 73.3, 68.5, 48.5, 43.1, 40.9, 39.5, 37.6, 37.4, 36.9, 35.6, 32.0, 31.3, 24.5, 21.4, 18.2, 17.7, 11.2. (peak for ketone not observed); HRMS (ESI+) $[M+Na]^+$ calcd. for $C_{22}H_{34}O_5Na$ 401.2298, found 401.2288 (Δ −2.7 ppm). **43**: R_f = 0.44 (petroleum ether-ethyl acetate, 1:1); $[\alpha]_D^{20}$ −23.6 (*c* = 0.250, $CHCl_3$); ν_{max} 2961, 2932, 2872, 1734, 1694, 993, 951, 936, 733 cm^{-1}; ^1H NMR (400 MHz, $CDCl_3$) δ 5.27 (1H, dd, *J* = 5.0, 2.7 Hz), 3.98 (1H, ddd, *J* = 6.5, 6.5, 3.3 Hz), 3.77 (1H, br d, *J* = 13.2 Hz), 3.74 (1H, d, *J* = 9.4 Hz), 3.49 (1H, dd, *J* = 13.2, 3.0 Hz), 2.87–2.76 (1H, m), 2.57–2.44 (2H, m), 2.42 (1H, ddd, J = 10.6, 10.4, 9.4 Hz), 2.26 (1H, ddd, *J* = 14.8, 10.7, 4.1 Hz), 2.07 (3H, s), 2.06–1.92 (4H, m), 1.85 (1H, dddd, J = 13.7, 10.8, 4.1,

3.7 Hz), 1.73 (1H, ddd, J = 14.8, 7.3, 3.6 Hz,), 1.65–1.58 (1H, m), 1.51 (1H, ddd, J = 13.7, 13.4, 9.7 Hz), 1.33 (3H, s), 1.06 (3H, d, J = 6.9 Hz), 1.01 (1H, ddd, J = 13.4, 3.7, 1.8 Hz), 0.95 (3H, d, J = 7.1 Hz), 0.90 (3H, d, J = 7.2 Hz); ^{13}C NMR (101 MHz, $CDCl_3$) δ 215.2, 171.2, 92.9, 79.3, 73.1, 68.0, 49.9, 44.3, 42.8, 38.8, 38.3, 37.9, 36.7, 34.6, 31.3, 31.2, 24.1, 21.3, 18.0, 17.9, 11.0 (peak at ~77 ppm obscured by solvent); HRMS (ESI+) $[M+Na]^+$ calcd. for $C_{22}H_{34}O_5Na$ 401.2298, found 401.2284 (Δ −3.6 ppm).

(1*R*,2*R*,3*S*,4*S*,5*S*,7*R*,8*S*,11*R*,14*S*,15*R*,17*R*)-14-Hydroxy-5,8,11,15-tetramethyl-10,18-dioxatetracyclo[9.7.0.0^{2,7}.0^{3,17}]octadecan-4-yl acetate (44).

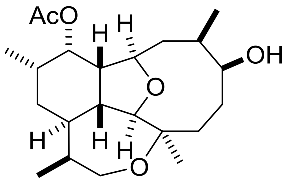

44

To a stirred solution of ketone **43** (12.3 mg, 32.5 μmol) in anhydrous THF (0.6 mL) at −78 °C was added L-selectride (0.04 mL of a 1.0 M solution in THF, 0.04 mmol) dropwise and the resulting solution was stirred for 1 hour. The reaction was quenched by the addition of a water (2 mL) and the mixture was warmed to room temperature and then diluted with ethyl acetate (3 mL). The phases were separated and the aqueous phase was extracted with ethyl acetate (3 × 2 mL). The combined organic extracts were washed with brine (3 mL), dried ($MgSO_4$) and concentrated under reduced pressure. The residue was purified by flash column chromatography on silica gel (pentane-ethyl acetate, gradient elution 2:1 → 1:2) to deliver the alcohol **44** (7.9 mg, 64%) as a colourless solid. R_f = 0.29 (petroleum ether-ethyl acetate, 1:1); m.p. 206−208 °C; $[\alpha]_D^{23}$ +12.4 (c = 0.210, $CHCl_3$); ν_{max} 3451, 2963, 2874, 1736, 974, 930, 731 cm^{-1}; ^{1}H NMR (500 MHz, $CDCl_3$) δ 5.39 (1H, dd, J = 5.7, 1.6 Hz), 4.09 (1H, app dd, J = 12.1, 3.7 Hz), 3.85 (1H, d, J = 8.5 Hz), 3.82−3.74 (2H, m), 3.48 (1H, dd, J = 12.9, 2.3 Hz), 2.59 (1H, ddd, J = 9.9, 9.9, 8.5 Hz), 2.15−1.98 (4H, m), 2.09 (3H, s), 1.93−1.85 (1H, m), 1.84−1.79 (1H, m), 1.77 (1H, dd, J = 9.4, 1.6 Hz), 1.72−1.65 (1H, m), 1.64−1.57 (1H, m), 1.48 (1H, ddd, J = 13.5, 13.5, 9.2 Hz), 1.33 (3H, s), 1.27−1.16 (1H, m), 1.05−0.98 (5H, m), 0.93 (3H, d, J = 7.2 Hz), 0.92 (3H, d, J = 7.2 Hz); ^{13}C NMR (126 MHz, $CDCl_3$) δ 171.5, 94.9, 82.1, 77.8, 73.9, 73.6, 68.1, 48.3, 38.4, 37.9, 37.5, 37.3, 36.5, 31.6, 31.5, 29.9, 28.2, 22.9, 22.7, 21.6, 17.2, 11.1; HRMS (ESI+) $[M+Na]^+$ calcd. for $C_{22}H_{36}O_5Na$ 403.2455, found 403.2446 (Δ −2.1 ppm).

References

1. (a) J. S. Clark, R. Berger, S. T. Hayes, L. H. Thomas, A. J. Morrison, L. Gobbi, *Angew. Chem., Int. Ed.* **2010**, *49*, 9867–9870. (b) J. S. Clark, R. Berger, S. T. Hayes, H. M. Senn, L. J. Farrugia, L. H. Thomas, A. J. Morrison, L. Gobbi, *J. Org. Chem.* **2013**, *78*, 673–696.
2. J. J. Morales, D. Lorenzo, A. Rodríguez, *J. Nat. Prod.* **1991**, *54*, 1368–1382.
3. C. A. Ospina, A. Rodríguez, *J. Nat. Prod.* **2006**, *69*, 1721–1727.
4. A. Rodríguez, O. M. Cóbar, N. Martínez, *J. Nat. Prod.* **1994**, *57*, 1638–1655.
5. A. Rodríguez, O. M. Cóbar, *Tetrahedron* **1993**, *49*, 319–328.

^{1}H and ^{13}C NMR Spectra for New Compounds

	Page
^{1}H NMR Spectrum of **29** ($CDCl_3$)	35
^{13}C NMR Spectrum of **29** ($CDCl_3$)	36
^{1}H NMR Spectrum of **30** ($CDCl_3$)	37
^{13}C NMR Spectrum of **30** ($CDCl_3$)	38
^{1}H NMR Spectrum of **S1** ($CDCl_3$)	39
^{13}C NMR Spectrum of **S1** ($CDCl_3$)	40
^{1}H NMR Spectrum of **31** ($CDCl_3$)	41
^{13}C NMR Spectrum of **31** ($CDCl_3$)	42
HSQC NMR Spectrum of **31** ($CDCl_3$)	43
^{1}H NMR Spectrum of **32** ($CDCl_3$)	44
^{13}C NMR Spectrum of **32** ($CDCl_3$)	45
^{1}H NMR Spectrum of **33** (C_6D_6)	46
^{13}C NMR Spectrum of **33** (C_6D_6)	47
^{1}H NMR Spectrum of **34** ($CDCl_3$)	48
^{13}C NMR Spectrum of **34** ($CDCl_3$)	49
^{1}H NMR Spectrum of **36** ($CDCl_3$)	50
^{13}C NMR Spectrum of **36** ($CDCl_3$)	51
^{1}H NMR Spectrum of **37** ($CDCl_3$)	52
^{13}C NMR Spectrum of **37** ($CDCl_3$)	53
^{1}H NMR Spectrum of **38a** ($CDCl_3$)	54
^{13}C NMR Spectrum of **38a** ($CDCl_3$)	55
^{1}H NMR Spectrum of **38b** ($CDCl_3$)	56
^{13}C NMR Spectrum of **38b** ($CDCl_3$)	57
^{1}H NMR Spectrum of **39** ($CDCl_3$)	58
^{13}C NMR Spectrum of **39** ($CDCl_3$)	59
^{1}H NMR Spectrum of **1** ($CDCl_3$)	60
^{13}C NMR Spectrum of **1** ($CDCl_3$)	61
^{1}H NMR Spectrum of **2** ($CDCl_3$)	62
^{13}C NMR Spectrum of **2** ($CDCl_3$)	63
^{1}H NMR Spectrum of **40a** ($CDCl_3$)	64
^{13}C NMR Spectrum of **40a** ($CDCl_3$)	65
^{1}H NMR Spectrum of **40b** ($CDCl_3$)	66
^{13}C NMR Spectrum of **40b** ($CDCl_3$)	67

^{1}H NMR Spectrum of **6** ($CDCl_3$) 68
^{13}C NMR Spectrum of **6** ($CDCl_3$) 69
HMBC NMR Spectrum of **6** ($CDCl_3$) 70
^{1}H NMR Spectrum of **9** ($CDCl_3$) 71
^{13}C NMR Spectrum of **9** ($CDCl_3$) 72
^{1}H NMR Spectrum of **14** ($CDCl_3$) 73
^{13}C NMR Spectrum of **14** ($CDCl_3$) 74
^{1}H NMR Spectrum of **14** (C_6D_6) 75
^{13}C NMR Spectrum of **14** (C_6D_6) 76
^{1}H NMR Spectrum of **41a** ($CDCl_3$) 77
^{13}C NMR Spectrum of **41a** ($CDCl_3$) 78
HSQC NMR Spectrum of **41a** ($CDCl_3$) 79
HMBC NMR Spectrum of **41a** ($CDCl_3$) 80
^{1}H NMR Spectrum of **42** ($CDCl_3$) 81
^{13}C NMR Spectrum of **42** ($CDCl_3$) 82
HSQC NMR Spectrum of **42** ($CDCl_3$) 83
HMBC NMR Spectrum of **42** ($CDCl_3$) 84
^{1}H NMR Spectrum of **16** ($CDCl_3$) 85
^{13}C NMR Spectrum of **16** ($CDCl_3$) 86
^{1}H NMR Spectrum of **20** ($CDCl_3$) 87
^{13}C NMR Spectrum of **20** ($CDCl_3$) 88
HMBC NMR Spectrum of **20** ($CDCl_3$) 89
^{1}H NMR Spectrum of **43** ($CDCl_3$) 90
^{13}C NMR Spectrum of **43** ($CDCl_3$) 91
^{1}H NMR Spectrum of **44** ($CDCl_3$) 92
^{13}C NMR Spectrum of **44** ($CDCl_3$) 93

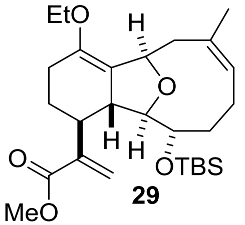

29

(400 MHz, $CDCl_3$)

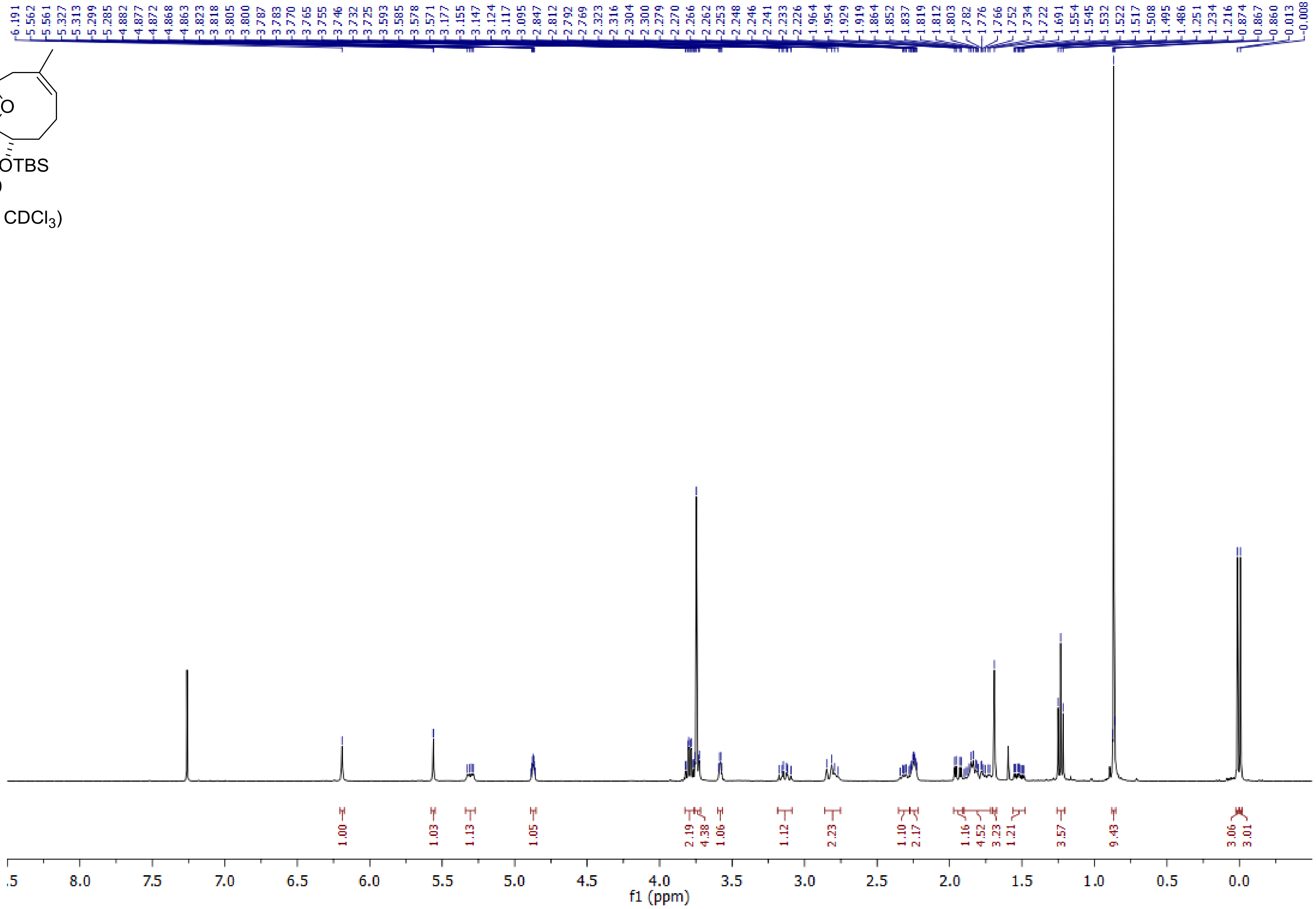

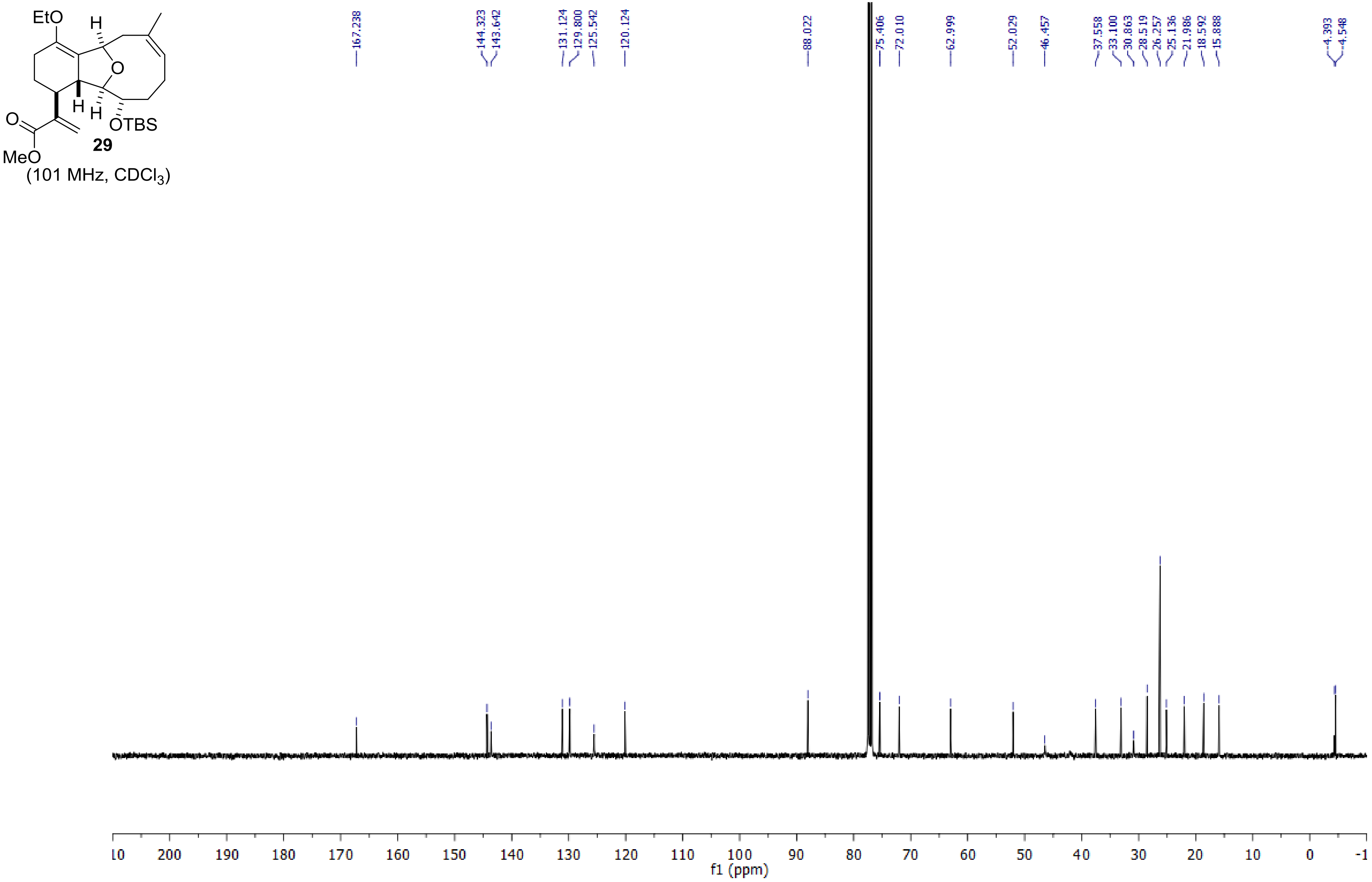

EtO
H
O
H
H
OTBS
O
MeO
29
(101 MHz, CDCl3)
167.238
144.323
143.642
131.124
129.800
125.542
120.124
88.022
75.406
72.010
62.999
52.029
46.457
37.558
33.100
30.863
28.519
26.257
25.136
21.986
18.592
15.888
-4.393
-4.548
f1 (ppm)

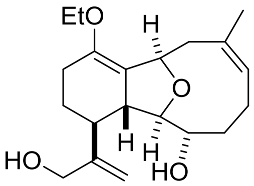

30

(400 MHz, C_6D_6)

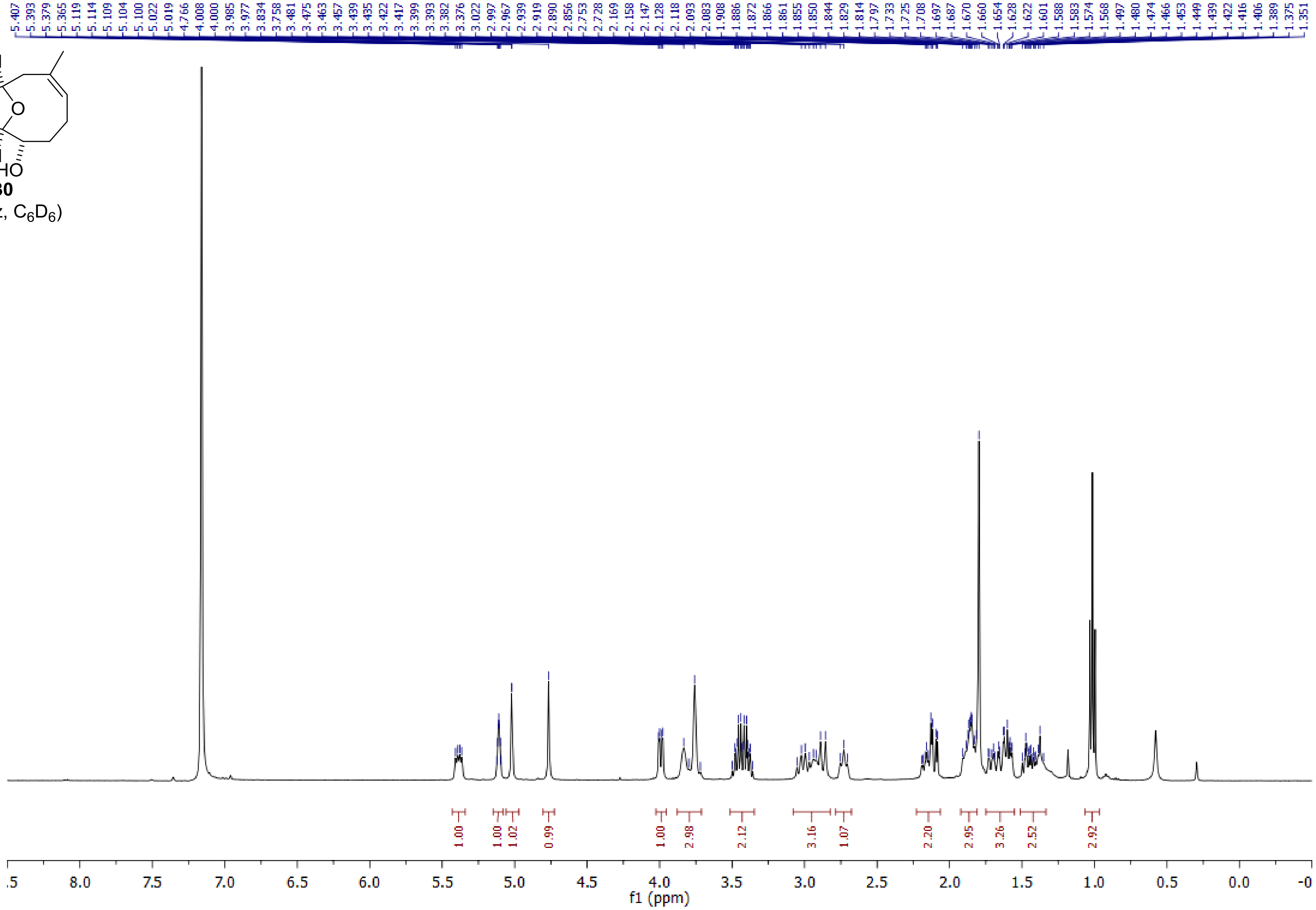

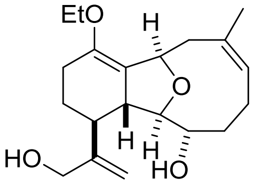

30

(101 MHz, C_6D_6)

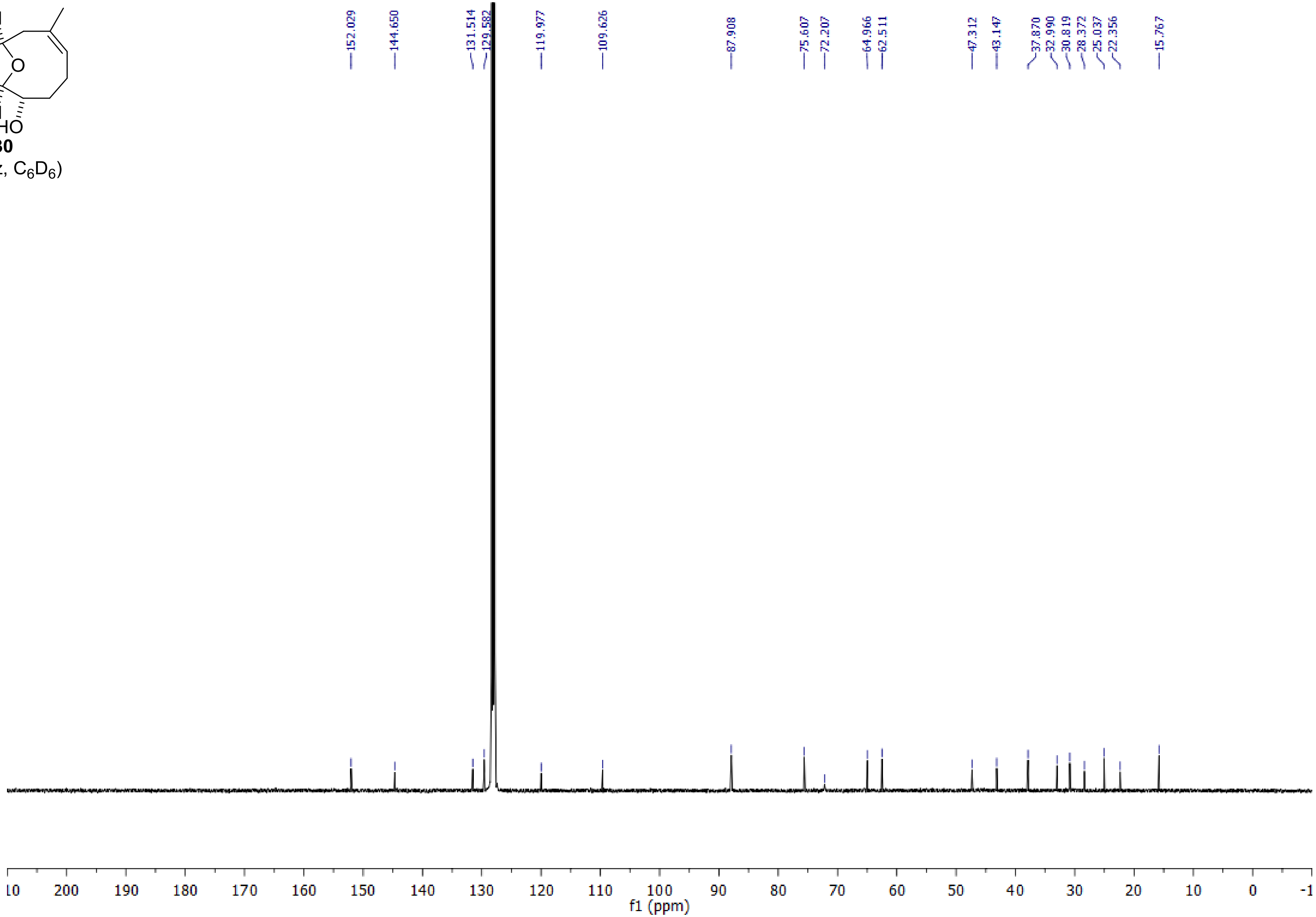

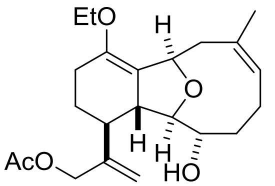

S1
(400 MHz, $CDCl_3$)

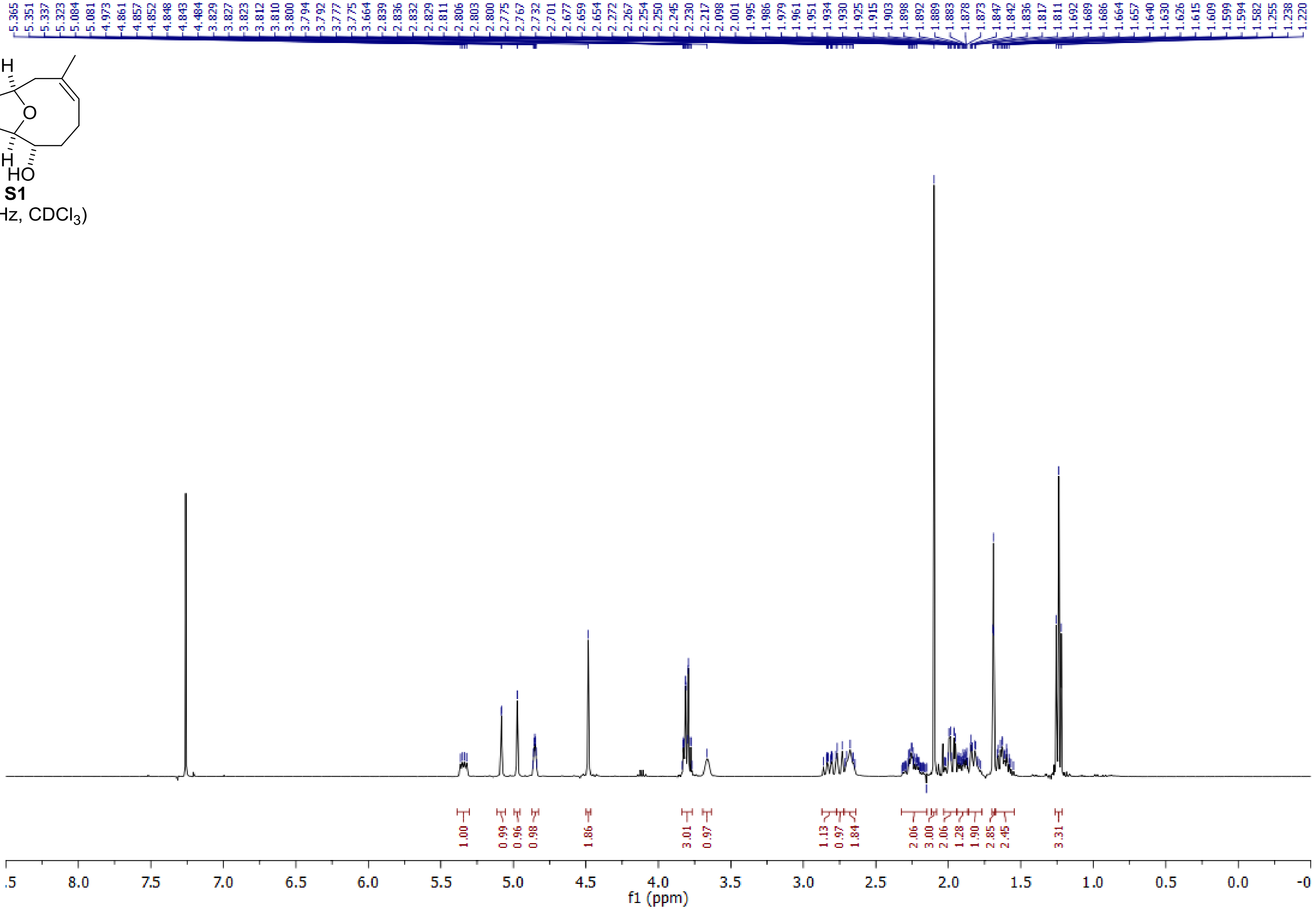

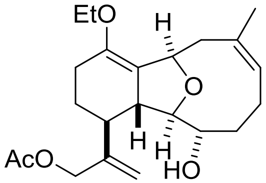

S1

(101 MHz, $CDCl_3$)

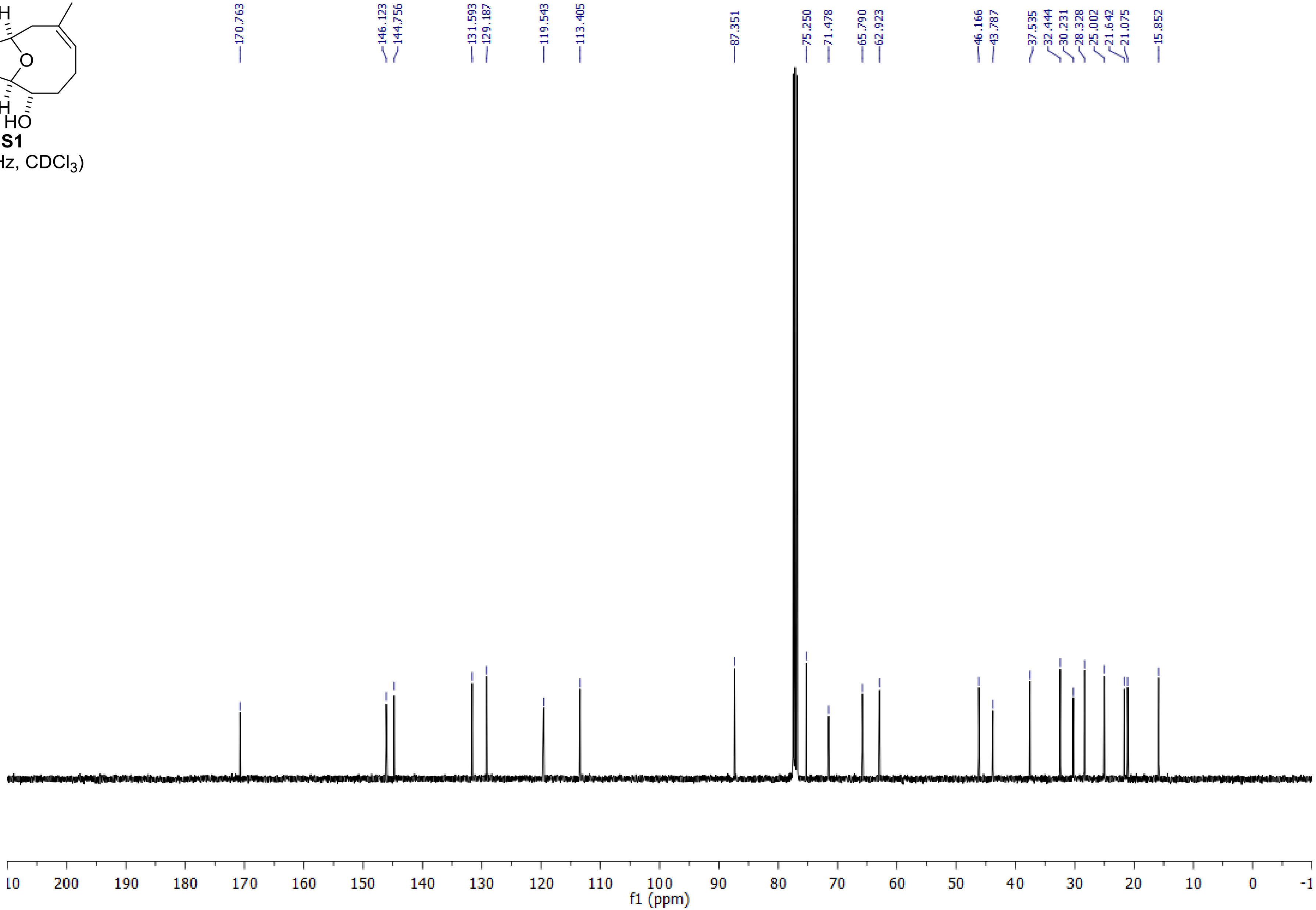

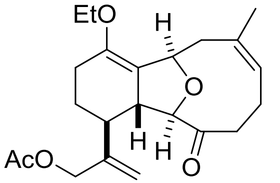

31
(400 MHz, $CDCl_3$)

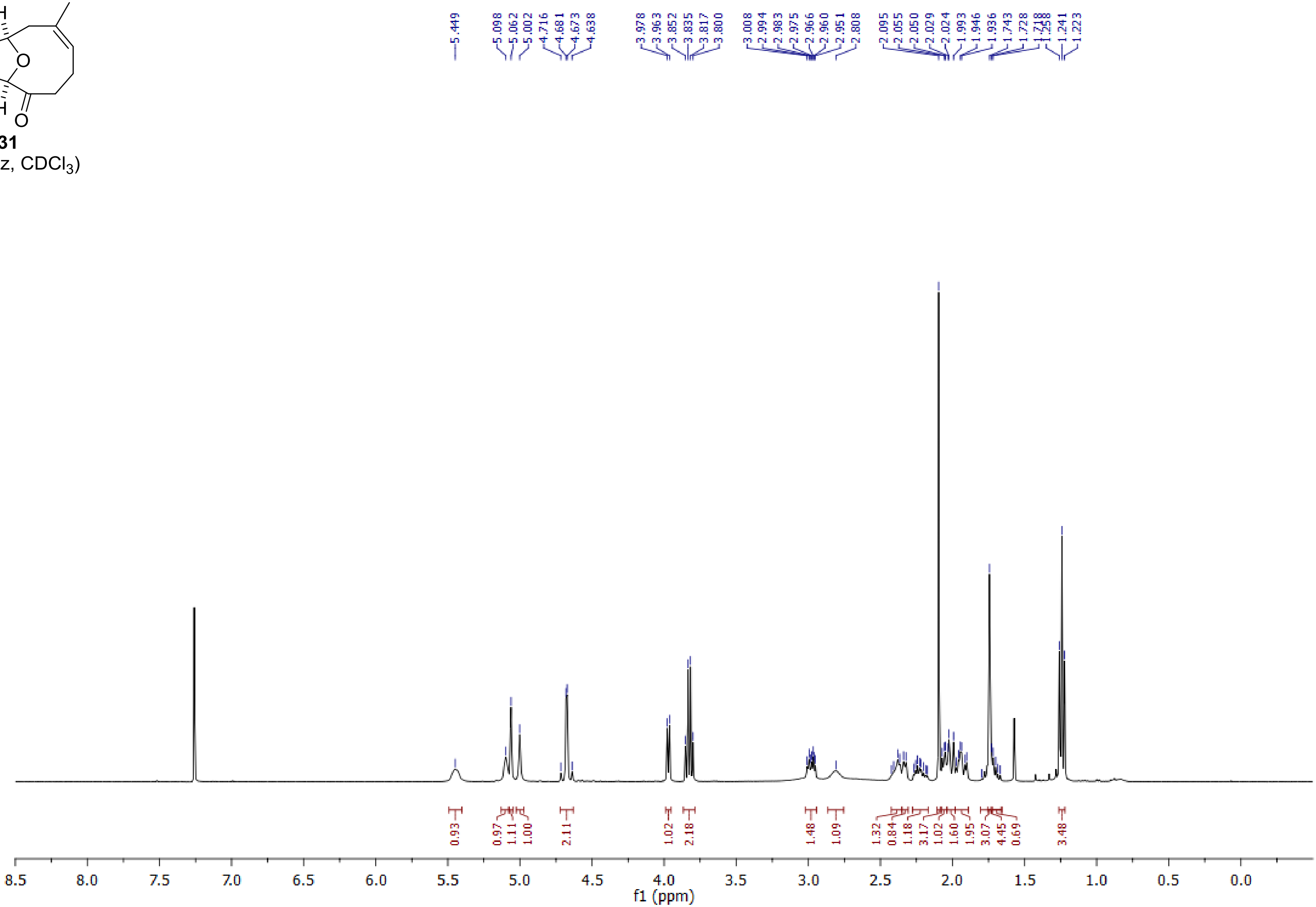

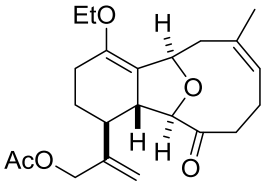

31

(101 MHz, $CDCl_3$)

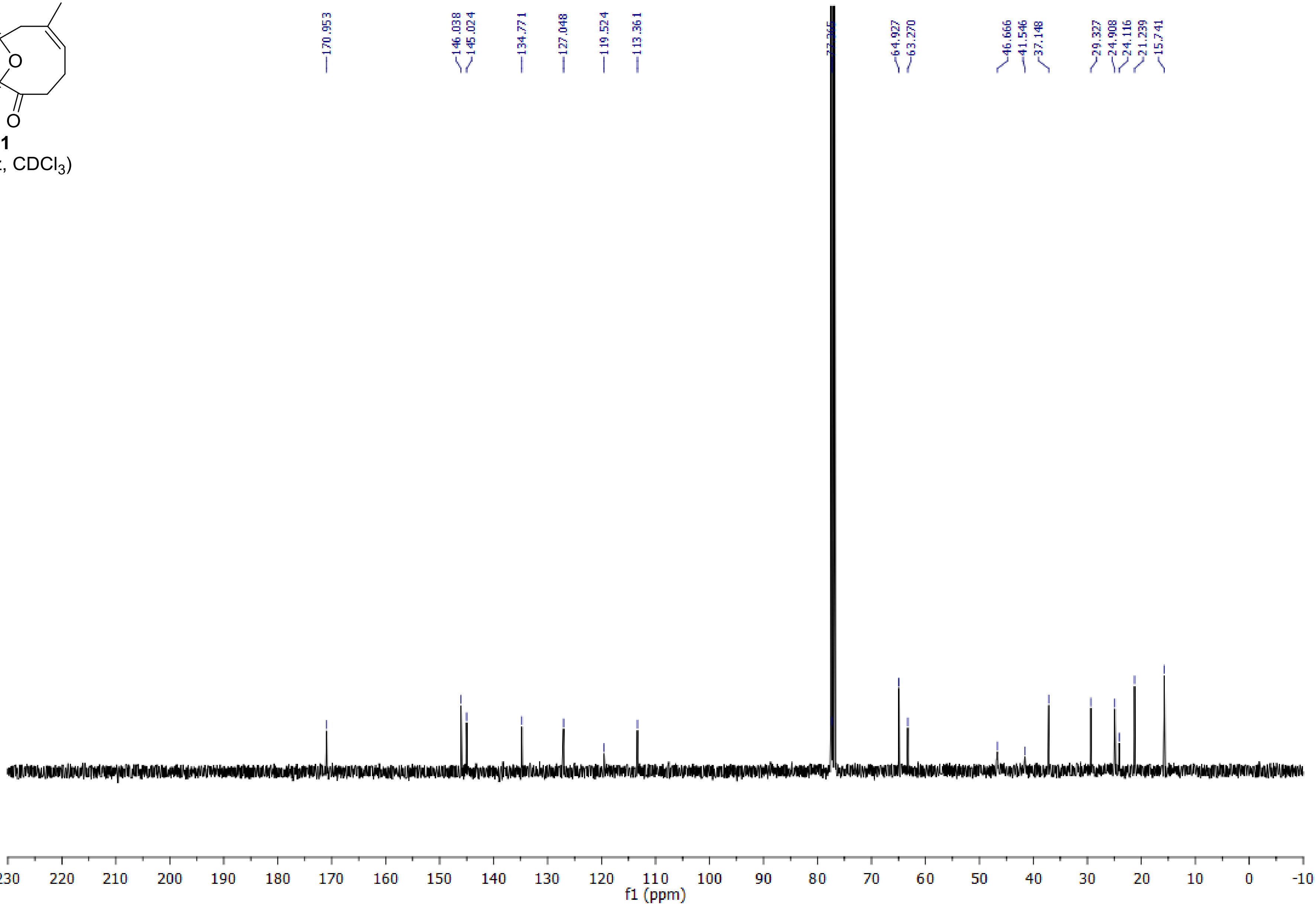

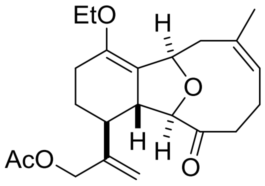

31

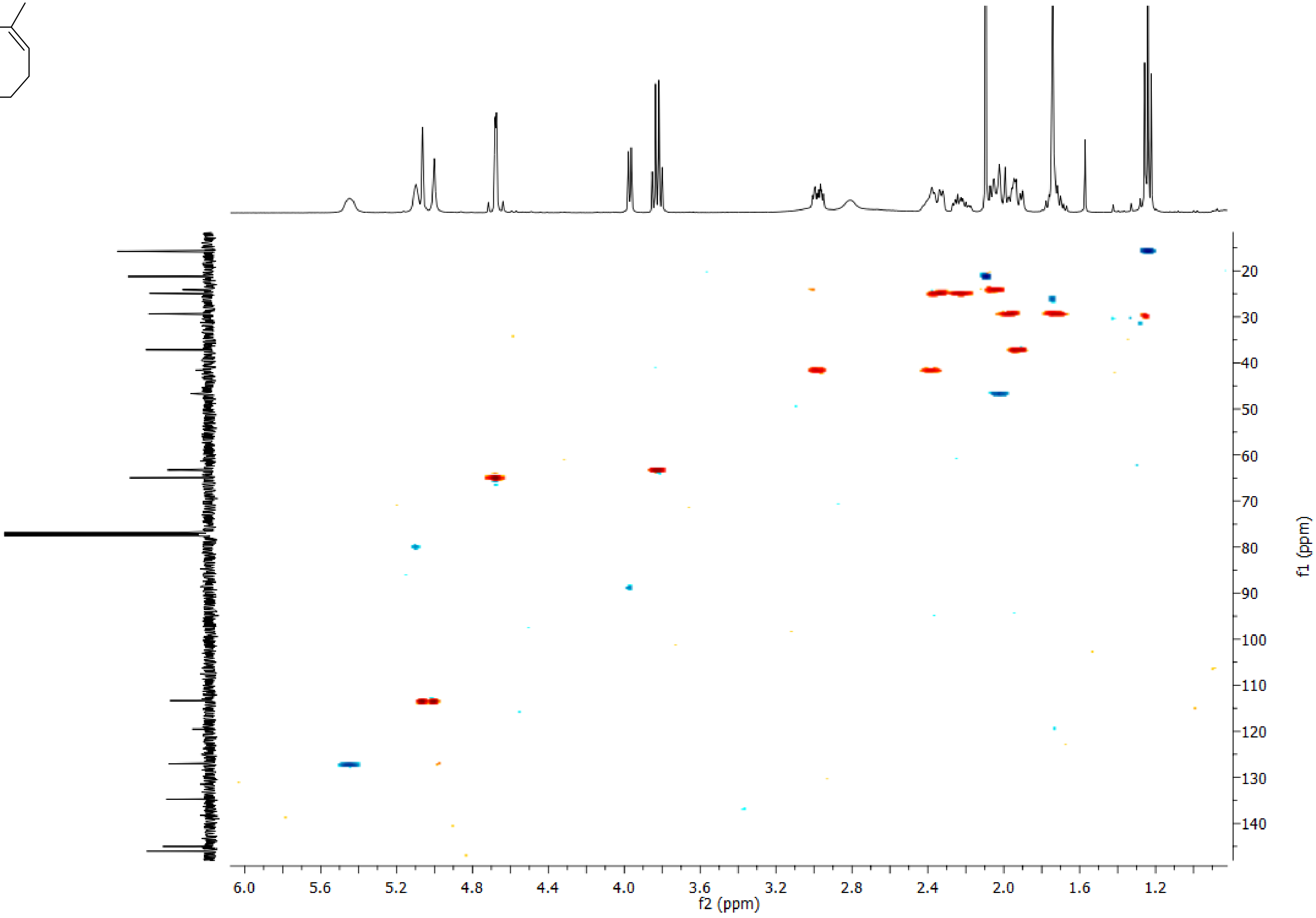

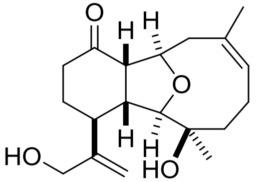

32

(400 MHz, $CDCl_3$)

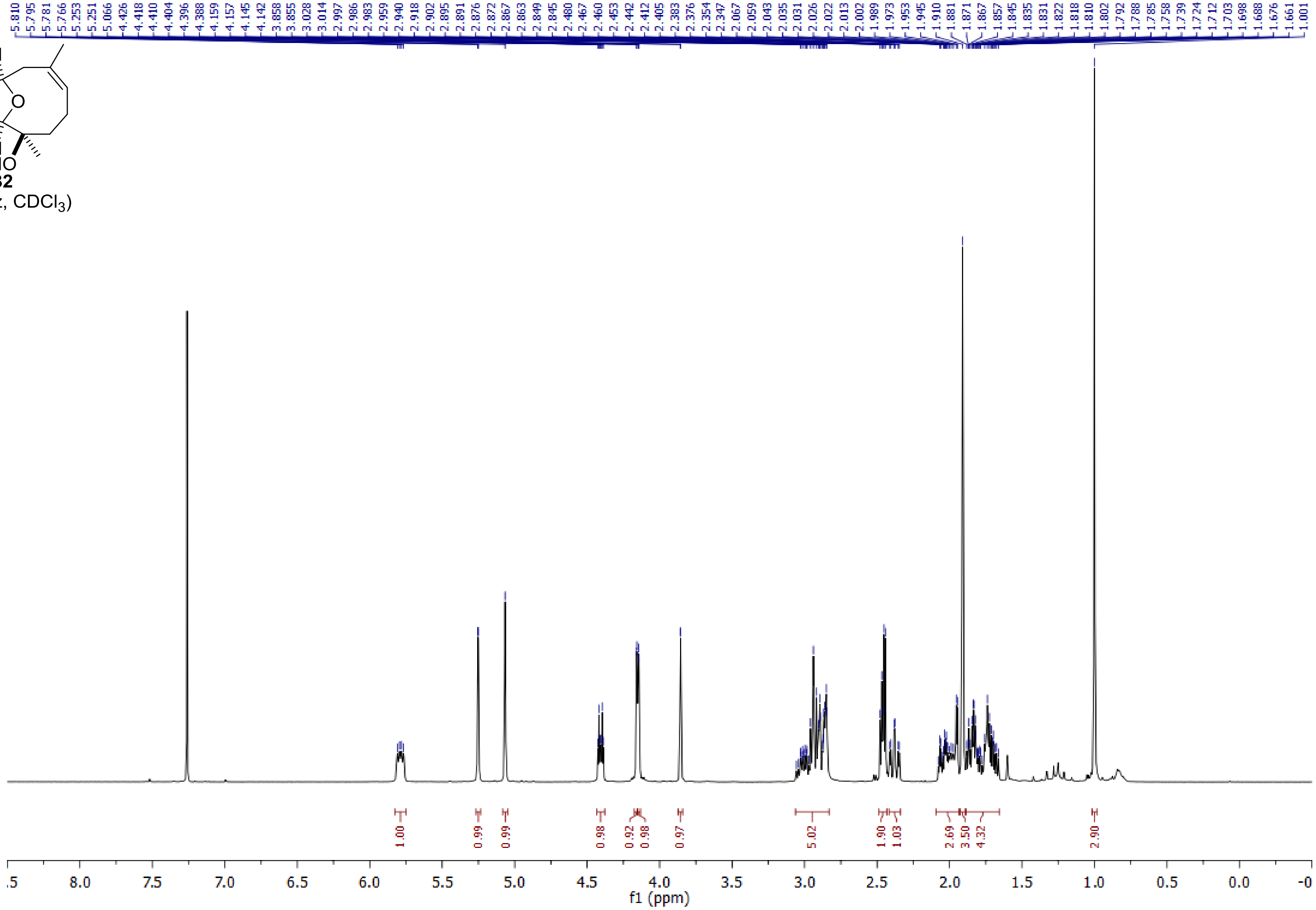

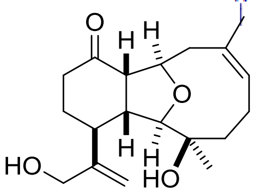

32
(101 MHz, $CDCl_3$)

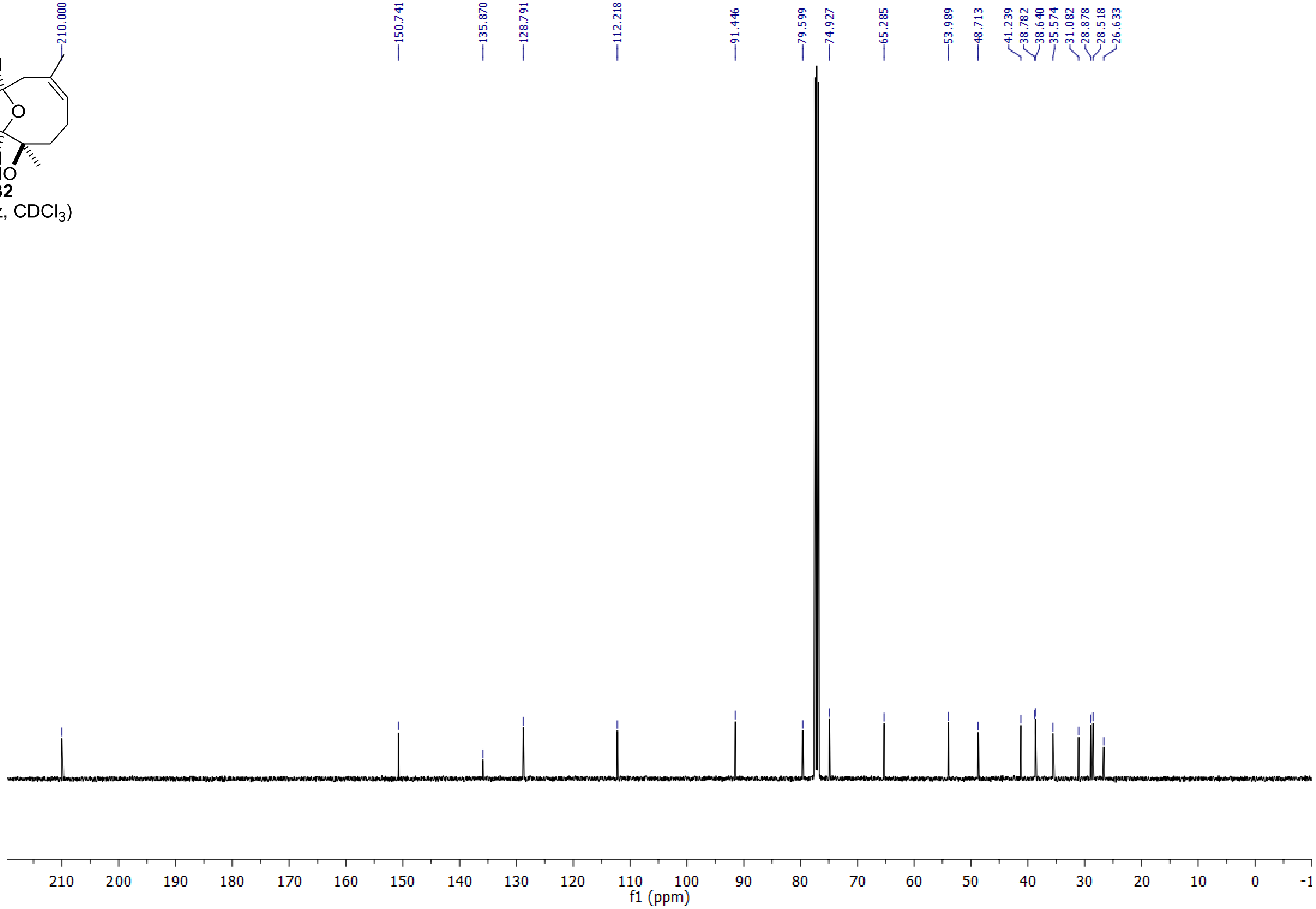

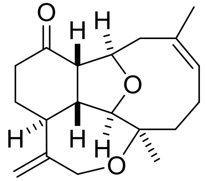

33

(400 MHz, C_6D_6)

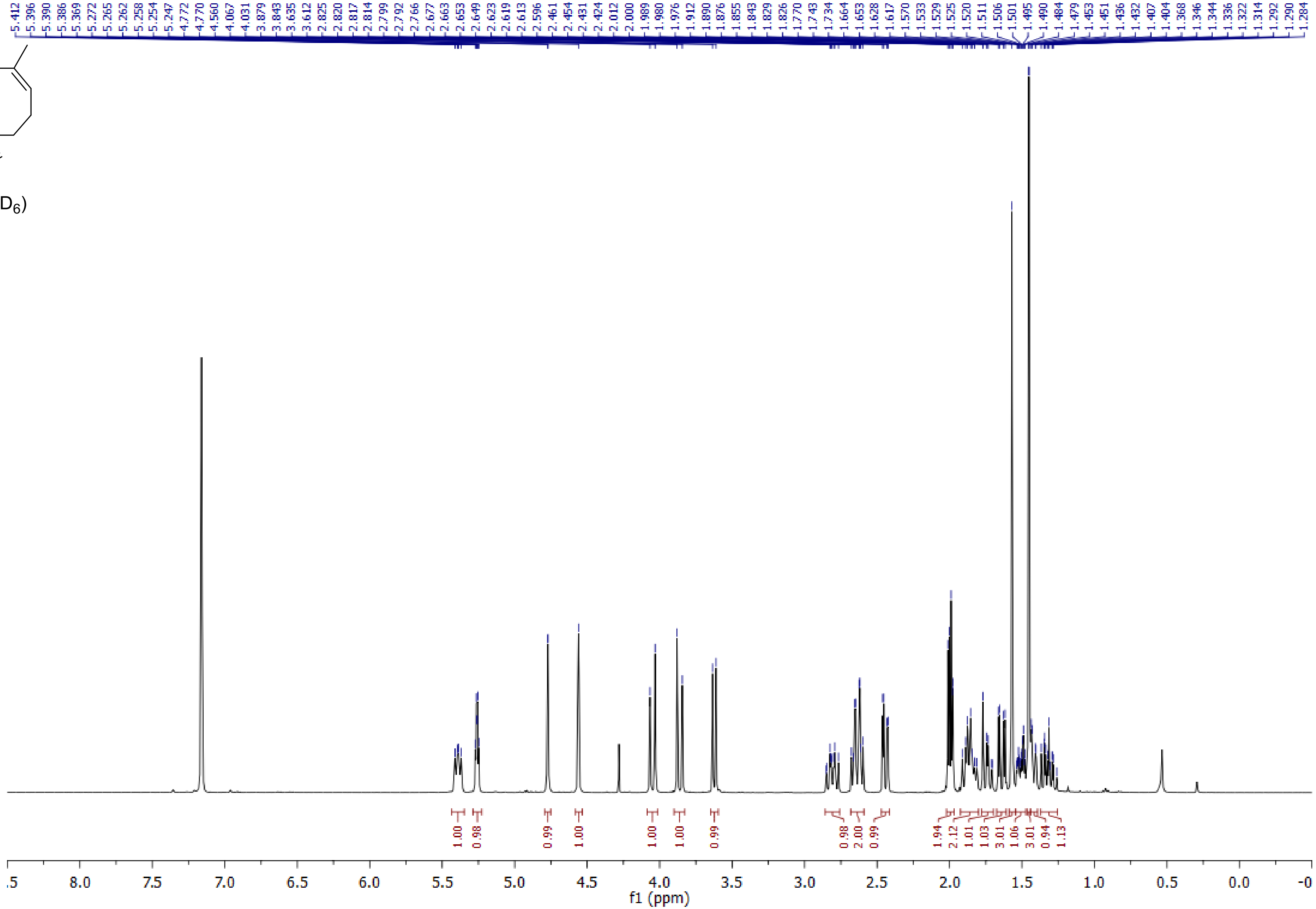

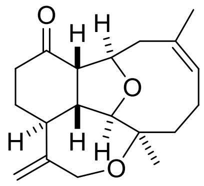

33

(101 MHz, C_6D_6)

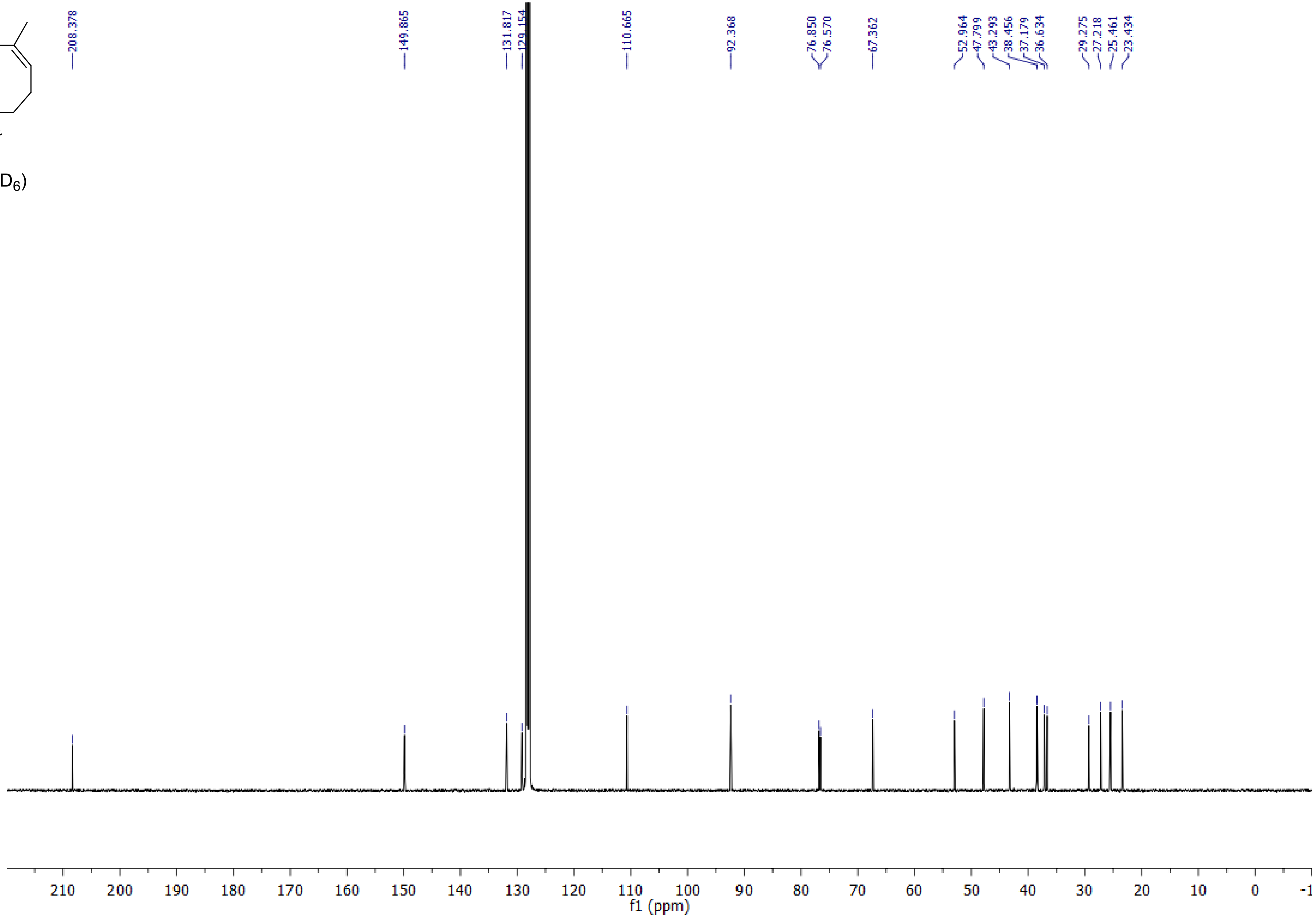

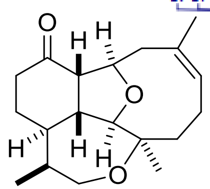

34
(400 MHz, $CDCl_3$)

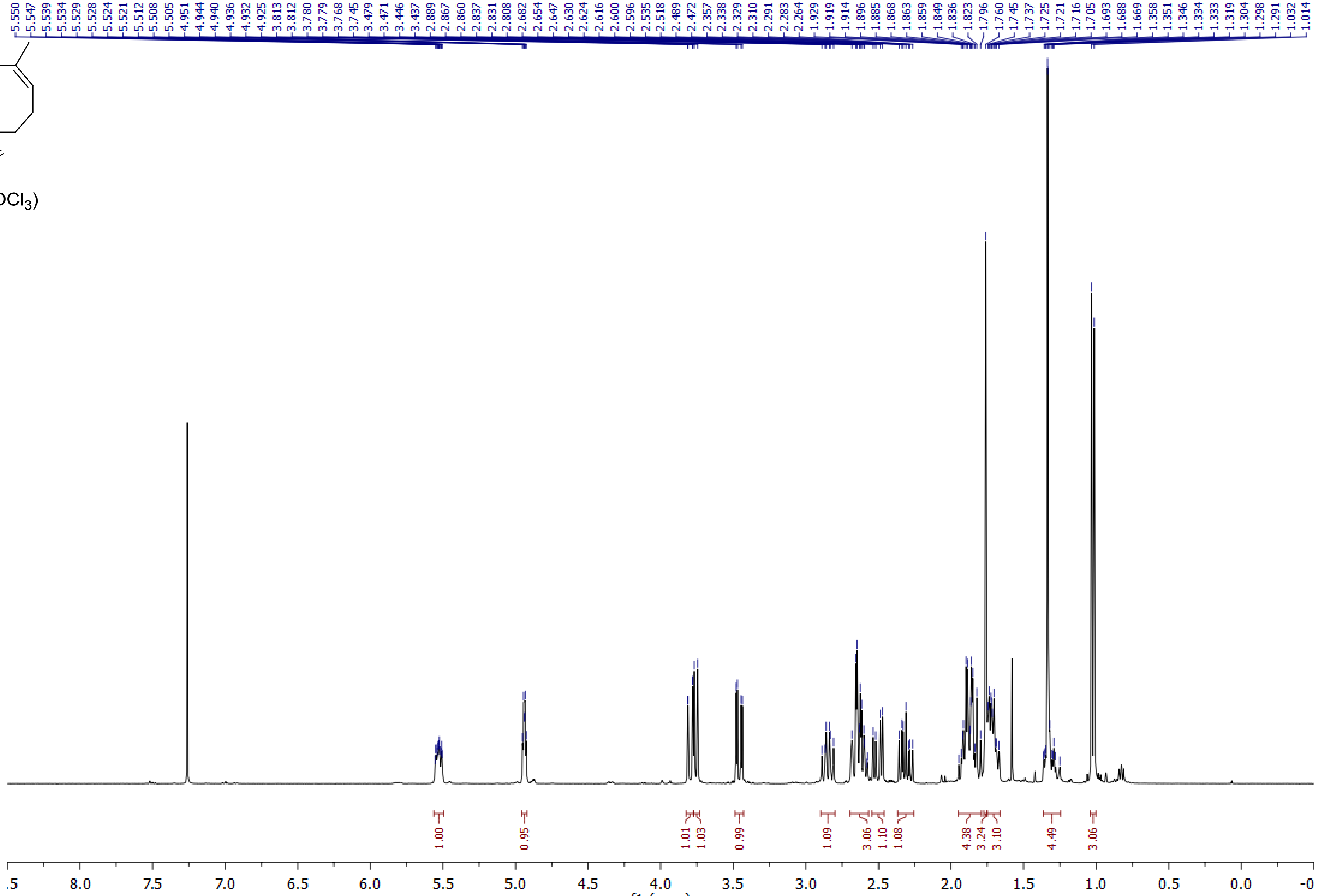

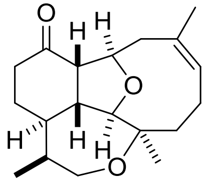

34

(101 MHz, $CDCl_3$)

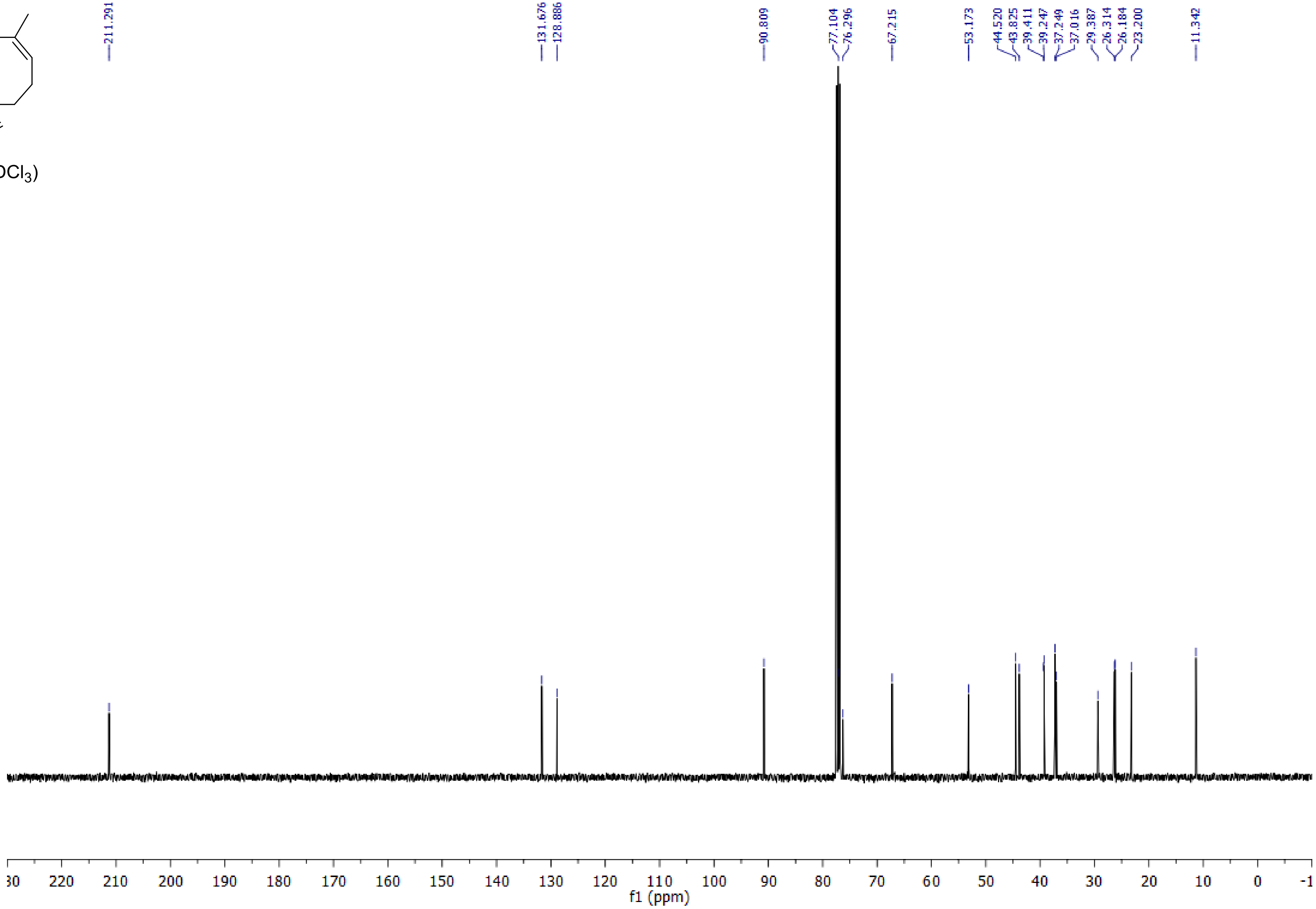

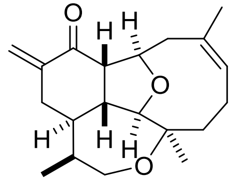

36

(400 MHz, $CDCl_3$)

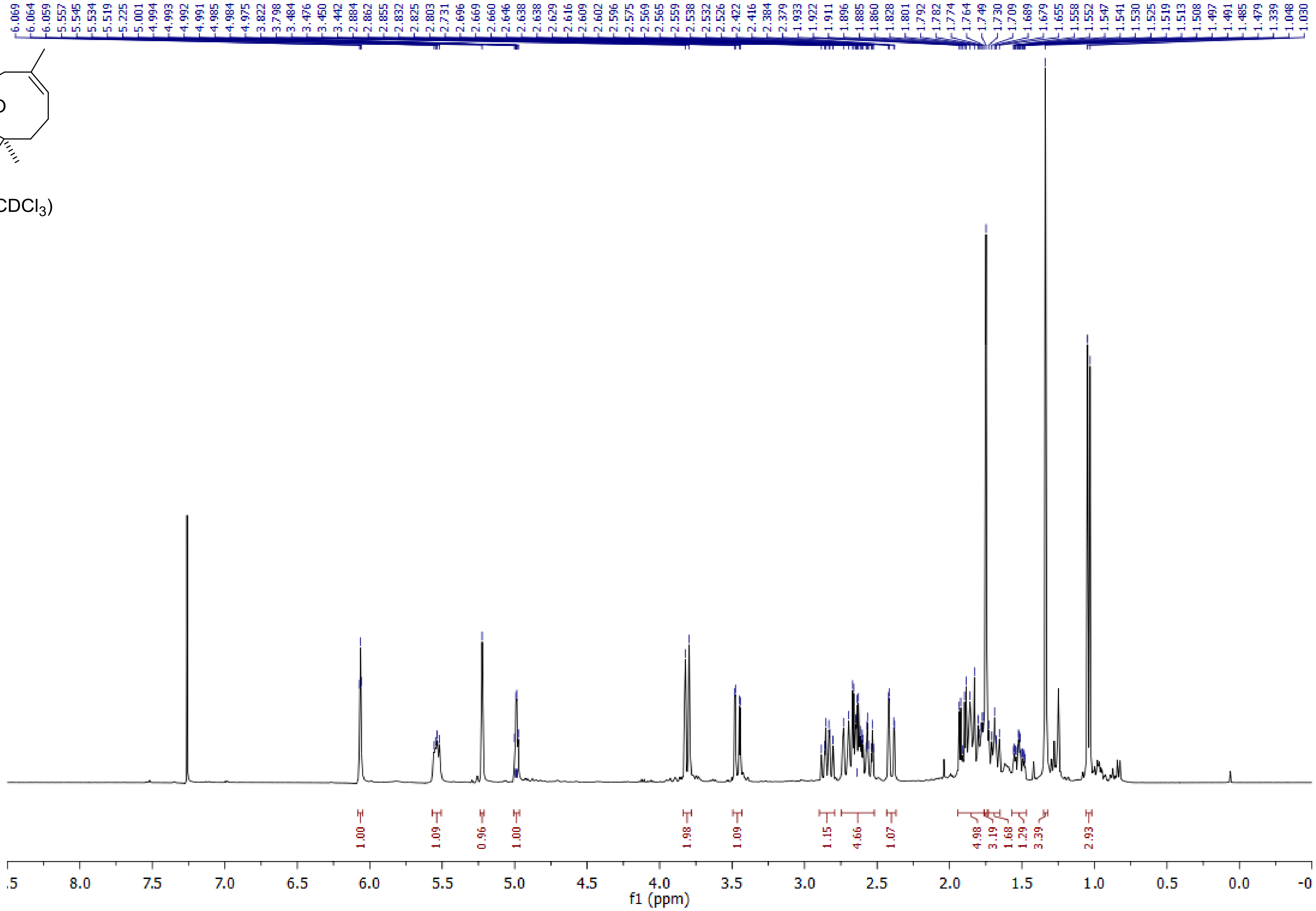

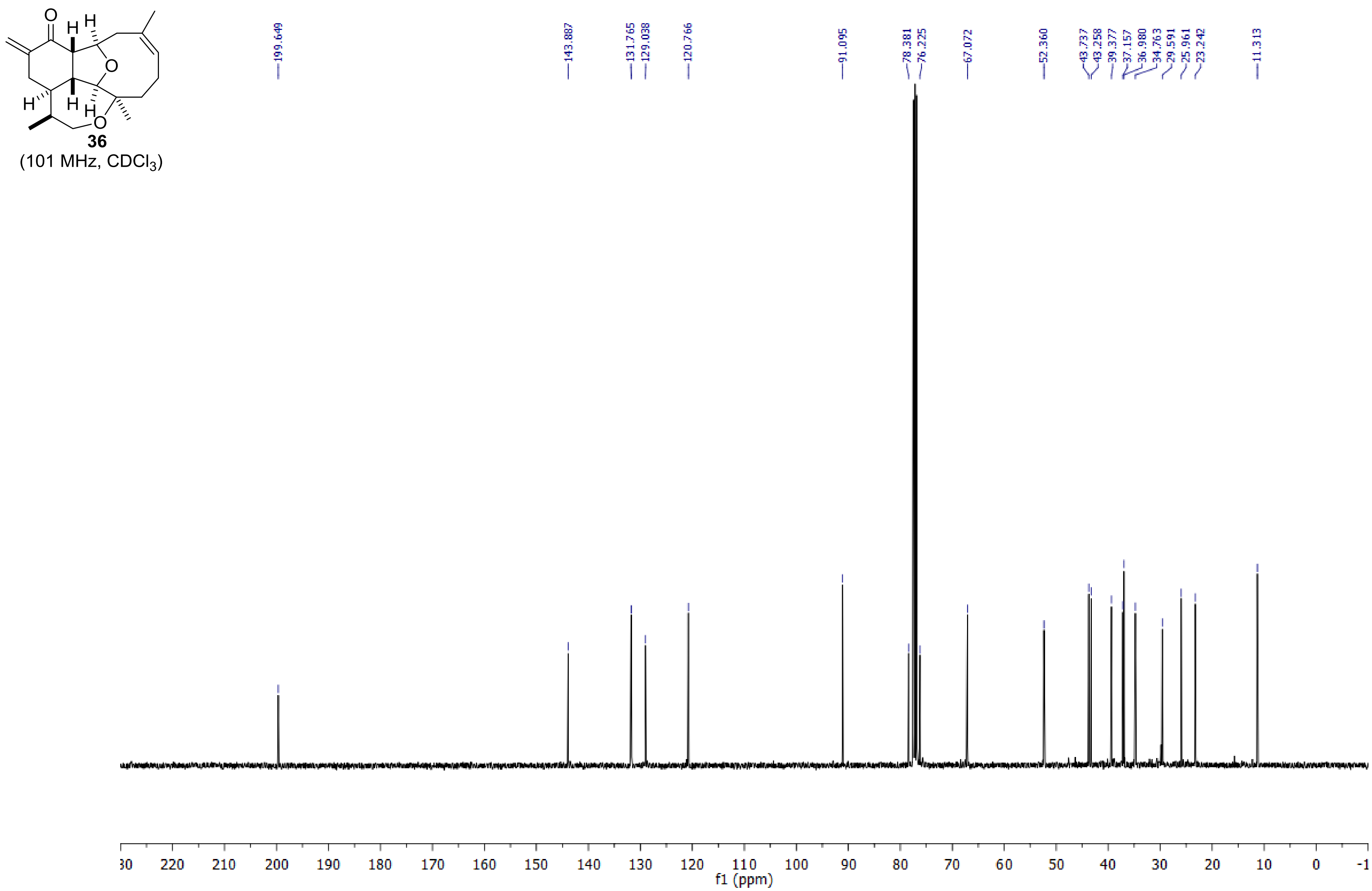
36
(101 MHz, CDCl3)
199.649
143.887
131.765
129.038
120.766
91.095
78.381
76.225
67.072
52.360
43.737
43.258
39.377
37.157
36.980
34.763
29.591
25.961
23.242
11.313
f1 (ppm)

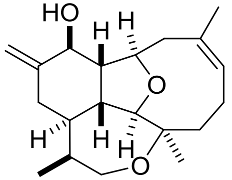

37
(400 MHz, $CDCl_3$)

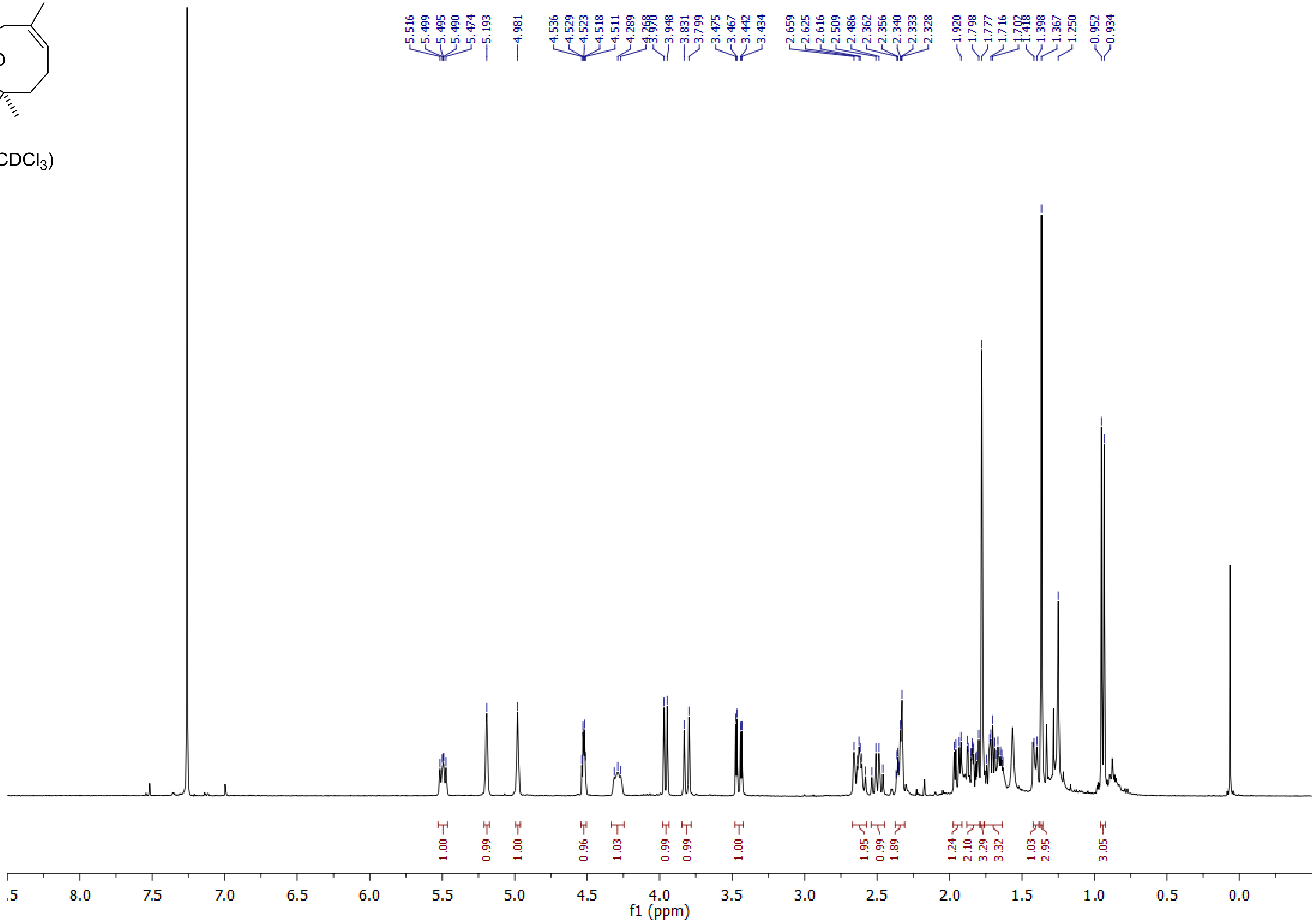

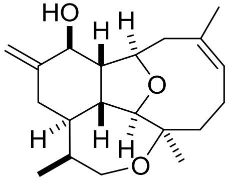

37

(101 MHz, $CDCl_3$)

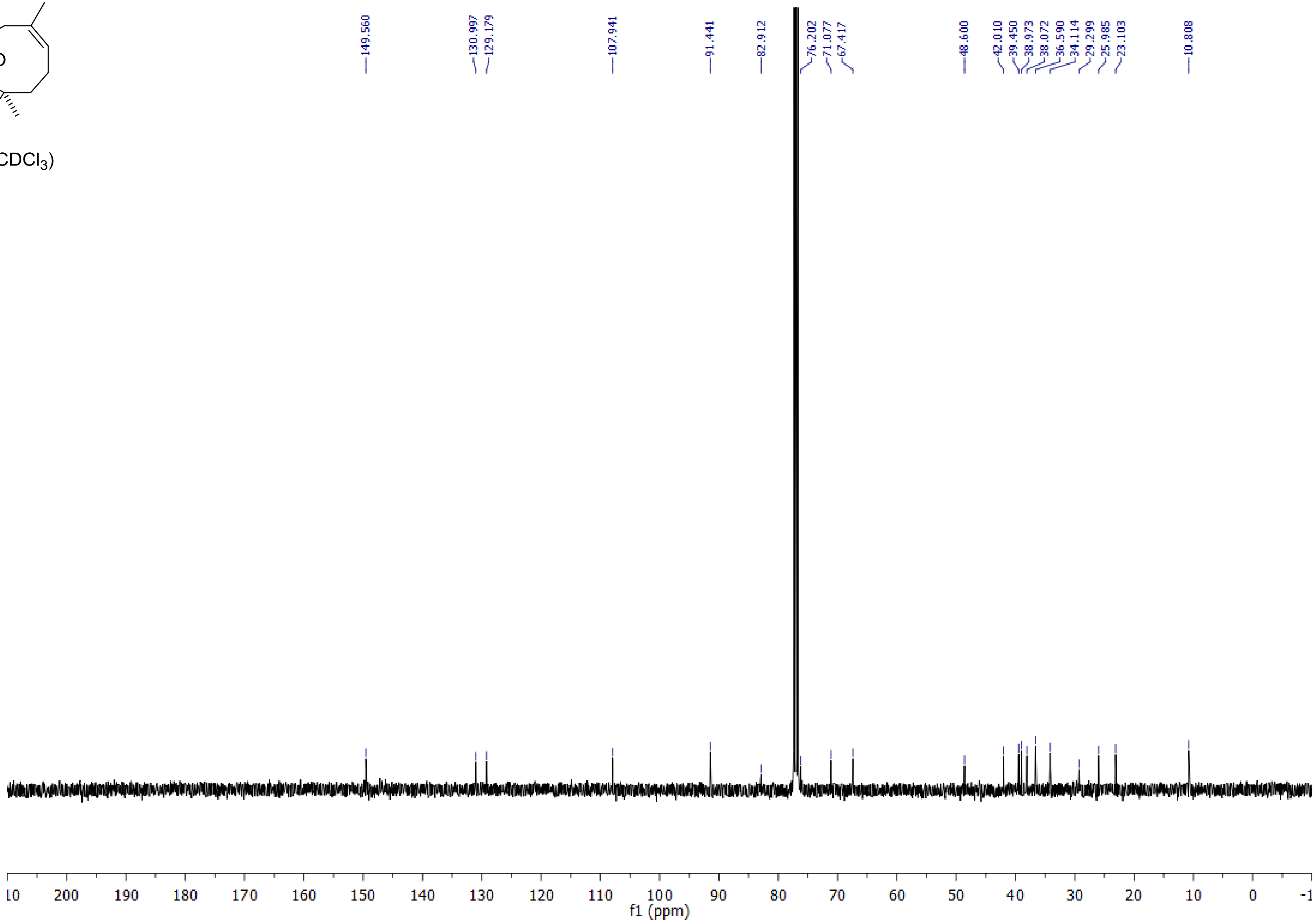

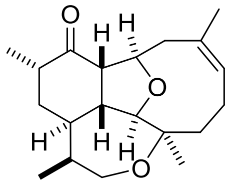

38a
(400 MHz, $CDCl_3$)

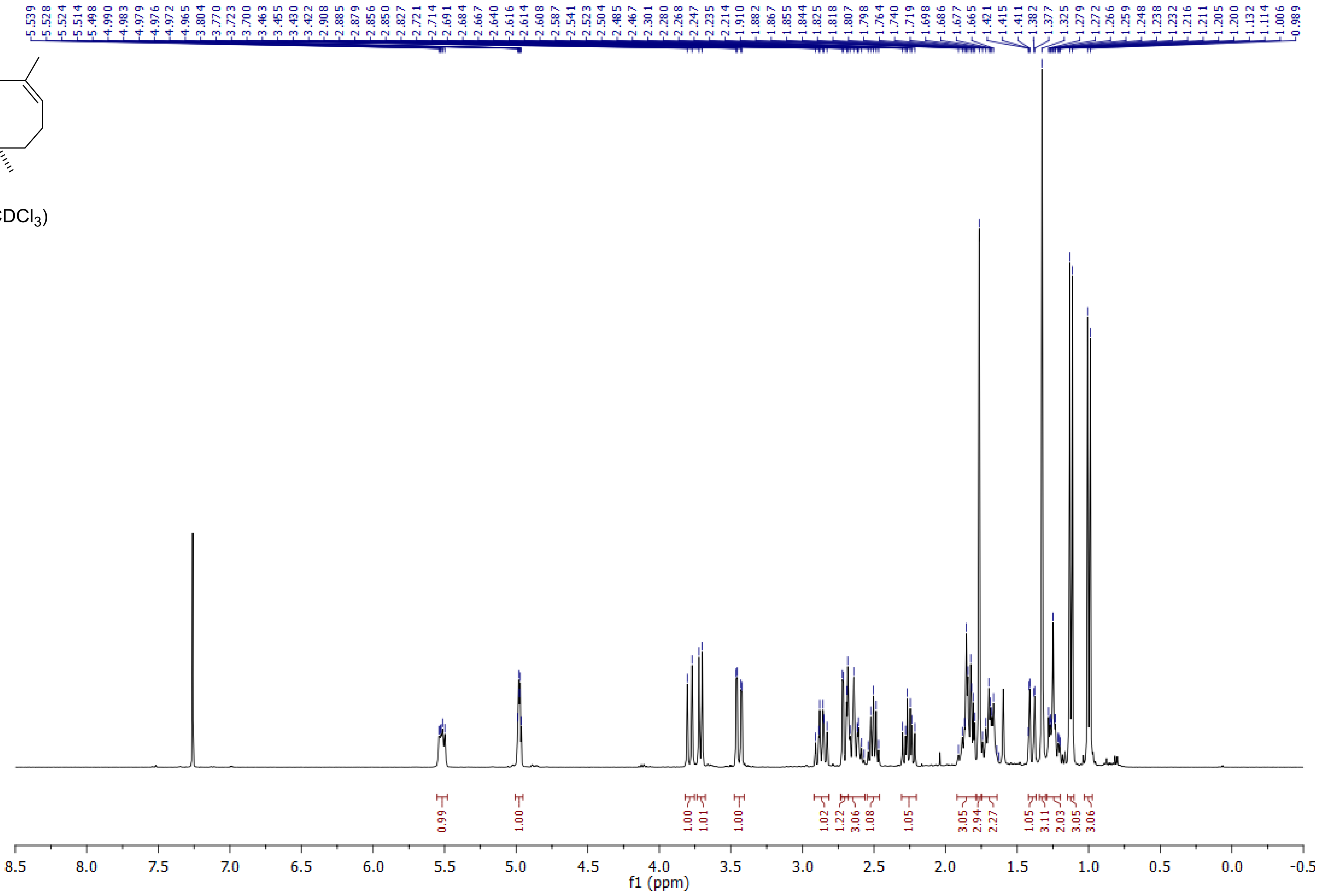

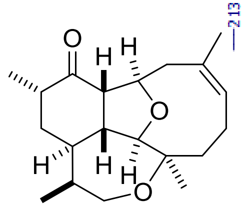

38a
(101 MHz, $CDCl_3$)

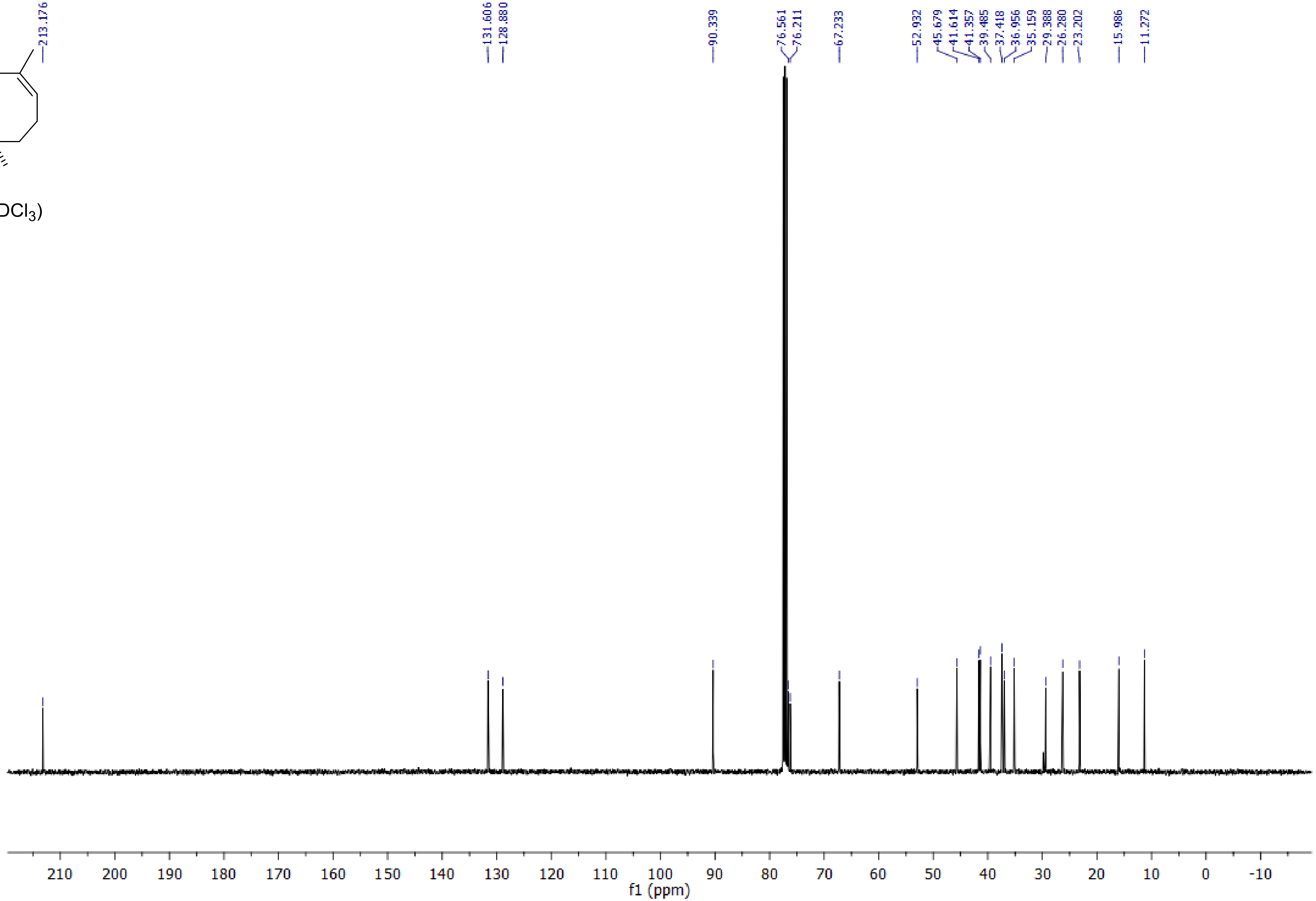

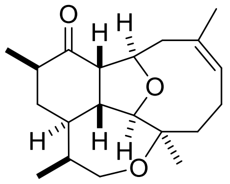

38b

(400 MHz, $CDCl_3$)

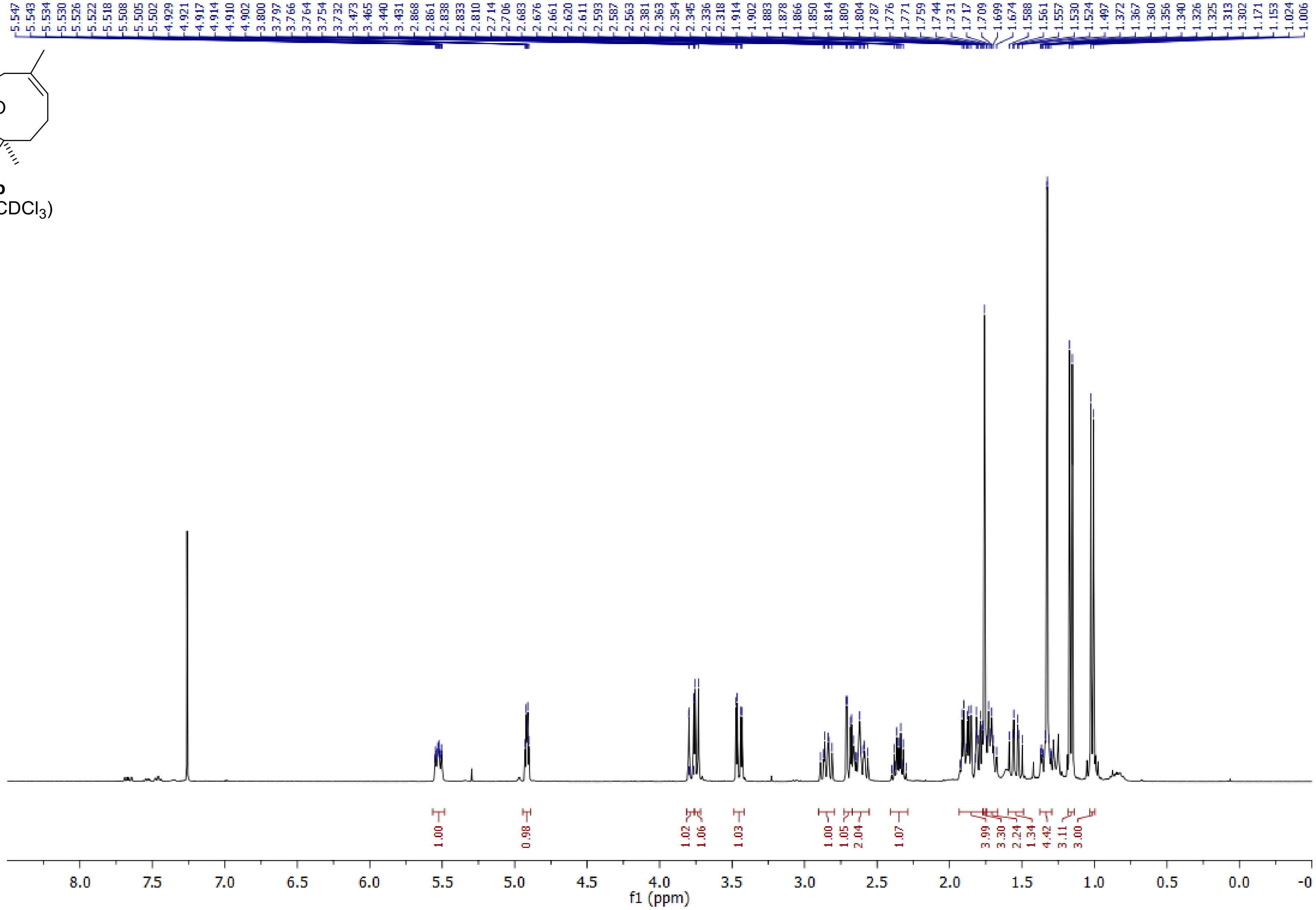

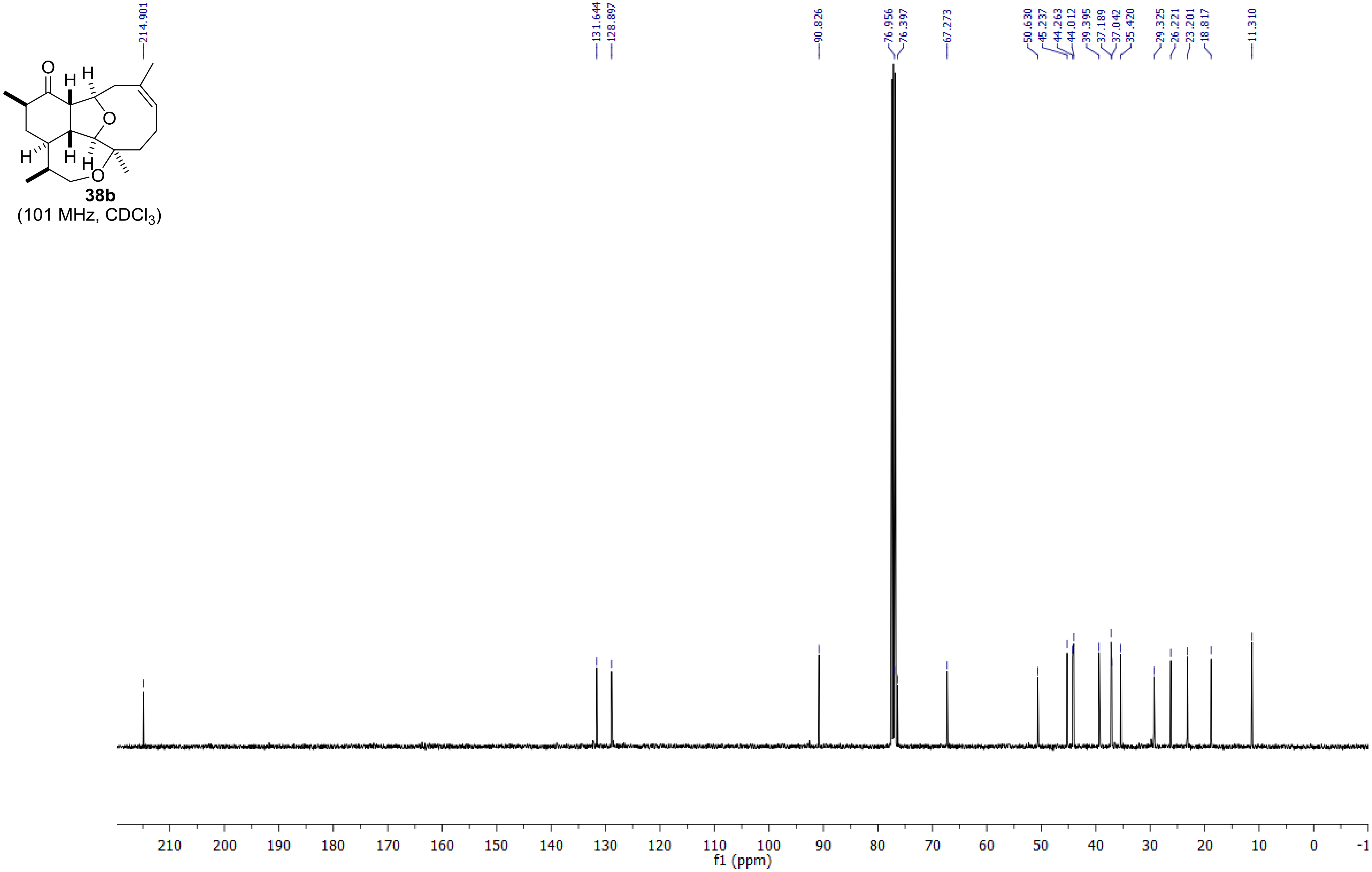

38b
(101 MHz, $CDCl_3$)

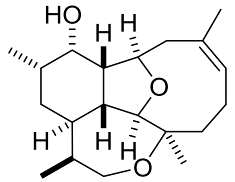

39

(400 MHz, $CDCl_3$)

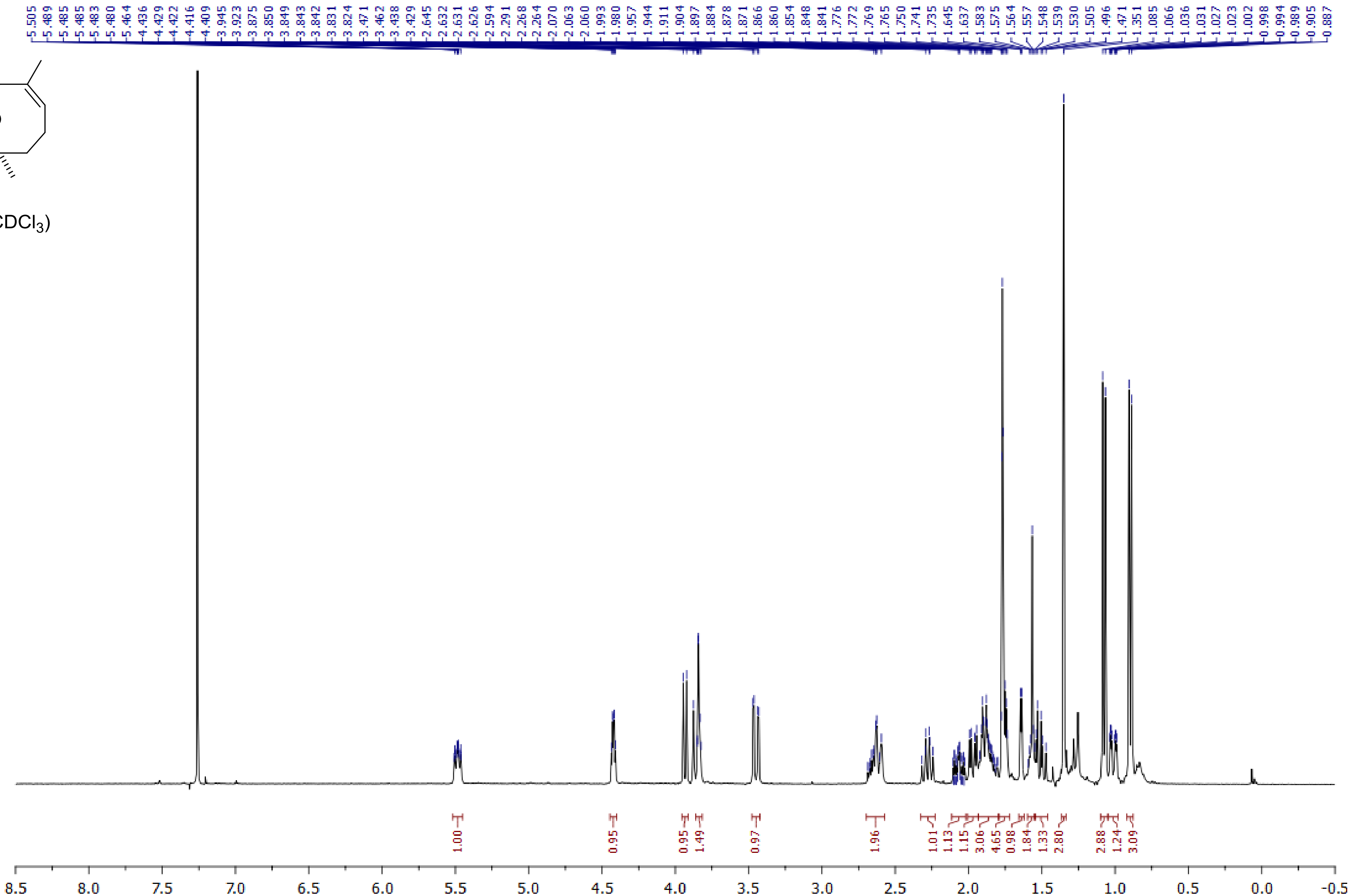

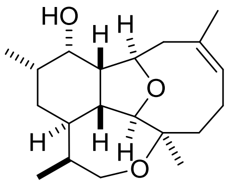

39
(101 MHz, $CDCl_3$)

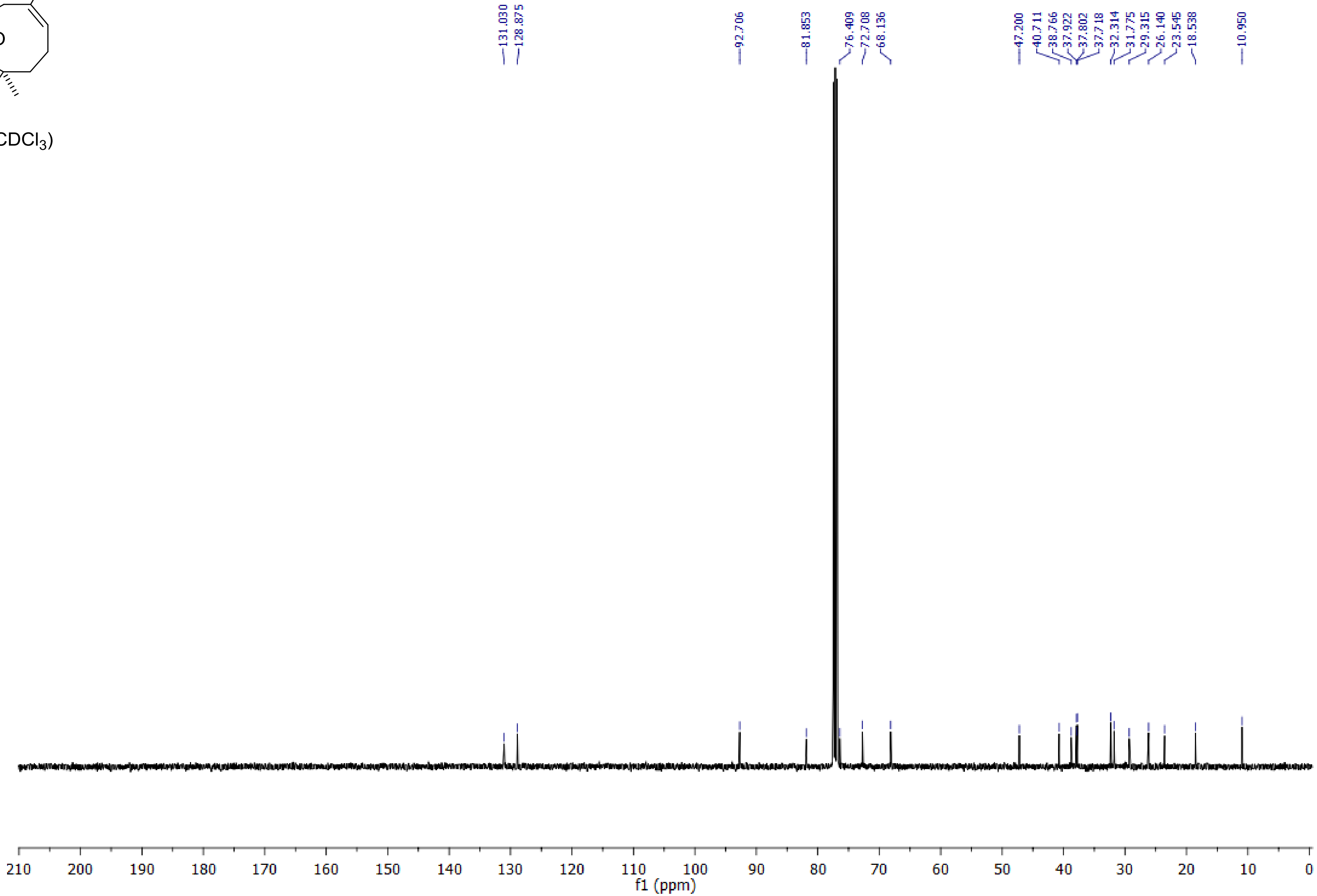

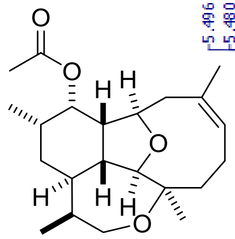

1
(400 MHz, $CDCl_3$)

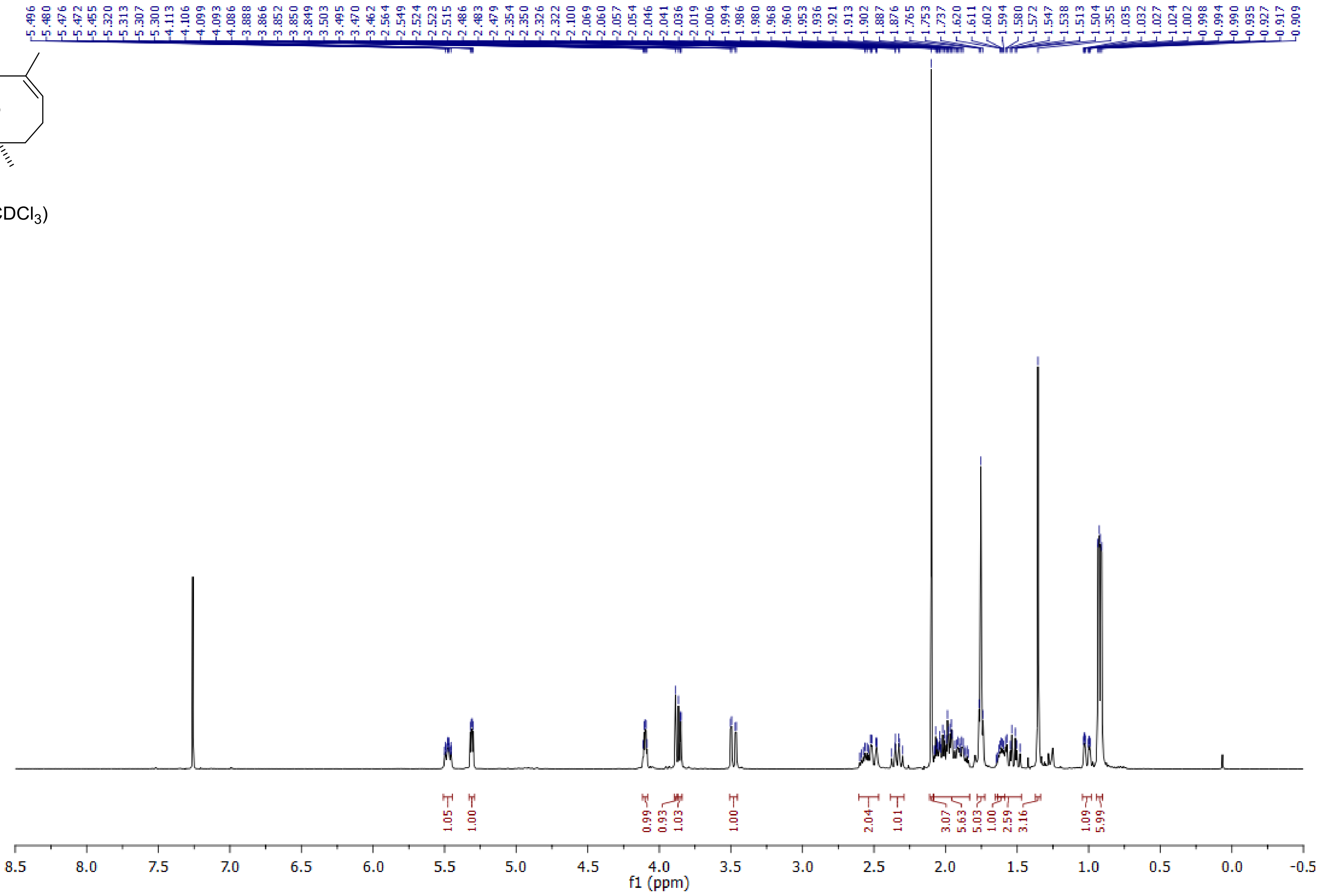

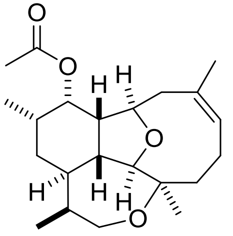

1
(101 MHz, $CDCl_3$)

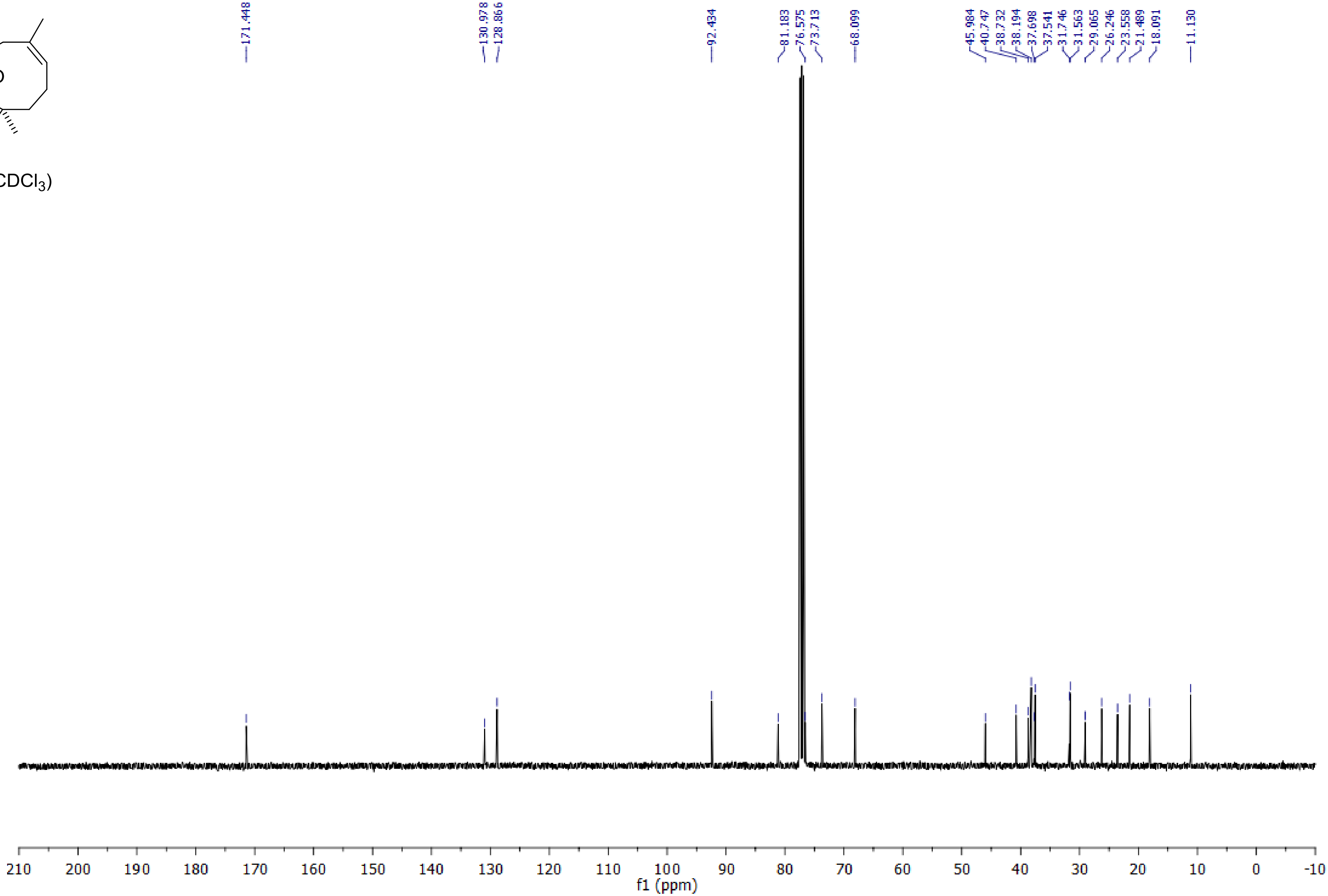

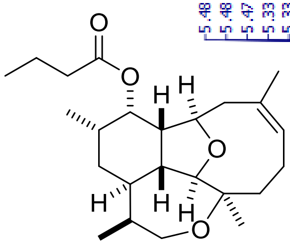

2
(400 MHz, $CDCl_3$)

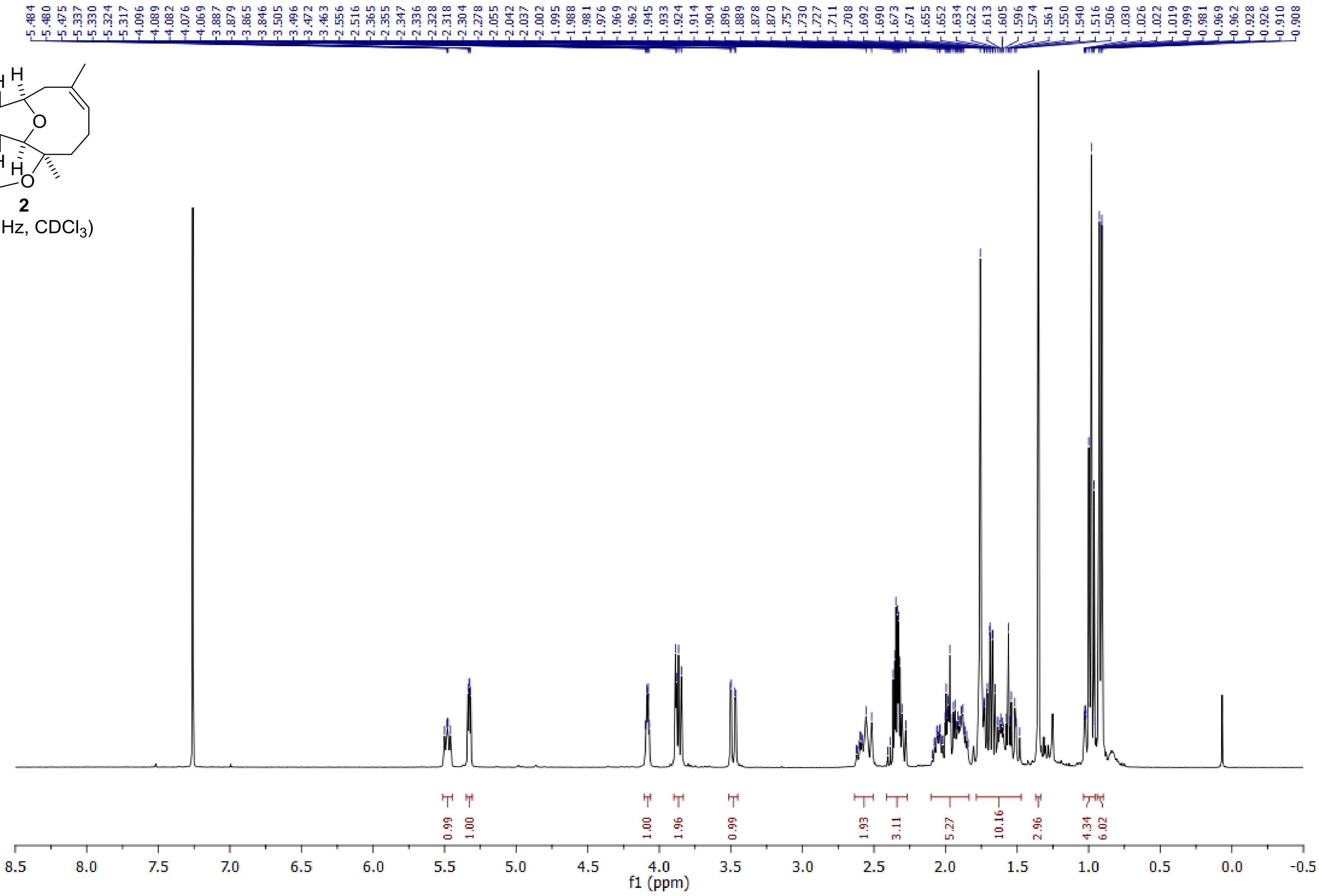

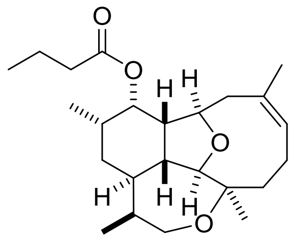

2

(101 MHz, $CDCl_3$)

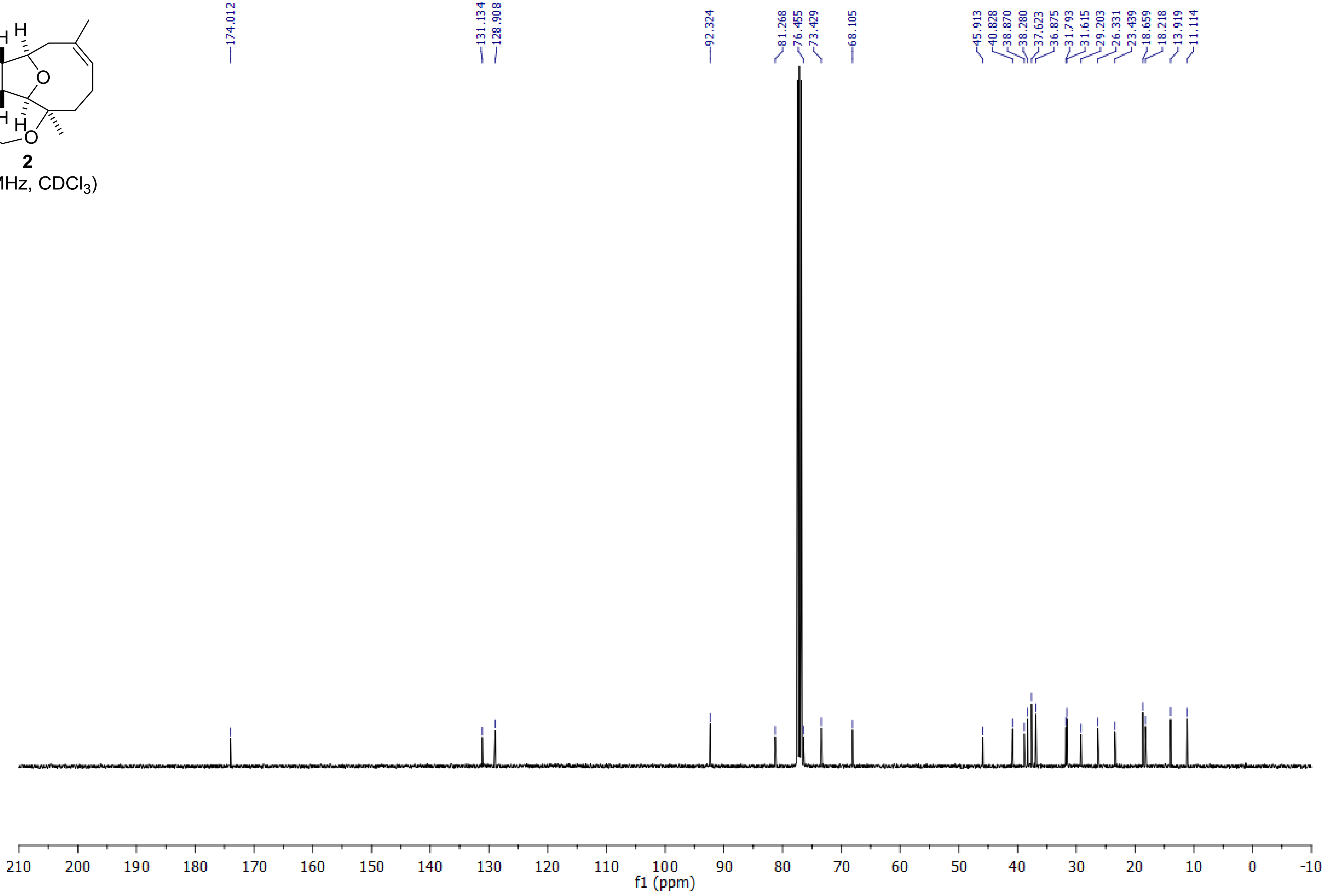

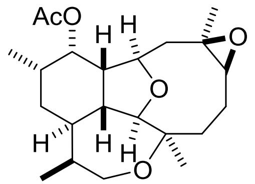

40a

(400 MHz, $CDCl_3$)

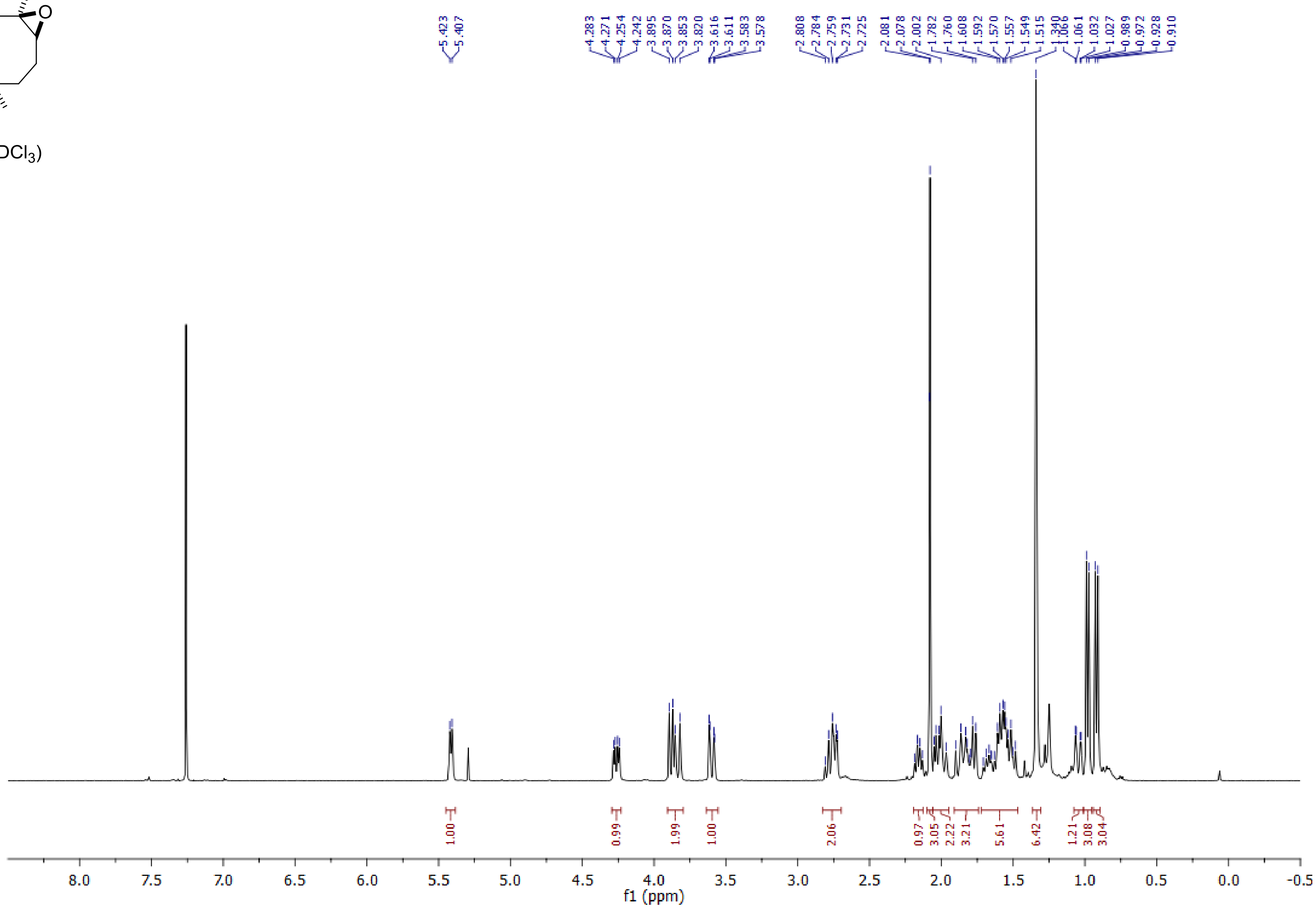

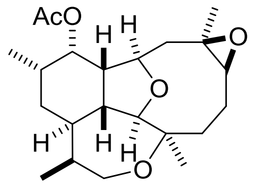

40a

(101 MHz, $CDCl_3$)

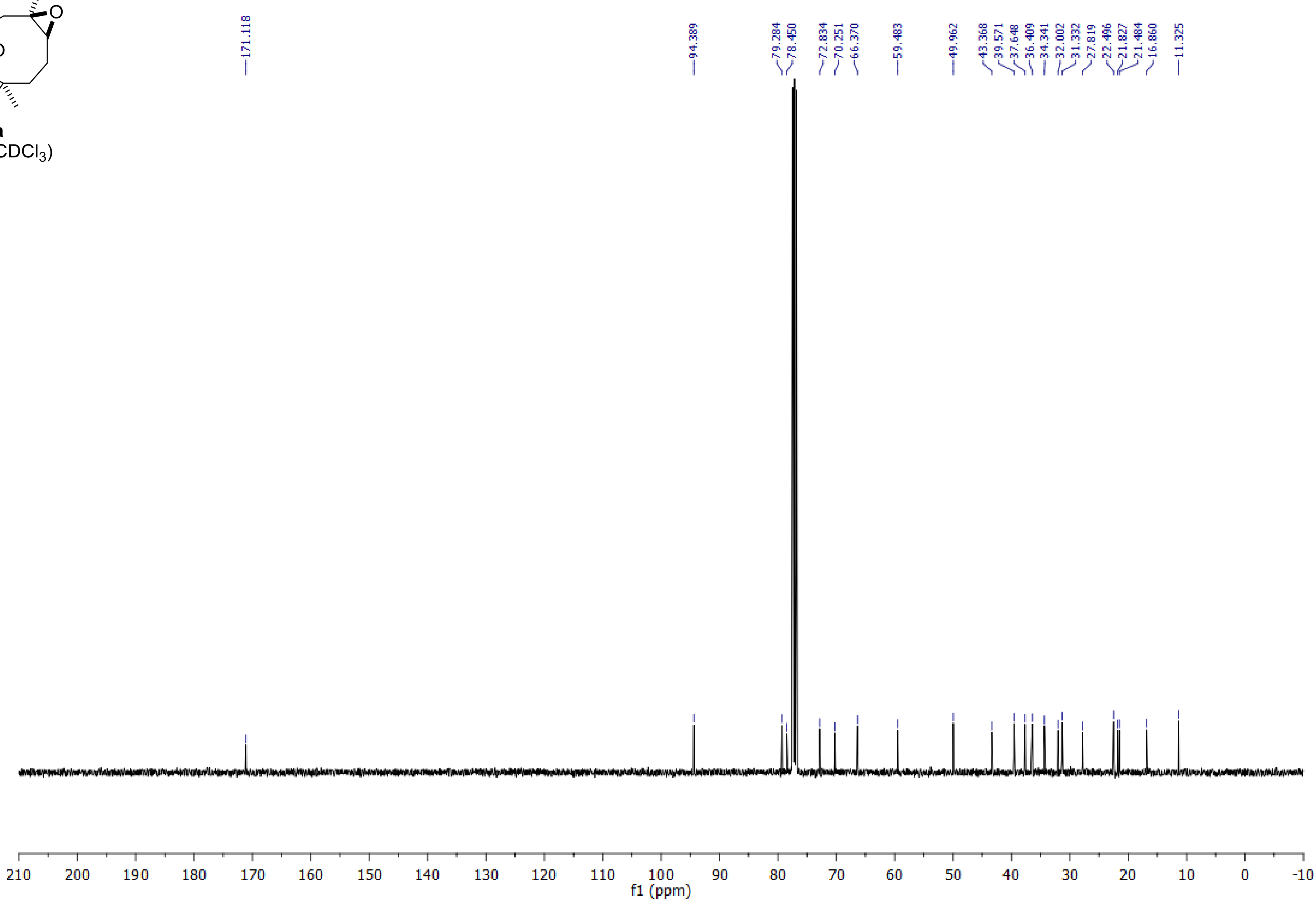

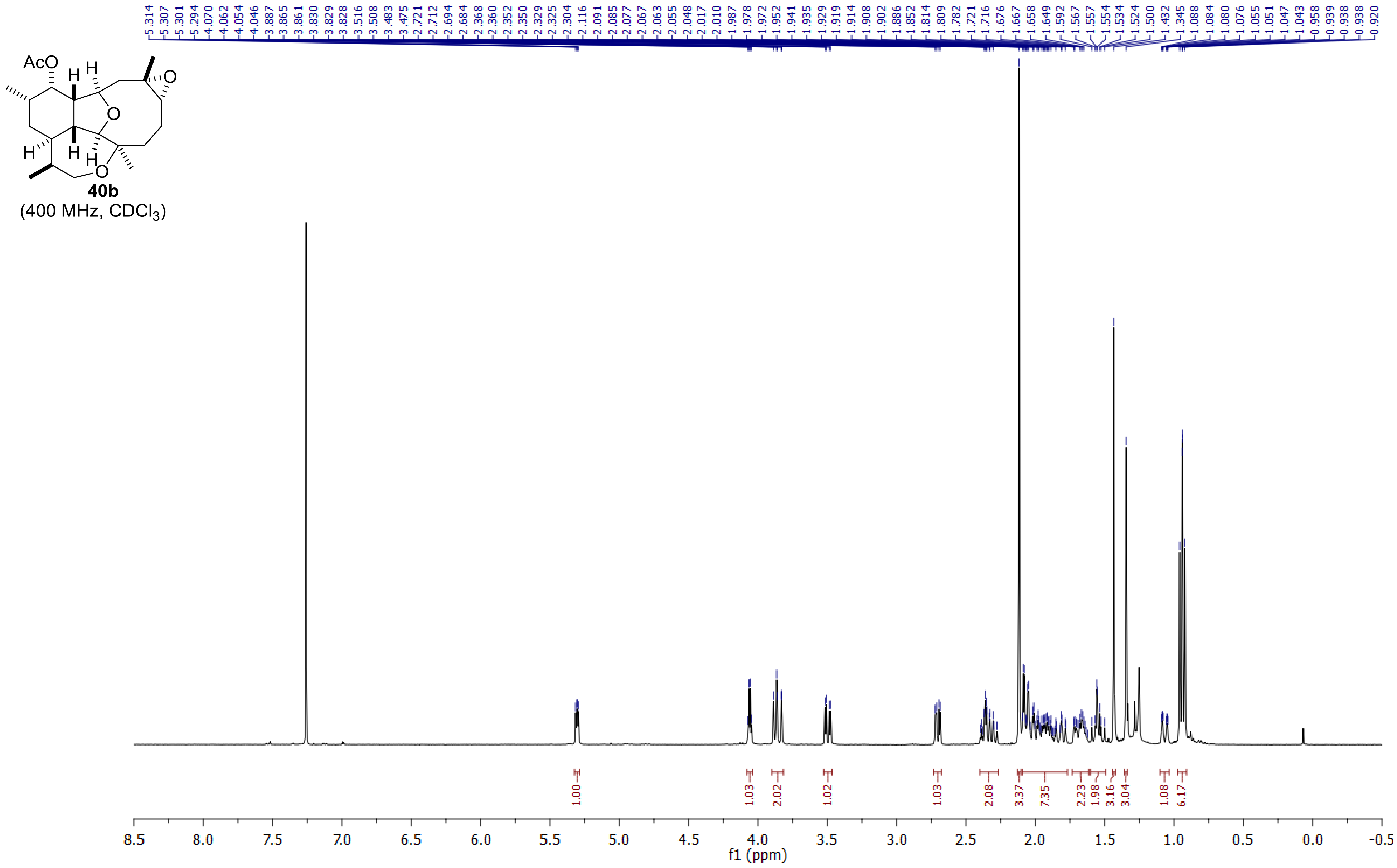
AcO
H
H
O
O
H
H
H
O
40b
(400 MHz, CDCl3)
f1 (ppm)

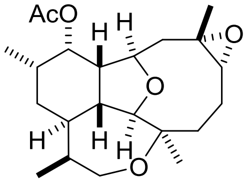

40b

(101 MHz, $CDCl_3$)

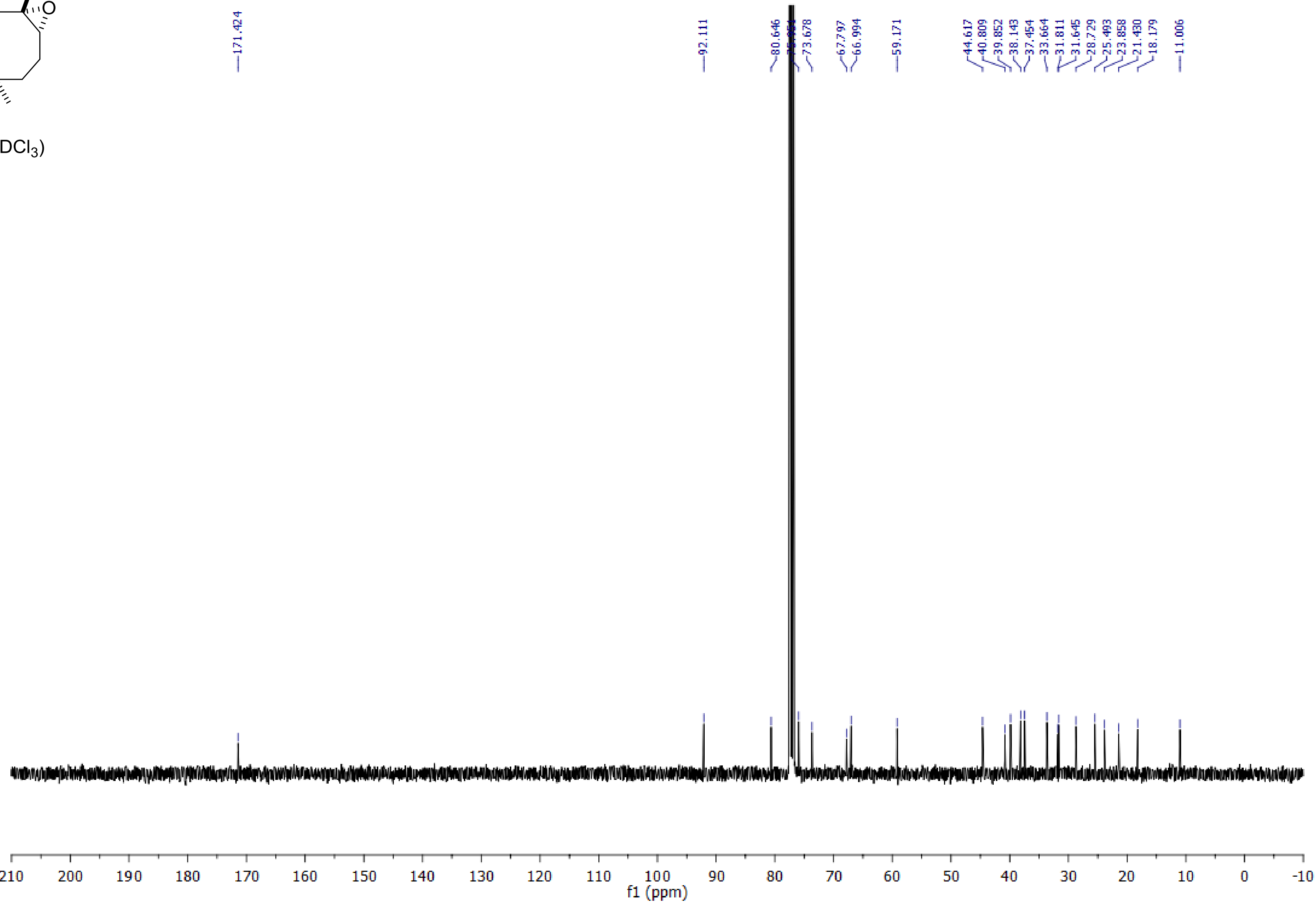

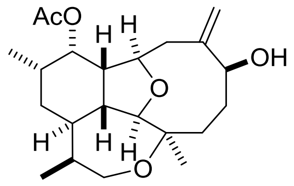

6

(500 MHz, $CDCl_3$)

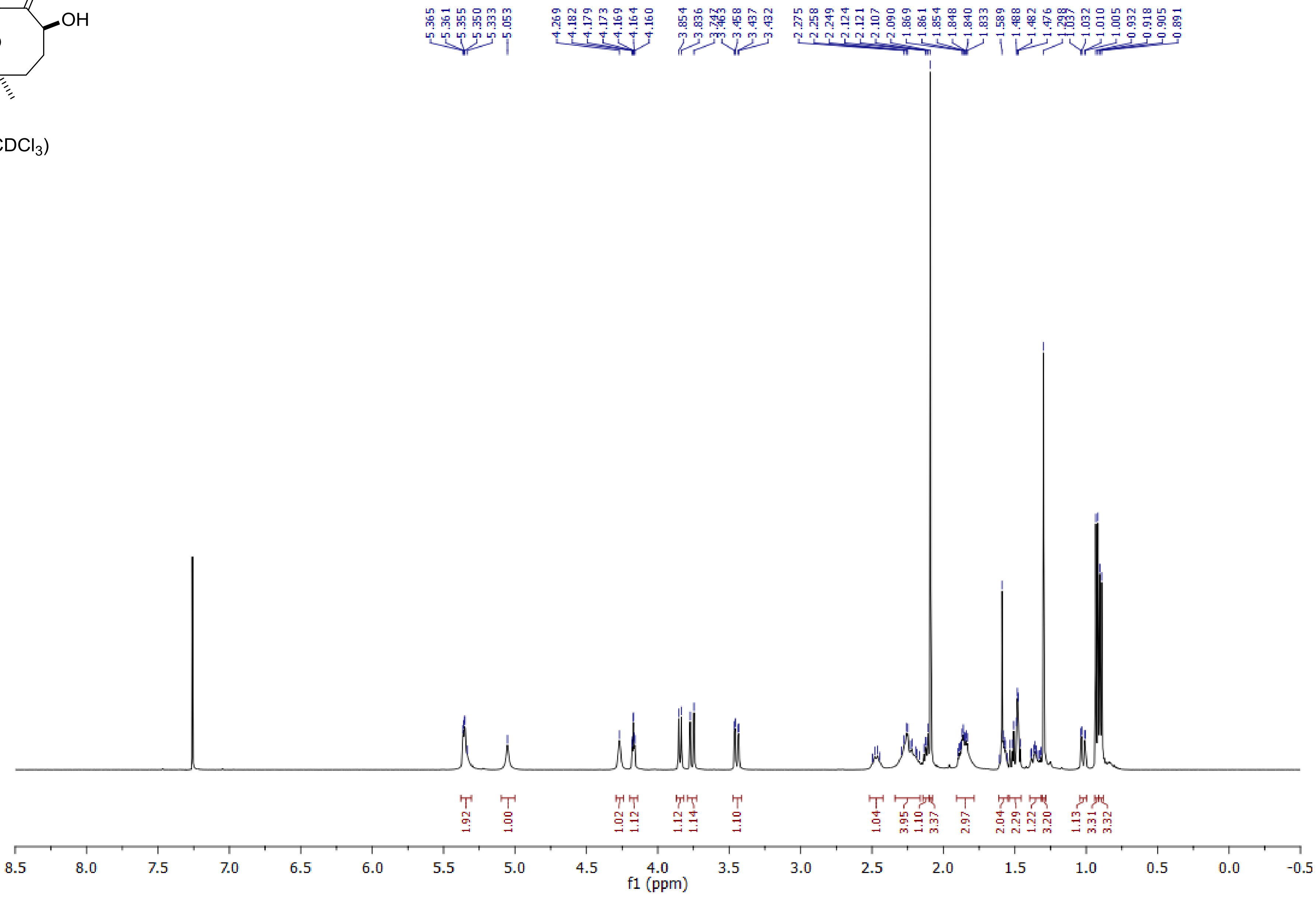

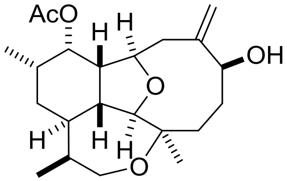

6

(126 MHz, $CDCl_3$)

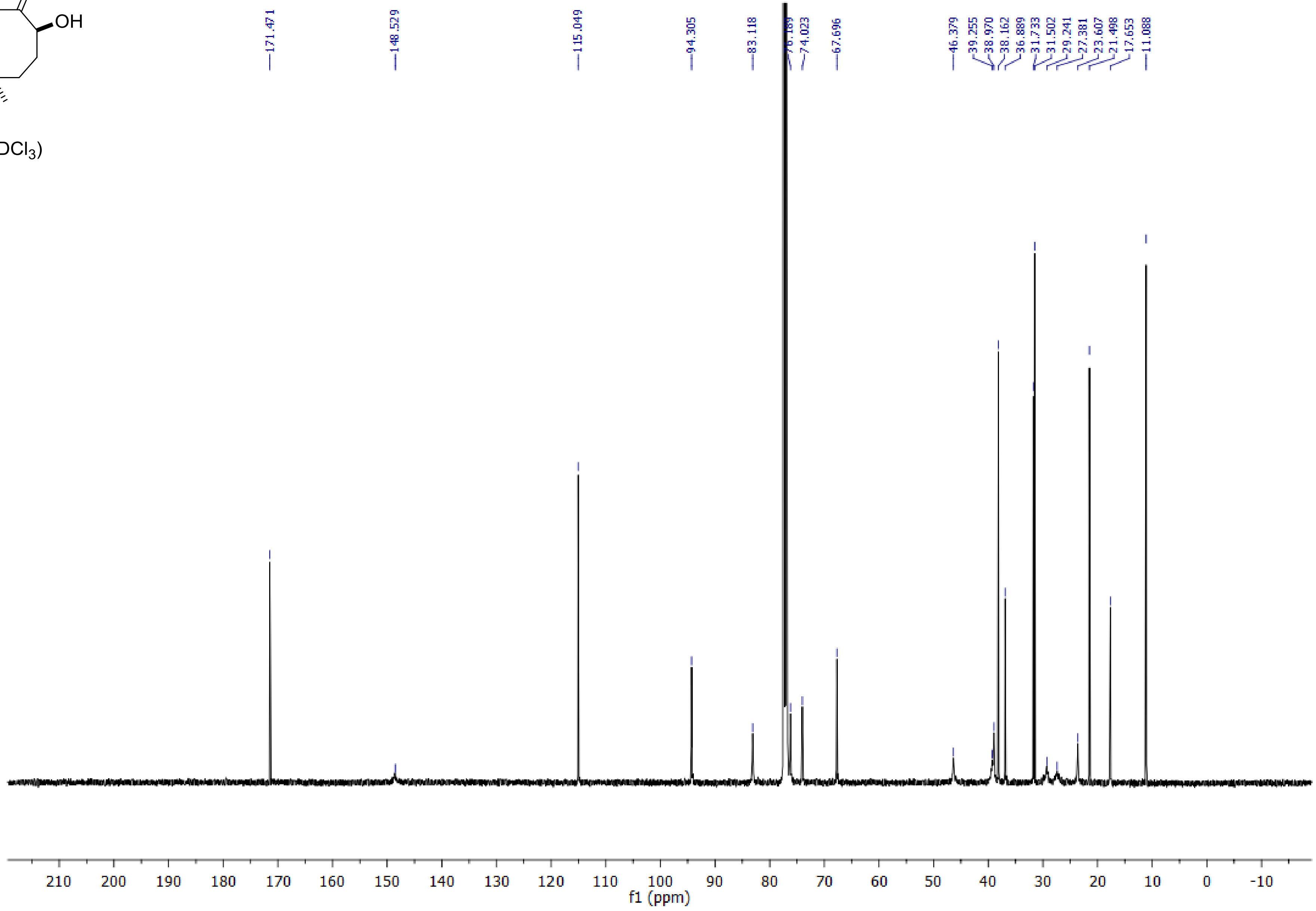

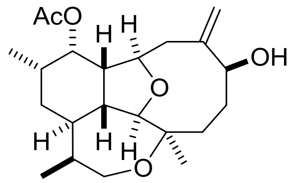
AcO
H
H
OH
O
H
H
H
O
6

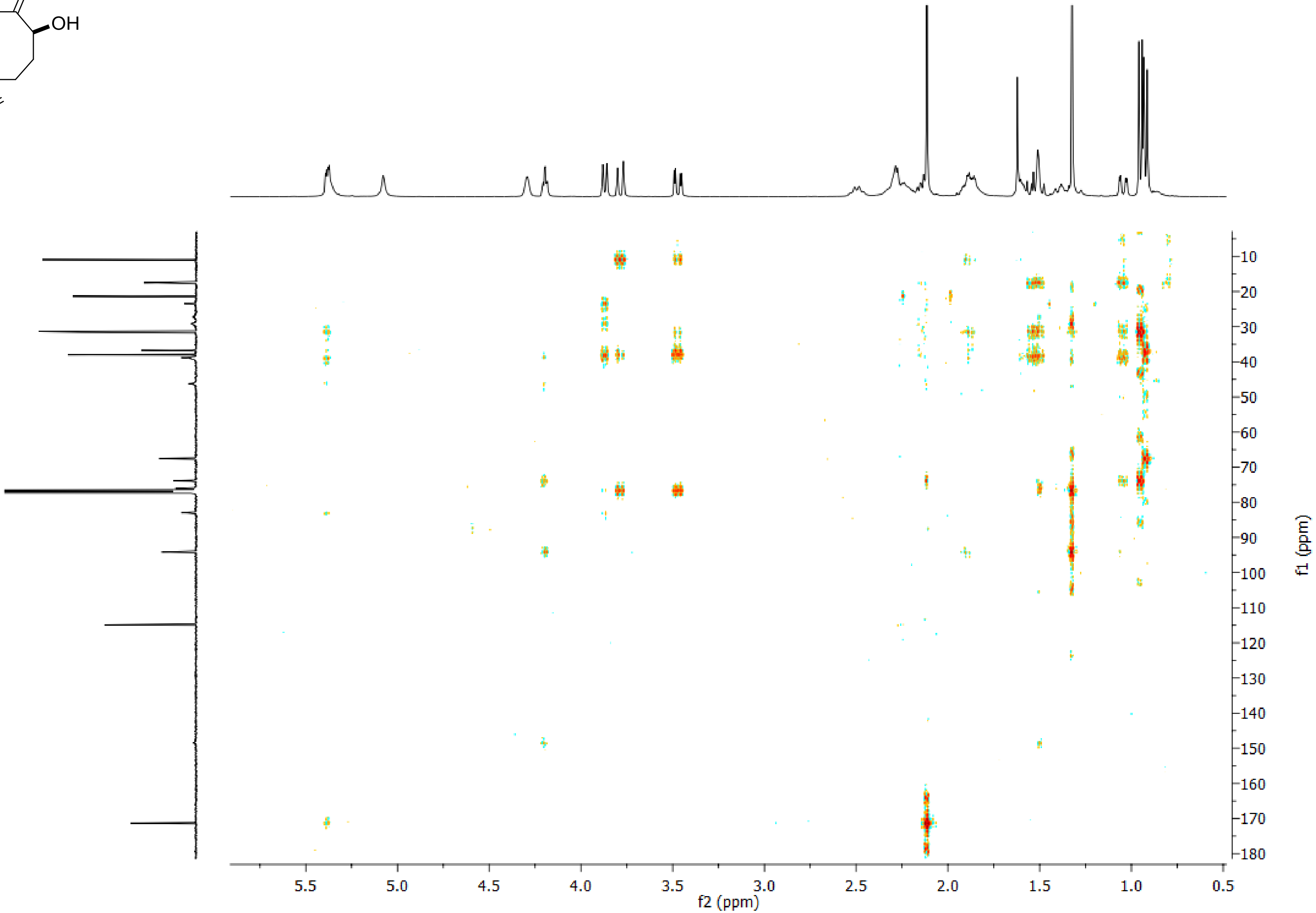
5.5
5.0
4.5
4.0
3.5
3.0
2.5
2.0
1.5
1.0
0.5
f2 (ppm)
10
20
30
40
50
60
70
80
90
100
110
120
130
140
150
160
170
180
f1 (ppm)

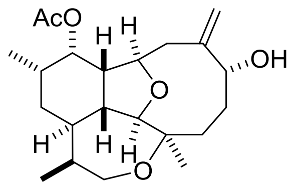

9

(400 MHz, $CDCl_3$)

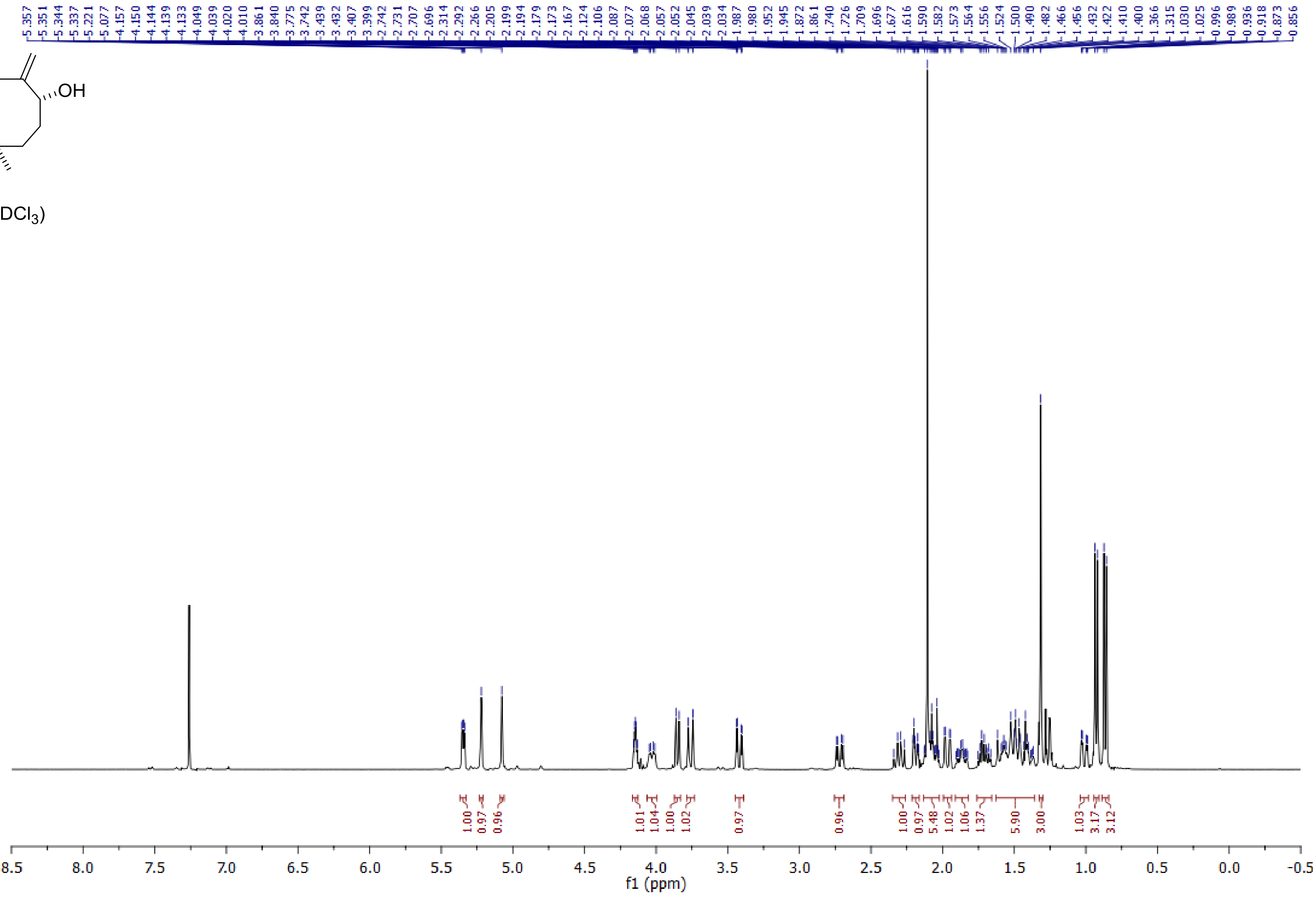

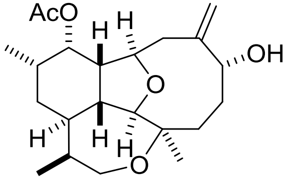

9

(101 MHz, $CDCl_3$)

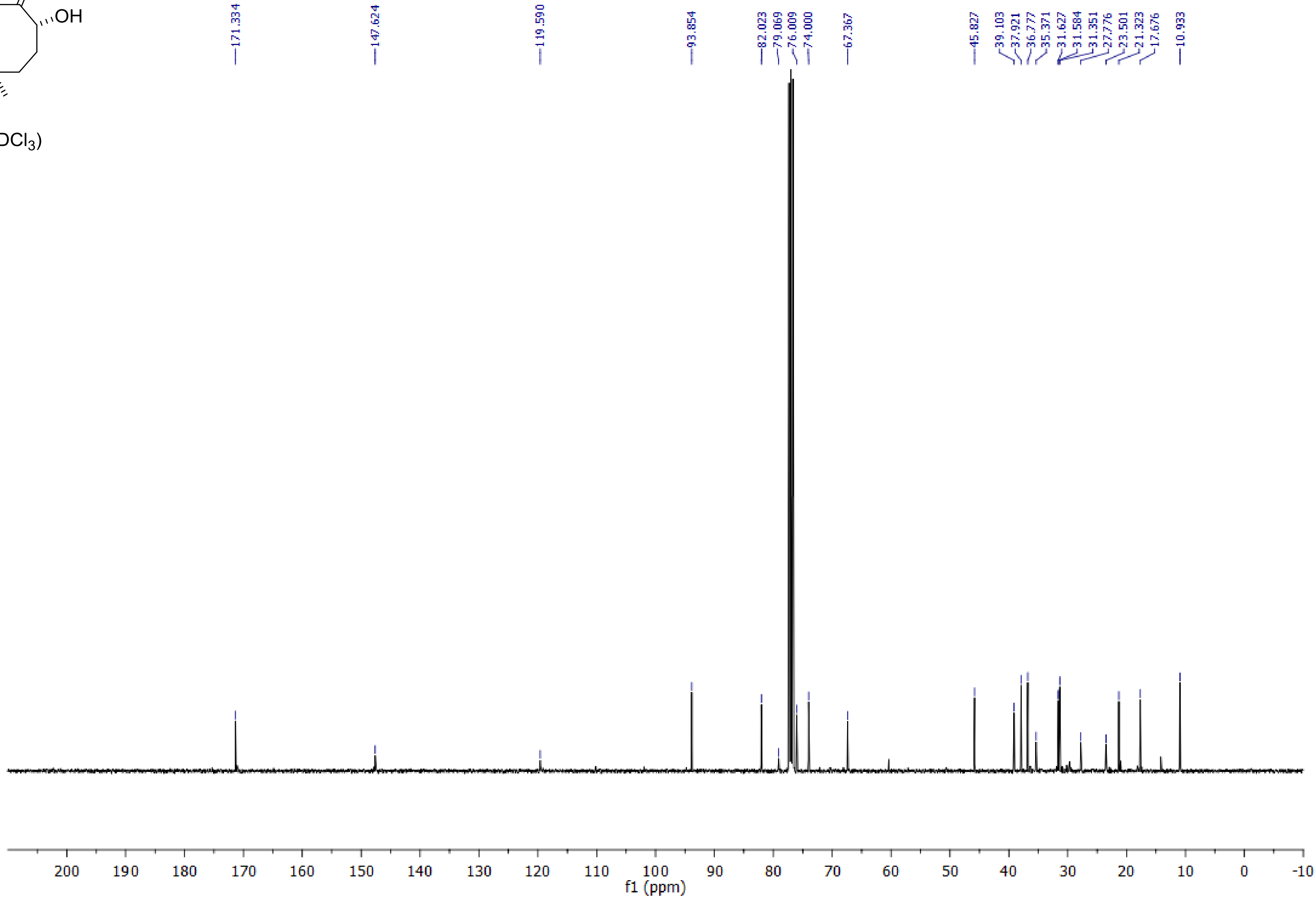

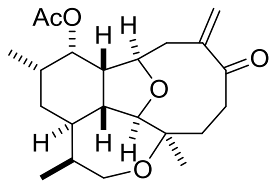

14
(400 MHz, $CDCl_3$)

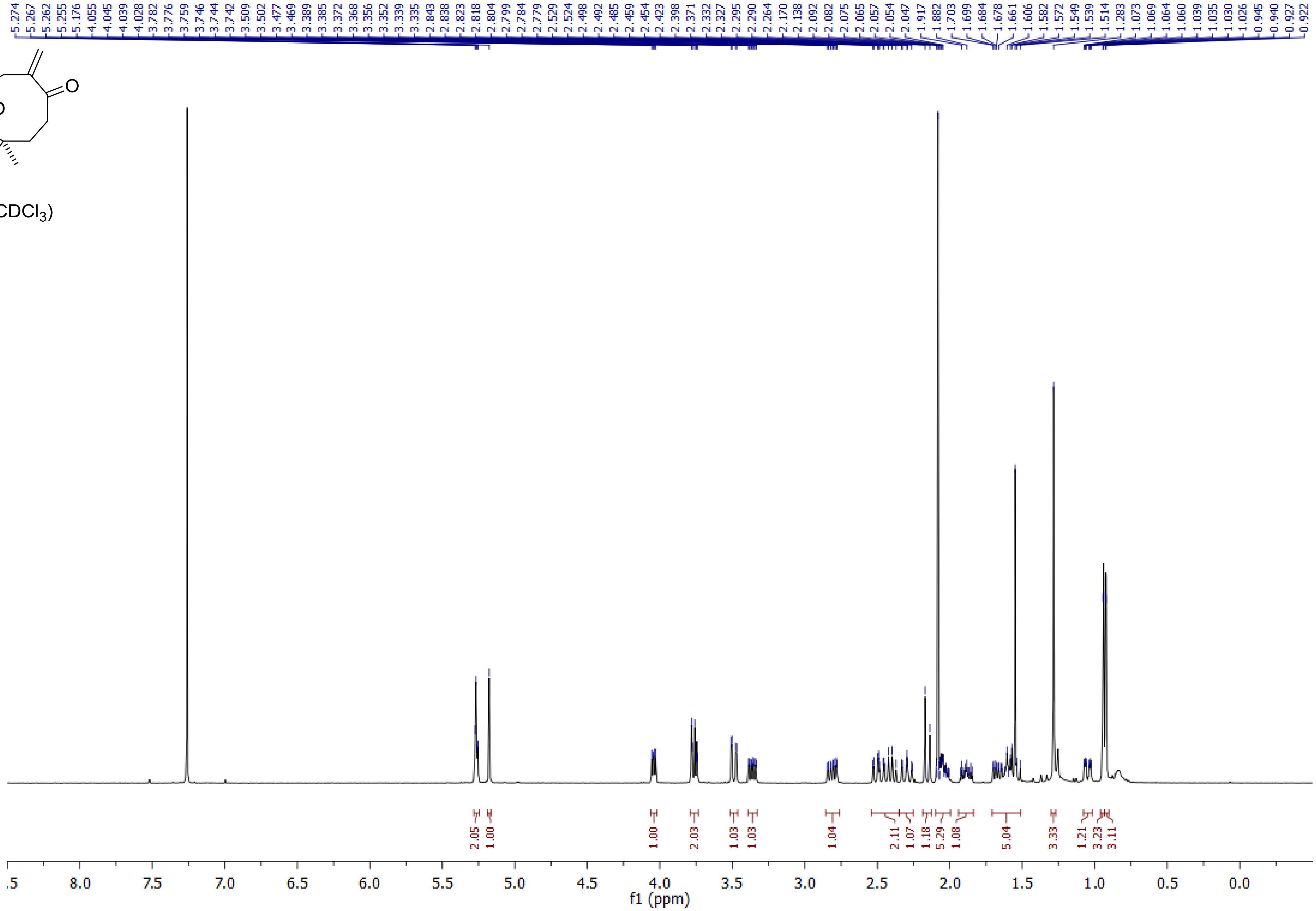

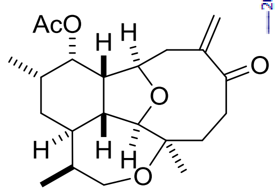

14
(101 MHz, $CDCl_3$)

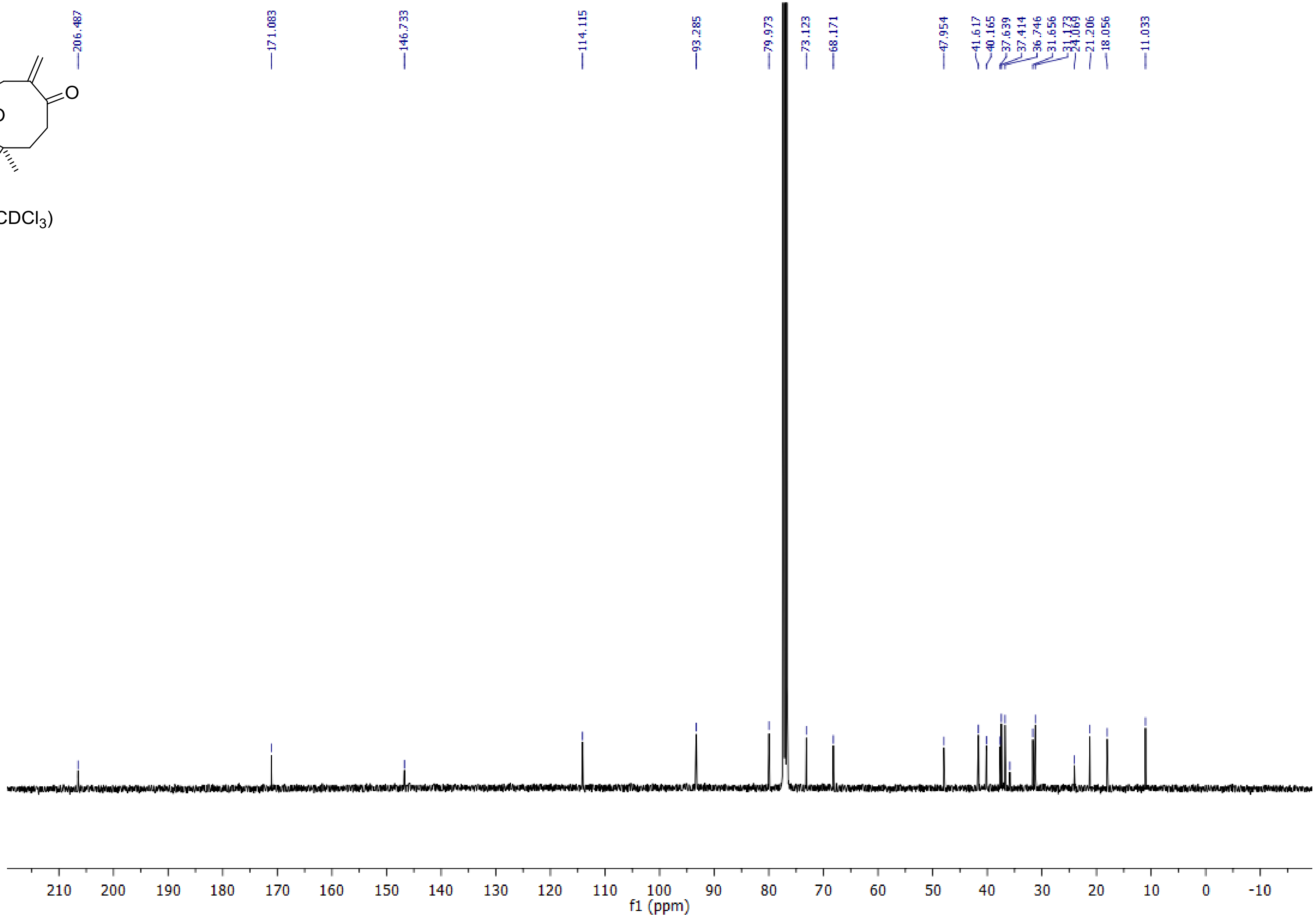

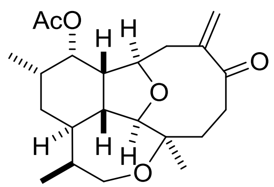

14

(400 MHz, C_6D_6)

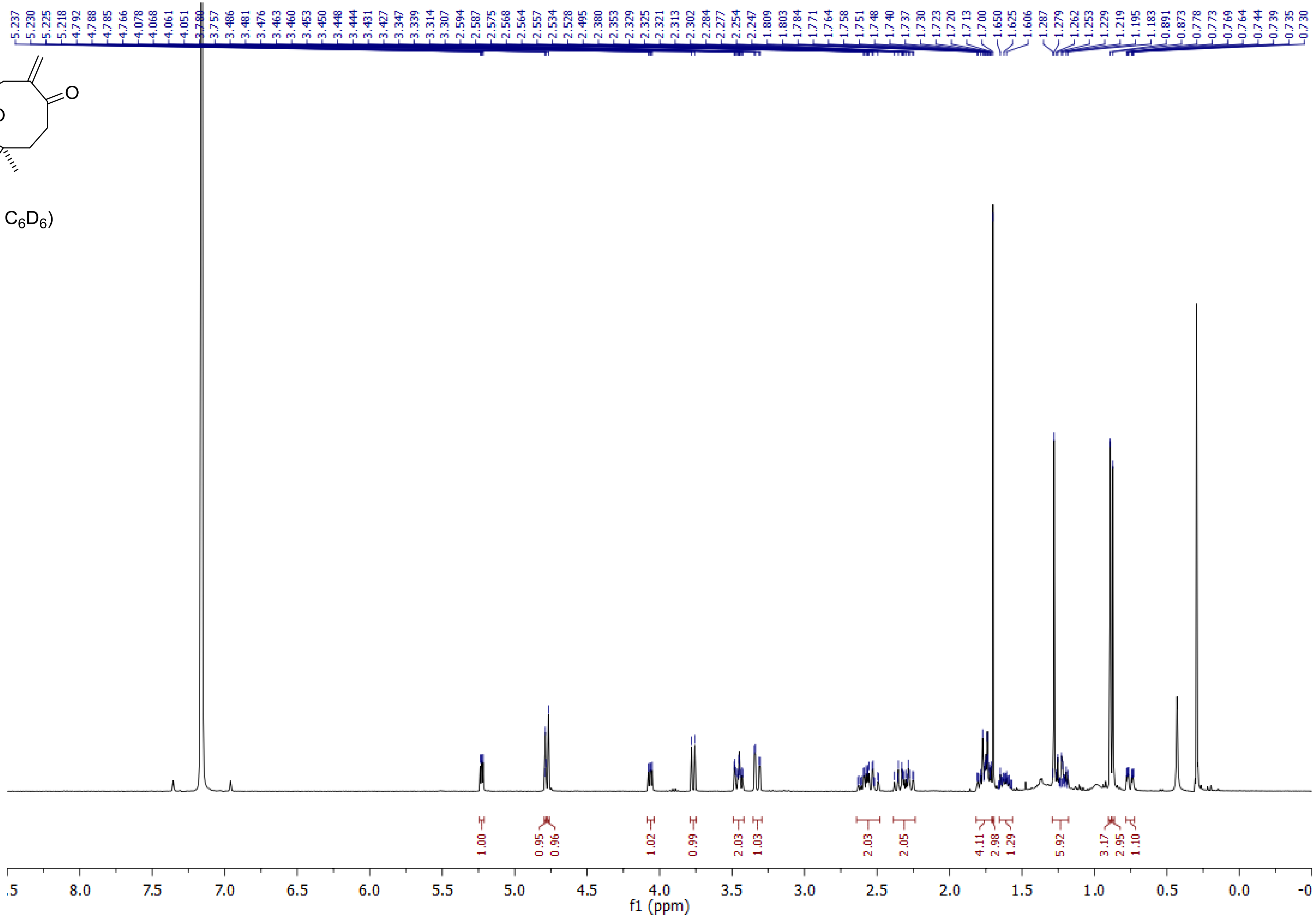

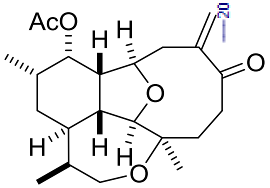

14
(101 MHz, C_6D_6)

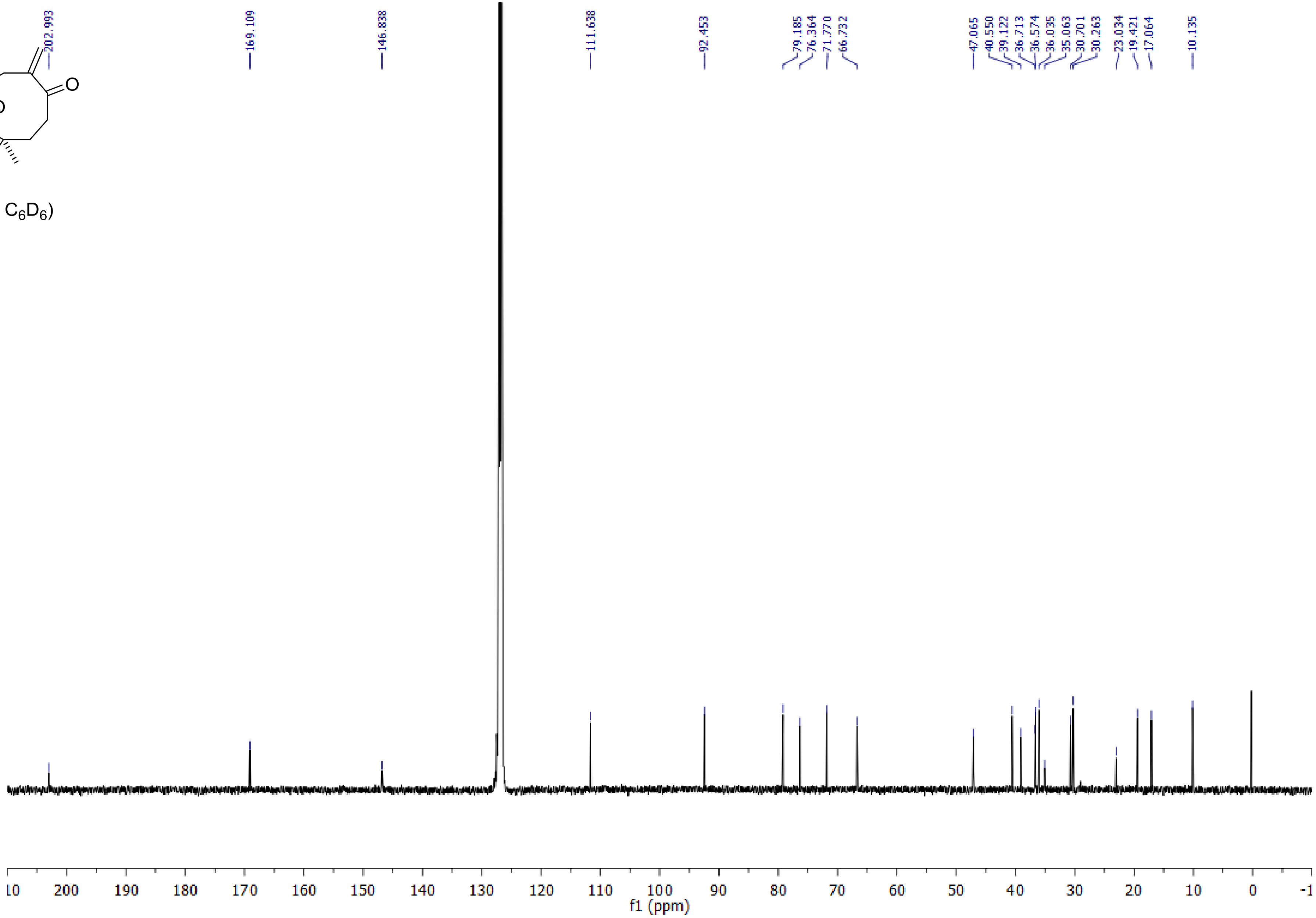

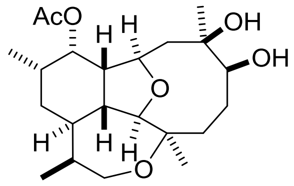

41a
(500 MHz, $CDCl_3$)

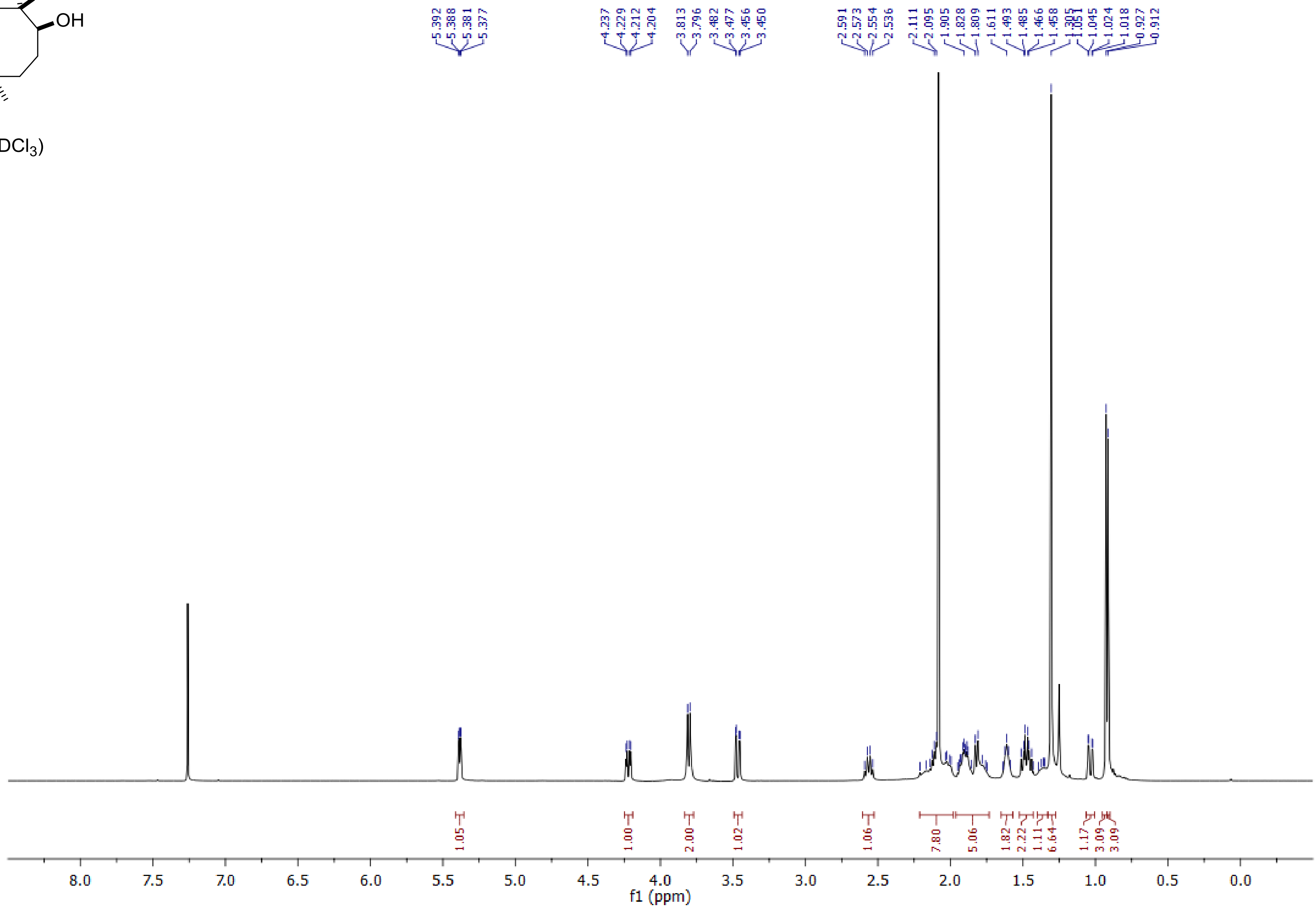

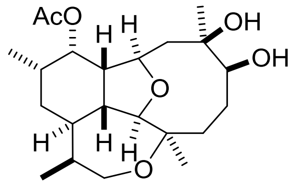

41a
(126 MHz, $CDCl_3$)

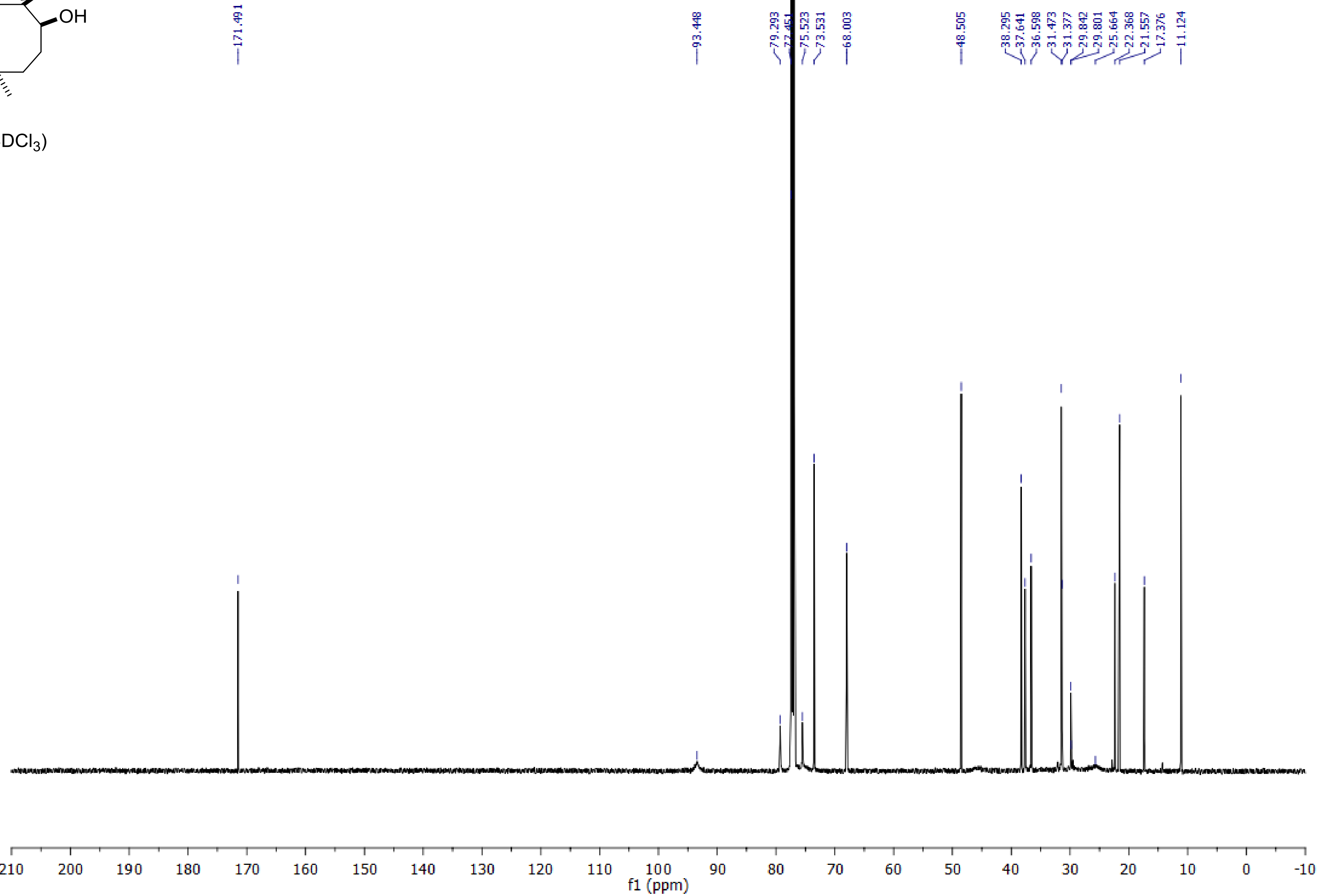

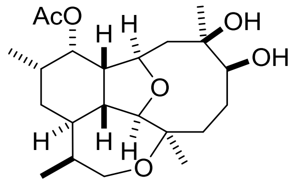
AcO
H
H
OH
OH
O
H
H
H
O

41a

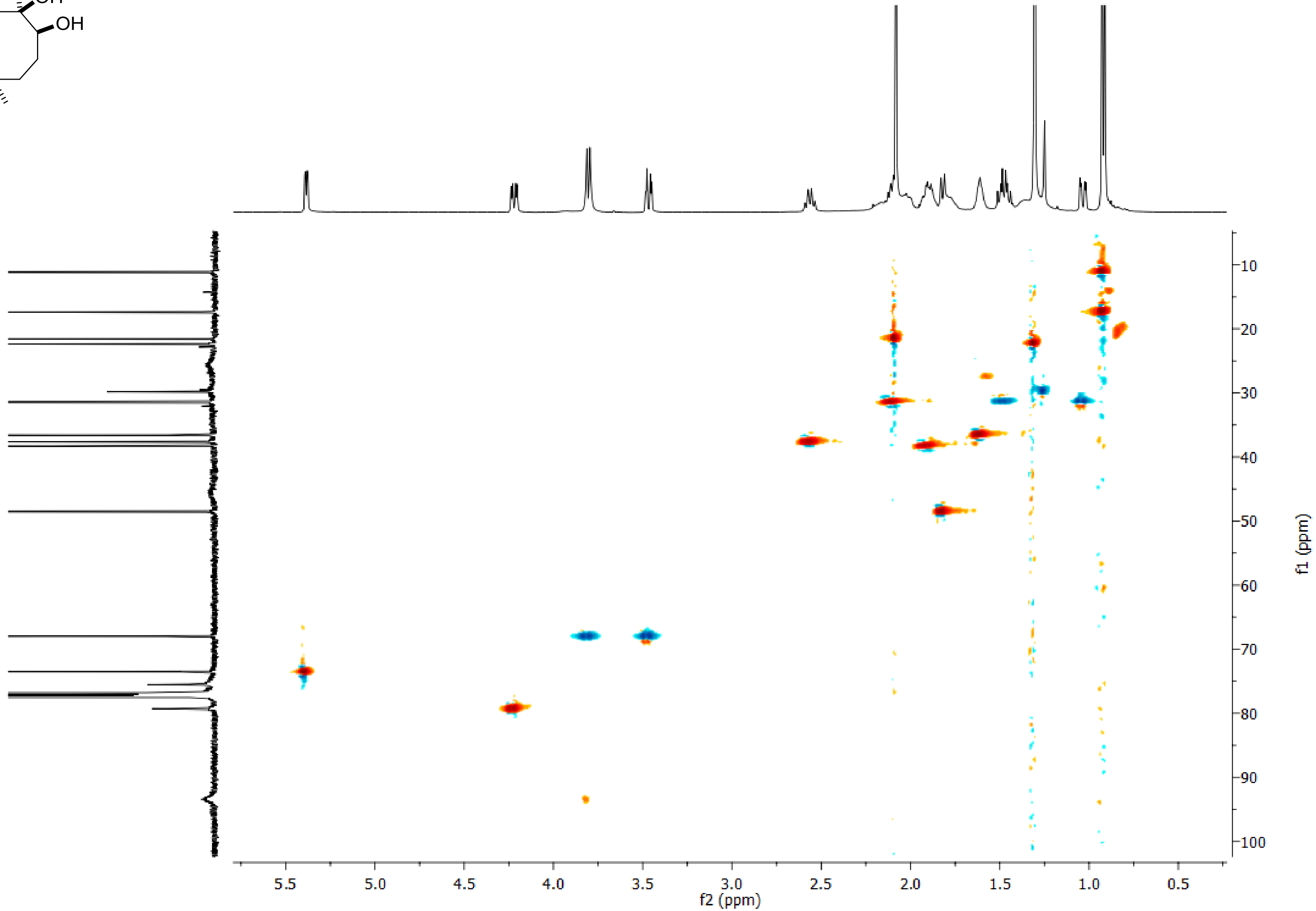
f2 (ppm)
f1 (ppm)
5.5
5.0
4.5
4.0
3.5
3.0
2.5
2.0
1.5
1.0
0.5
10
20
30
40
50
60
70
80
90
100

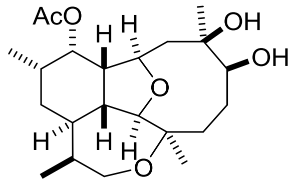
AcO
H
H
OH
OH
O
H
H
H
O

41a

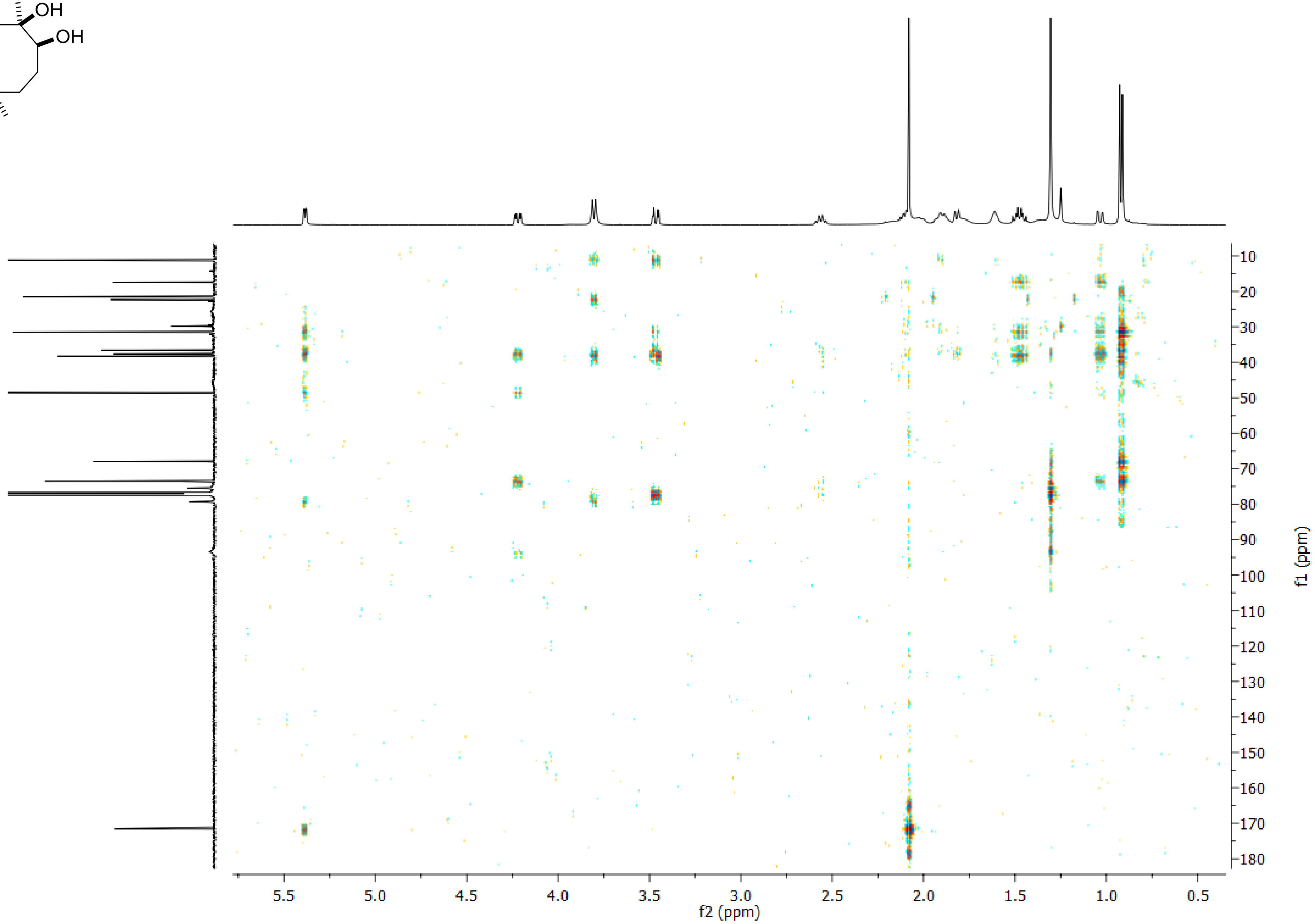
5.5
5.0
4.5
4.0
3.5
3.0
2.5
2.0
1.5
1.0
0.5
f2 (ppm)
10
20
30
40
50
60
70
80
90
100
110
120
130
140
150
160
170
180
f1 (ppm)

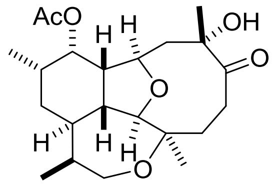

42
(500 MHz, $CDCl_3$)

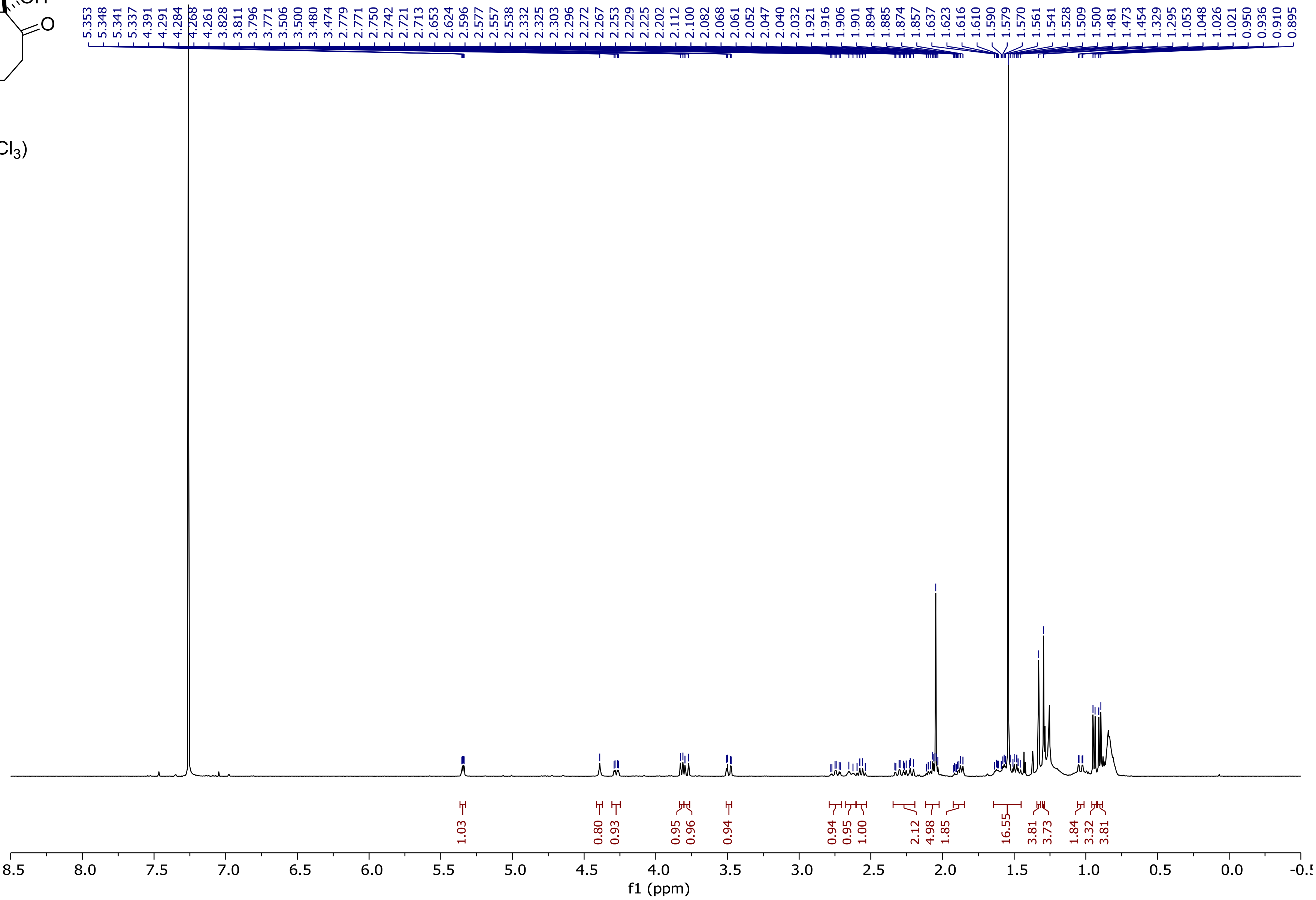

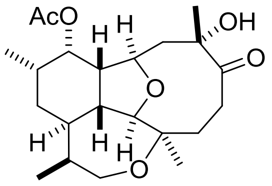

42

(126 MHz, $CDCl_3$)

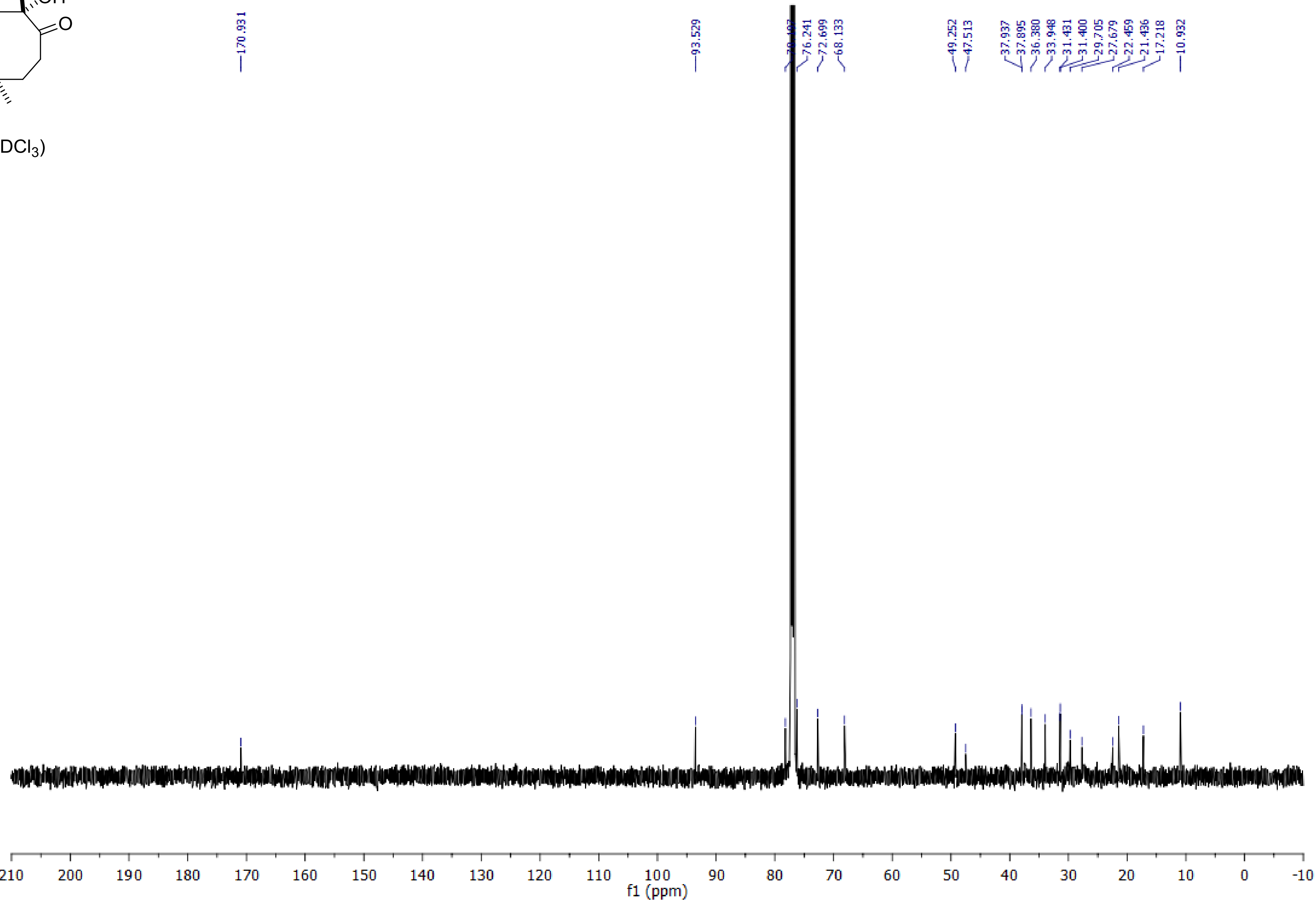

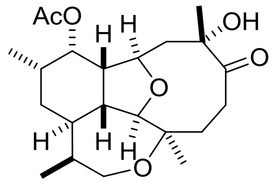

42

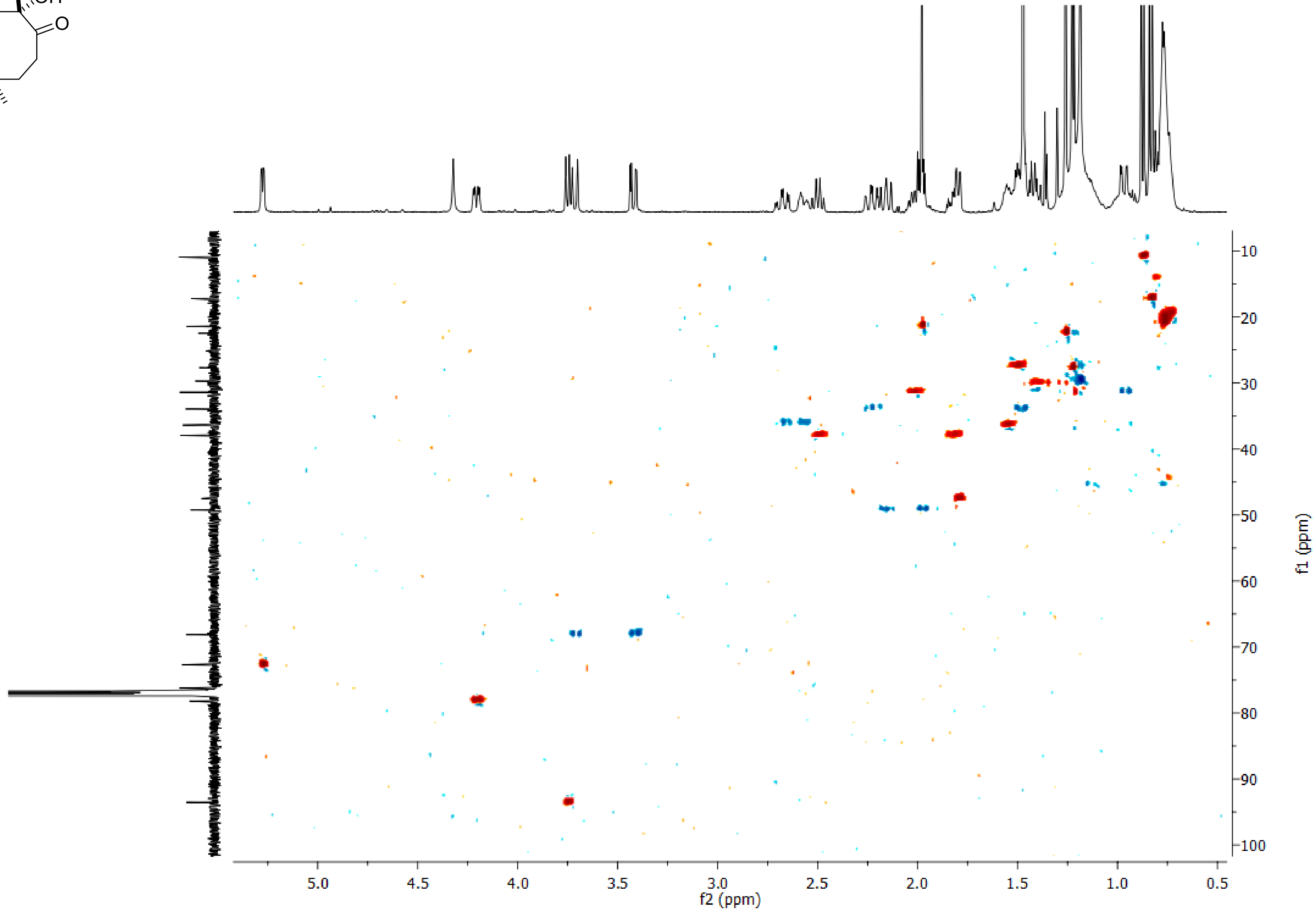

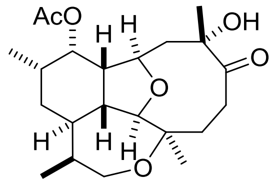

42

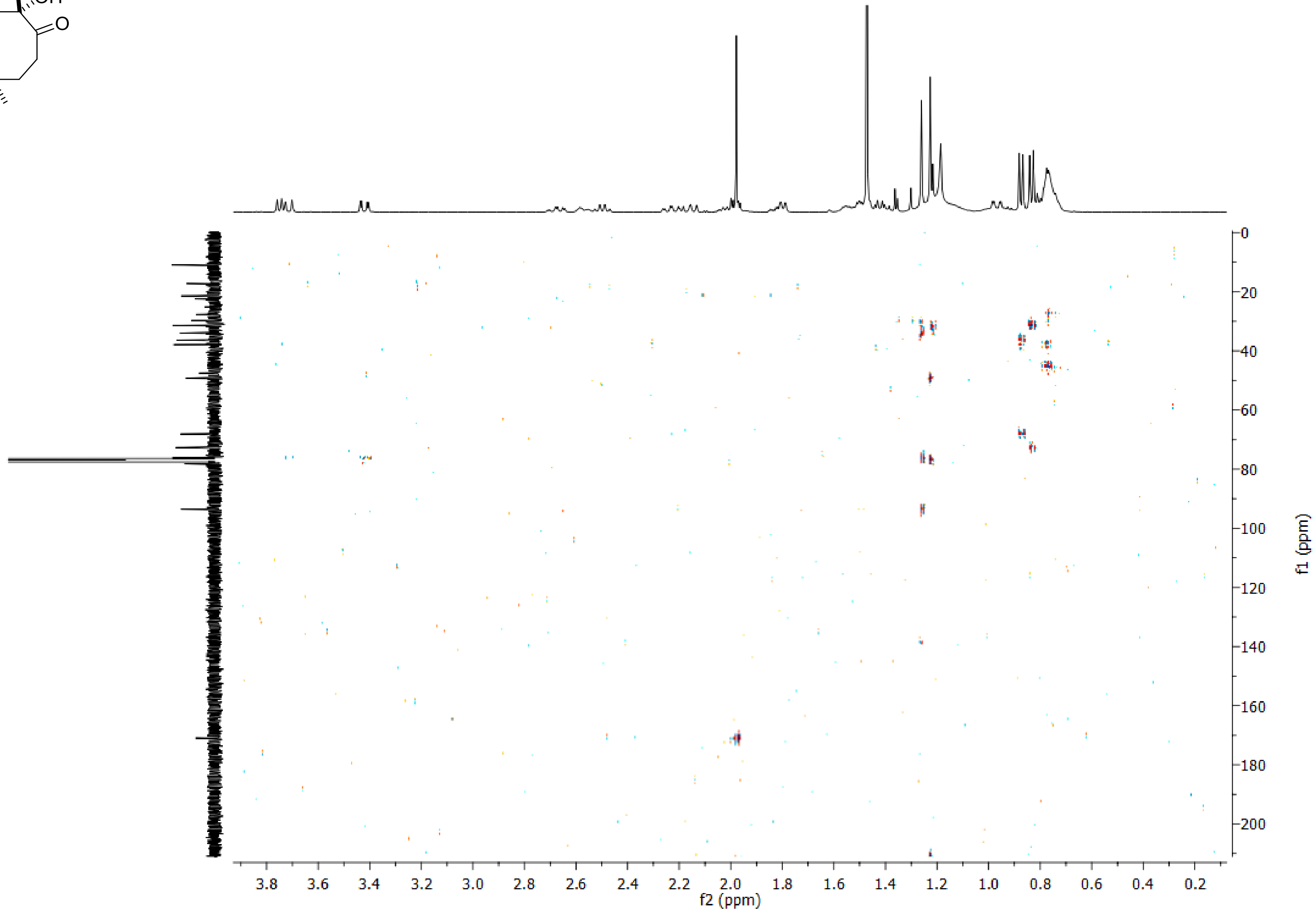

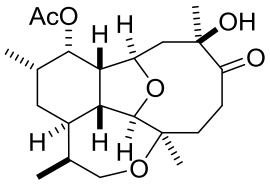

16
(400 MHz, $CDCl_3$)

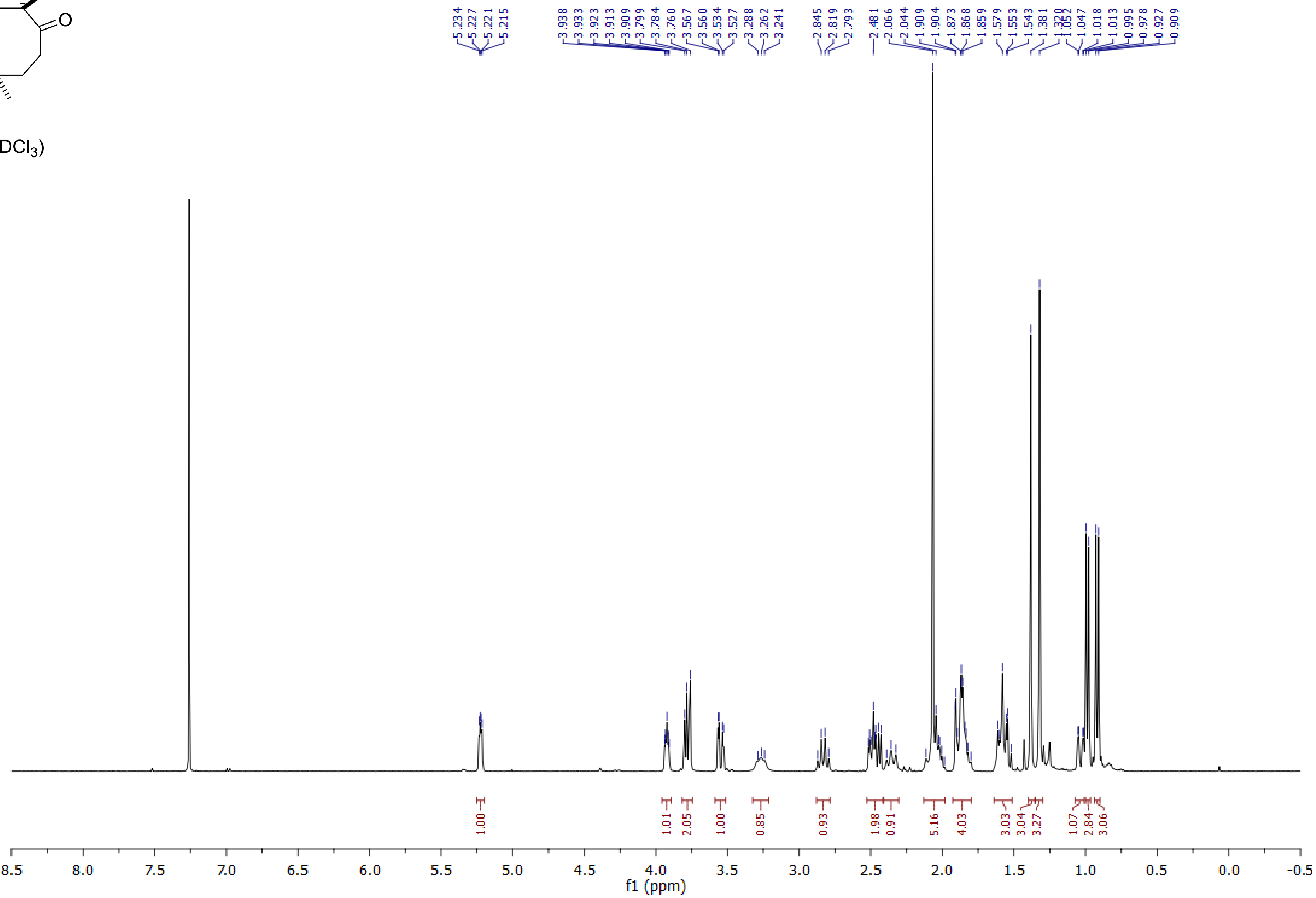

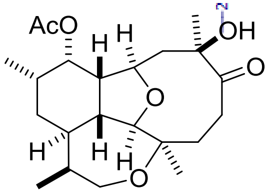

16
(101 MHz, $CDCl_3$)

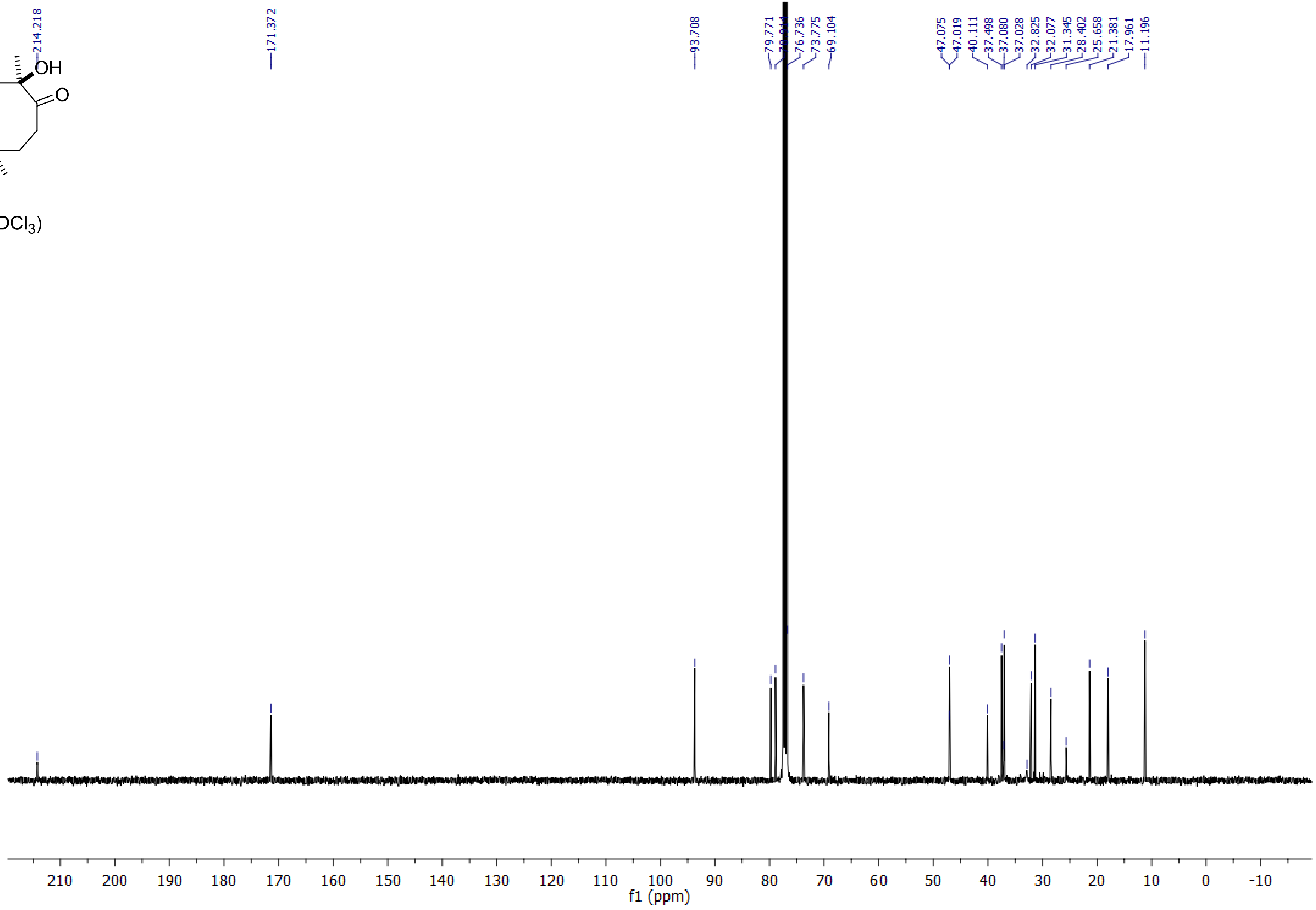

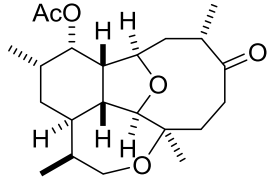

20
(500 MHz, $CDCl_3$)

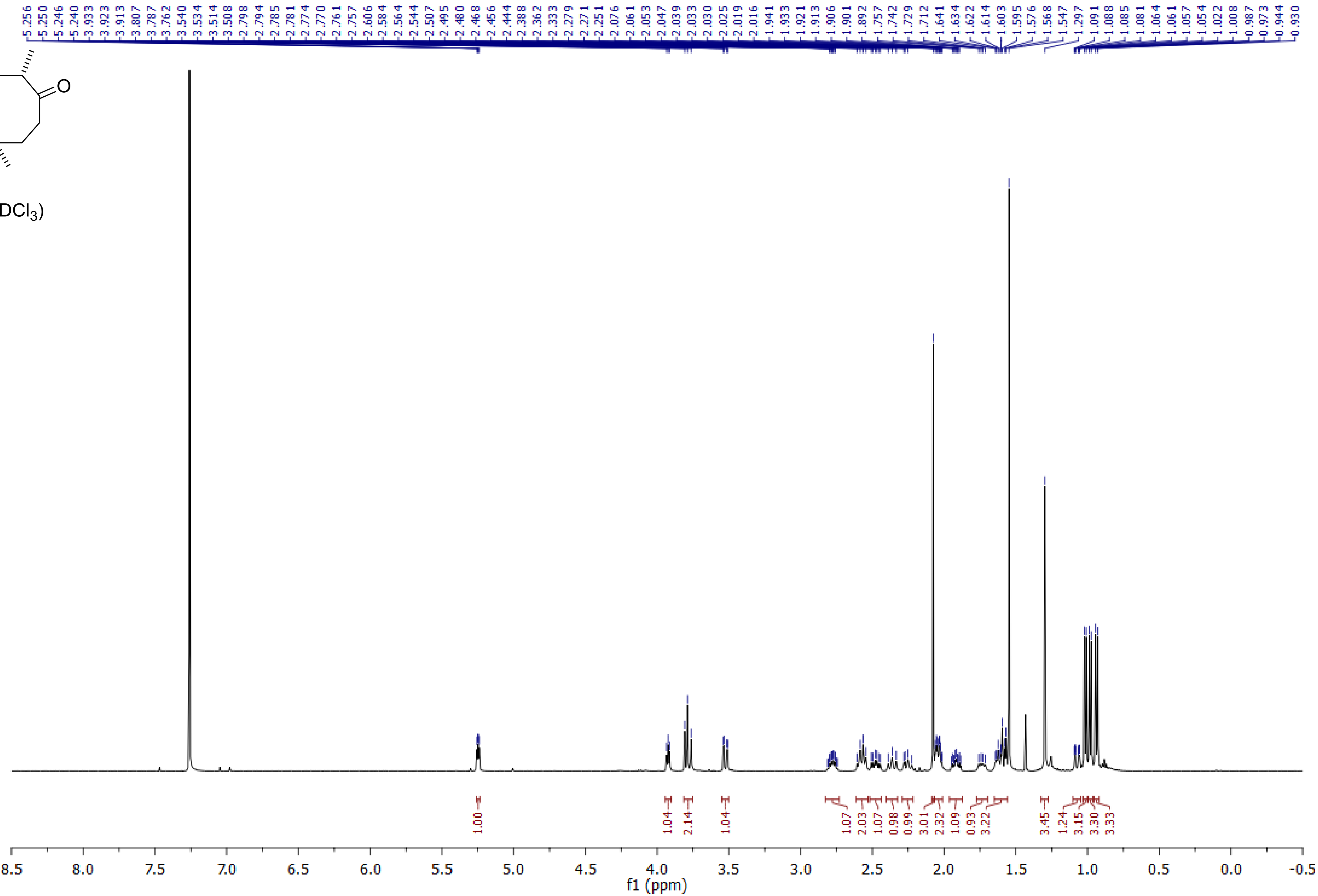

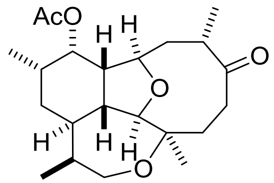

20
(126 MHz, $CDCl_3$)

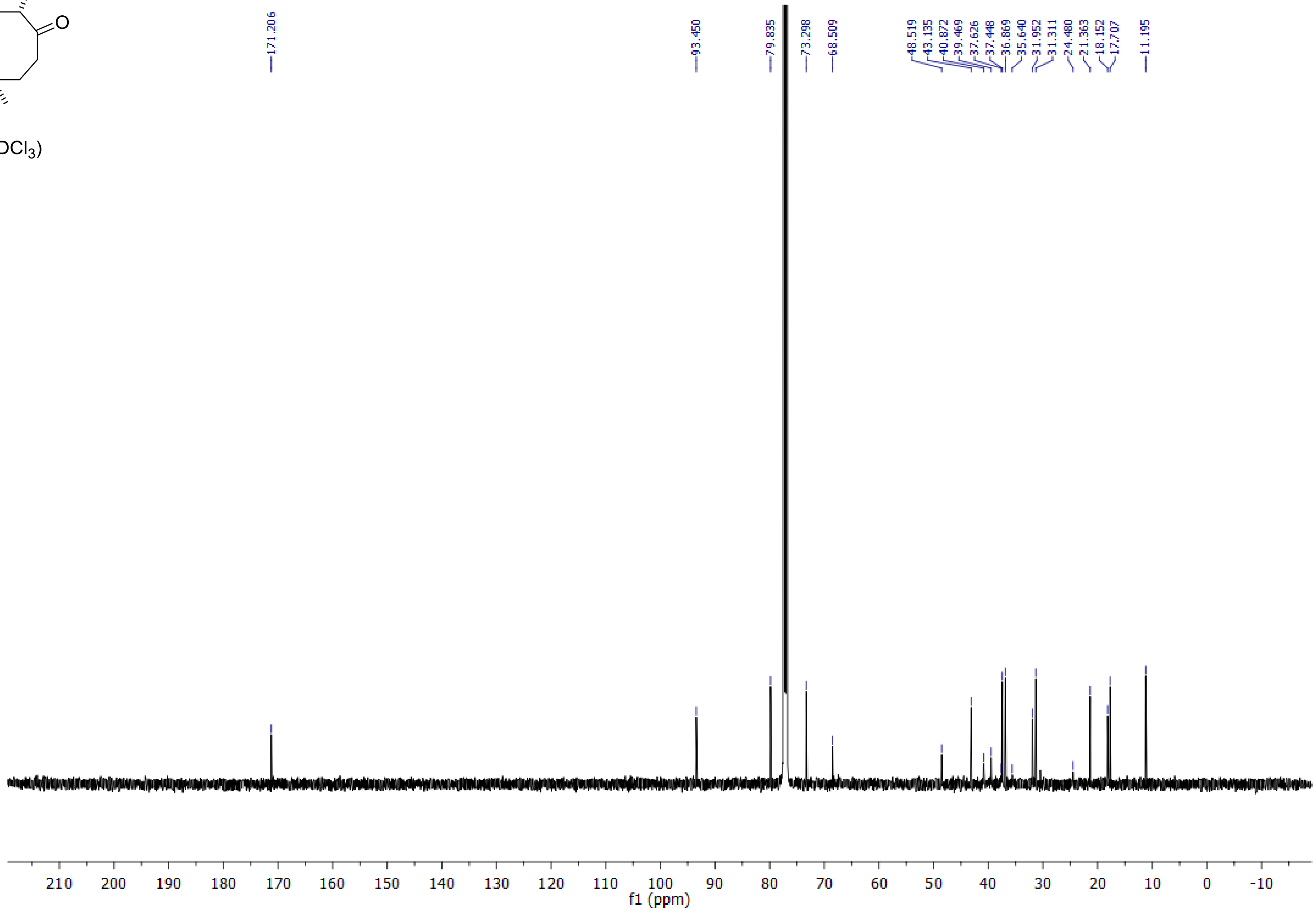

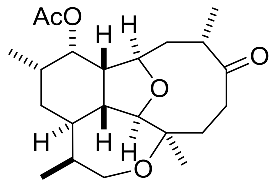

20

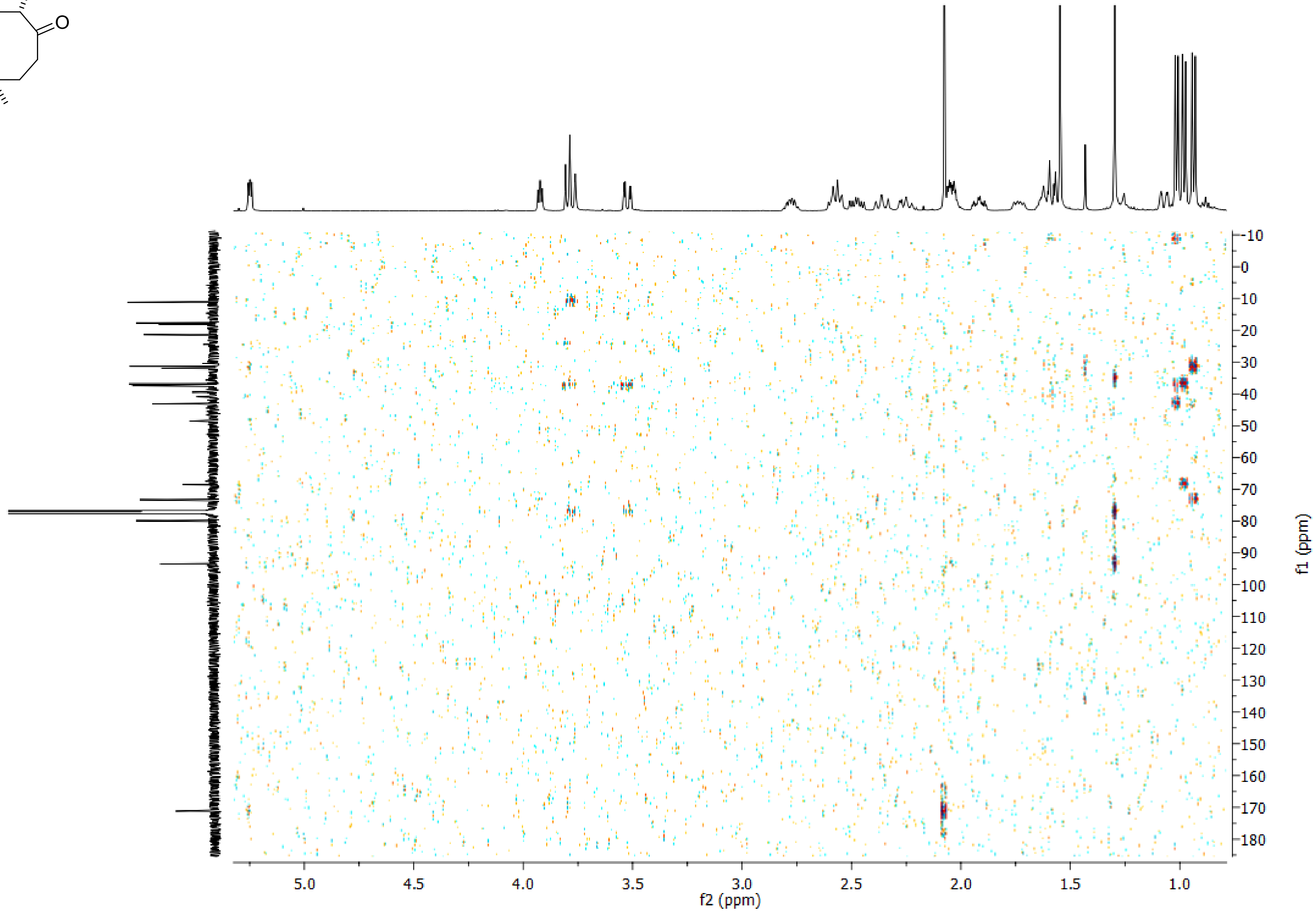

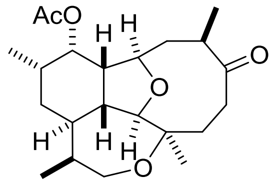

43

(400 MHz, $CDCl_3$)

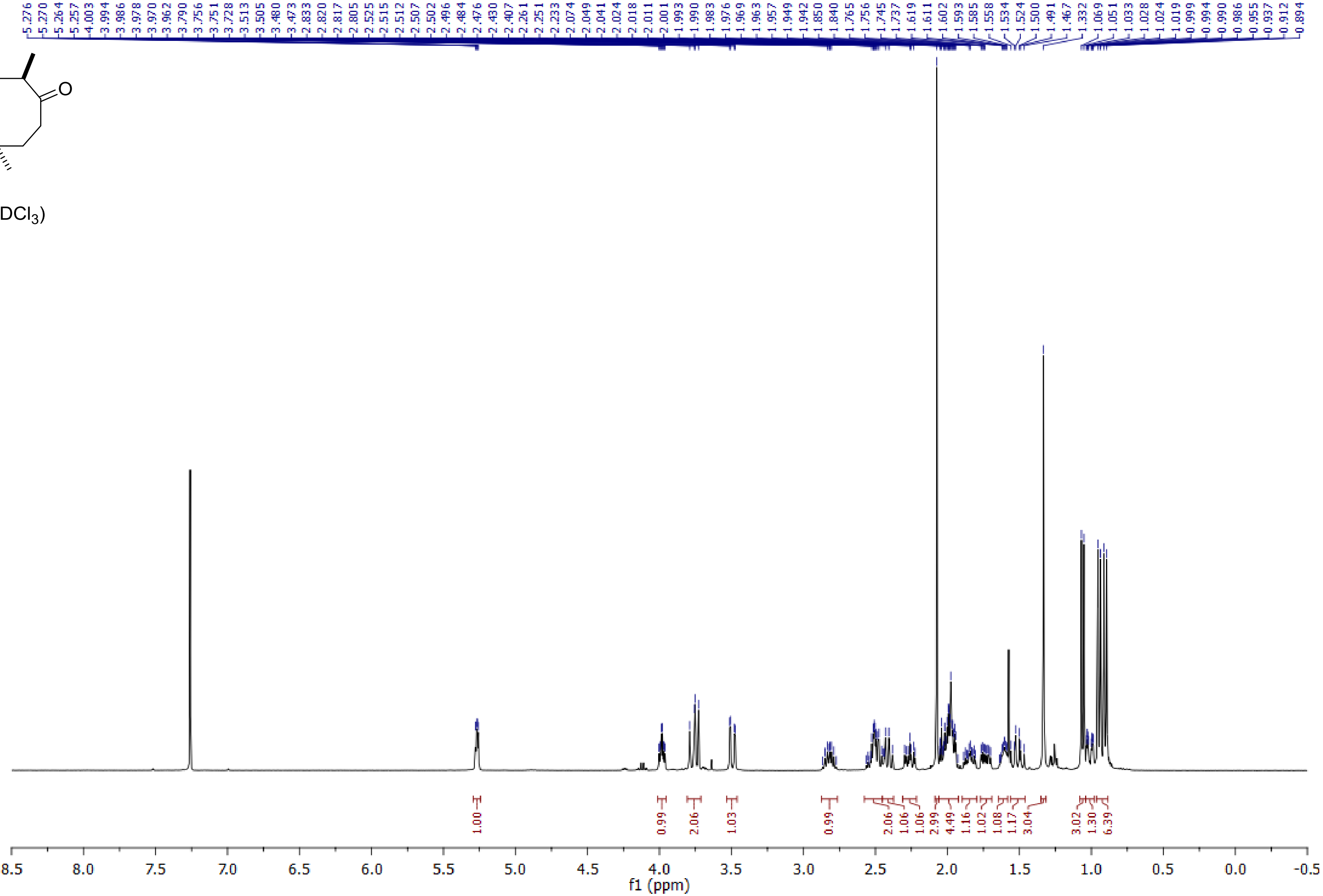

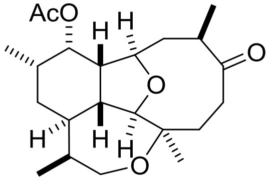

43
(101 MHz, $CDCl_3$)

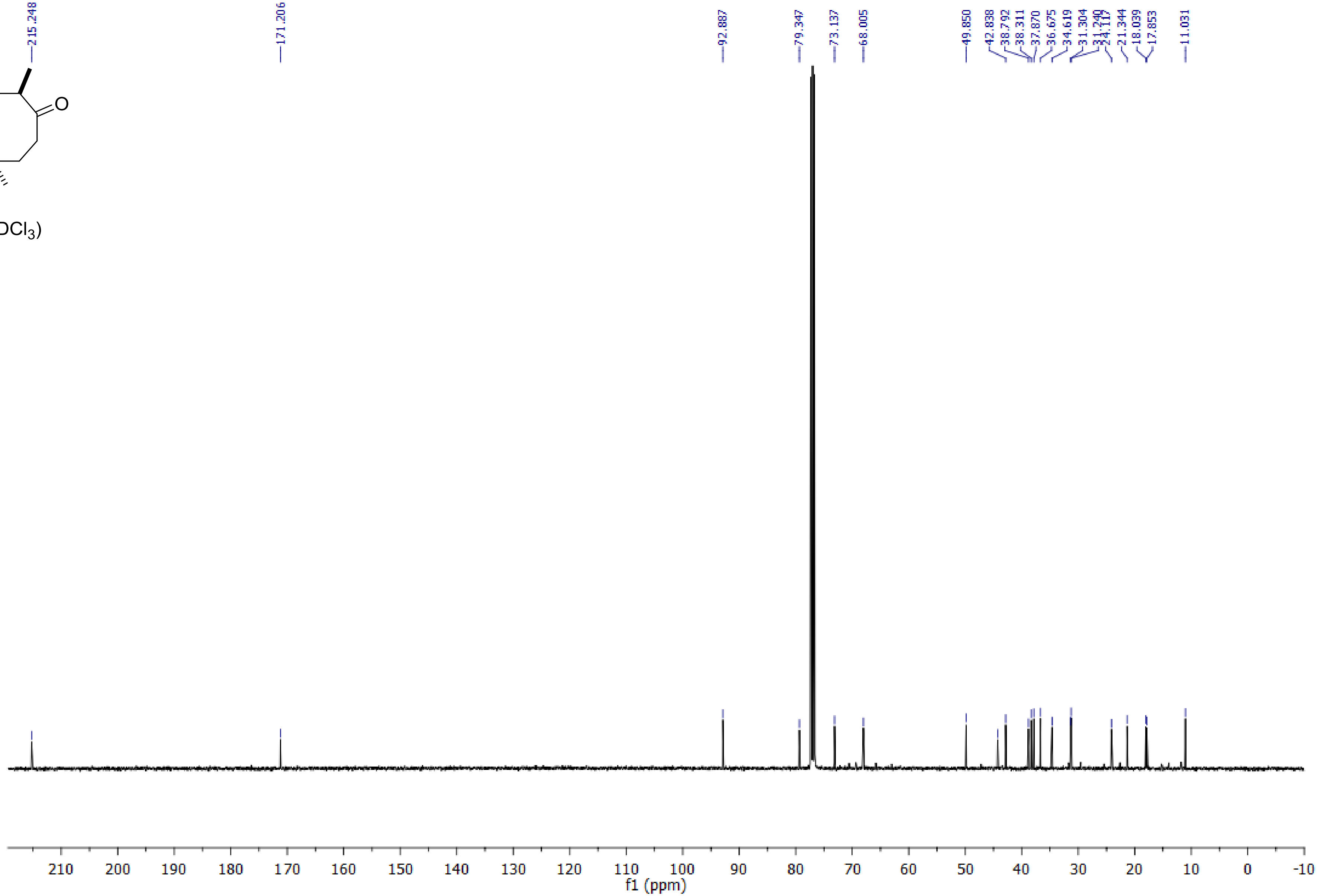

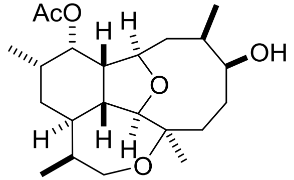

44
(500 MHz, $CDCl_3$)

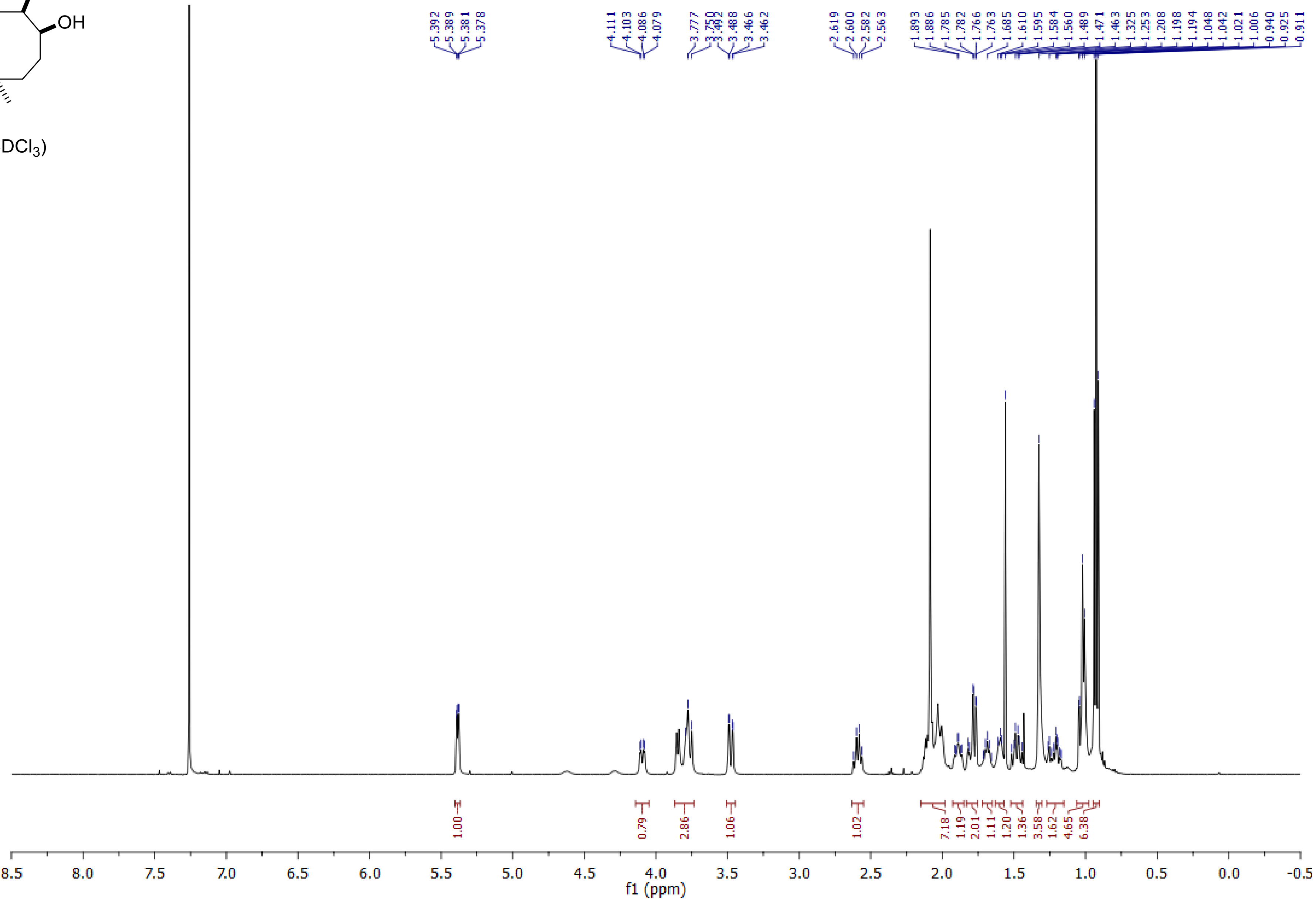

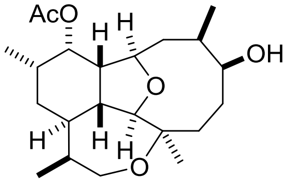

44
(126 MHz, $CDCl_3$)

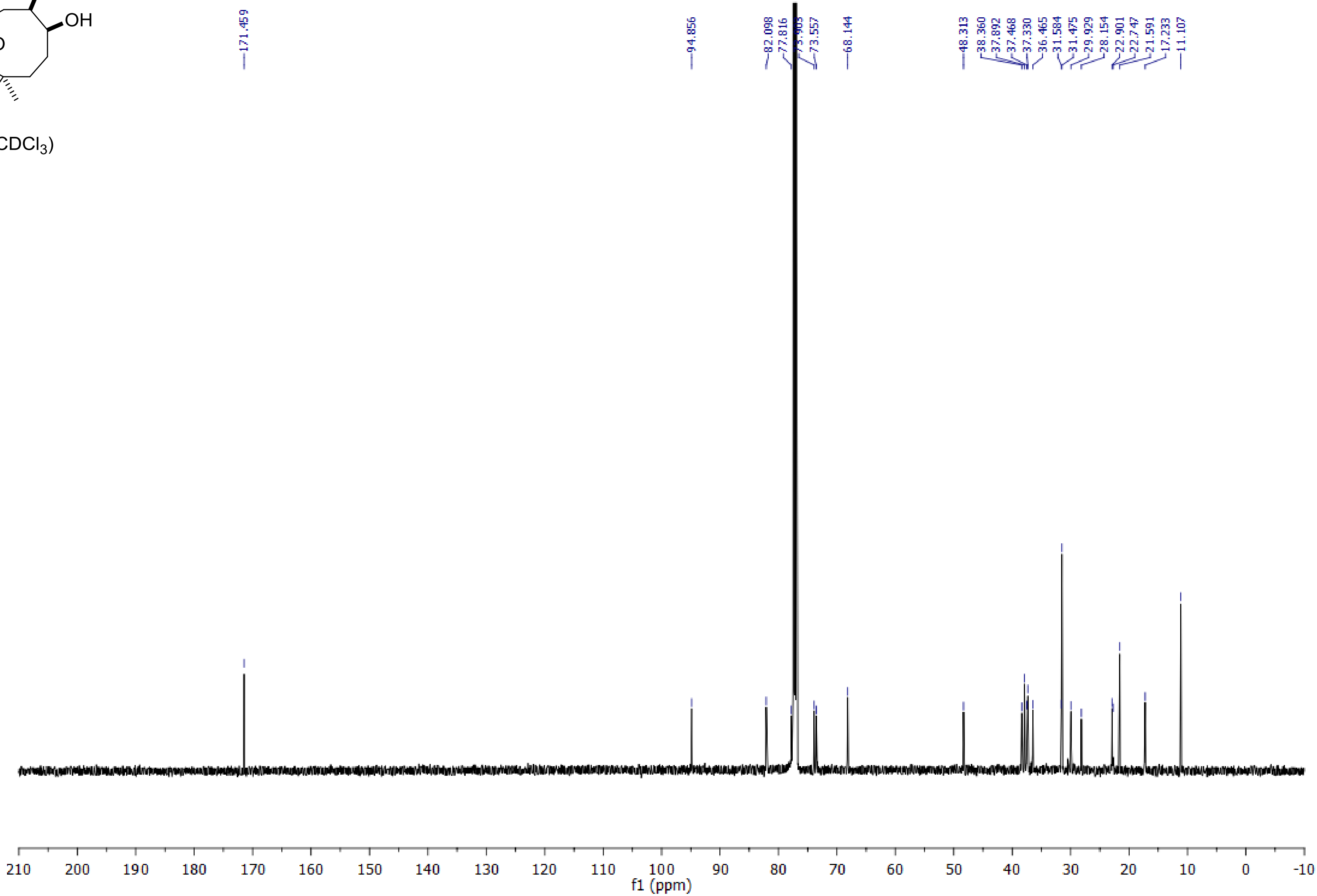

Supplement: Supplementary file 1 — Supplementary [file CHEM-26-1155-s001.pdf]
